# Channeling C1 Metabolism toward *S*-Adenosylmethionine-Dependent Conversion of Estrogens to Androgens in Estrogen-Degrading Bacteria

**DOI:** 10.1128/mBio.01259-20

**Published:** 2020-08-25

**Authors:** Christian Jacoby, Joris Krull, Jennifer Andexer, Nico Jehmlich, Martin von Bergen, Thomas Brüls, Matthias Boll

**Affiliations:** a Faculty of Biology-Microbiology, Albert-Ludwigs-Universität Freiburg, Freiburg im Breisgau, Germany; b Insitute of Pharmaceutical Sciences, Albert-Ludwigs-Universität Freiburg, Freiburg im Breisgau, Germany; c Department of Molecular Systems Biology, Helmholtz Centre of Environmental Research-UFZ, Leipzig, Germany; d Institute of Biochemistry, Faculty of Life Sciences, University of Leipzig, Leipzig, Germany; e CEA, DRF, Institut François Jacob-Genoscope, Evry, France; f CNRS-UMR8030, Génomique Métabolique, Université Paris-Saclay, Evry, France; University of California, Irvine

**Keywords:** C1 metabolism, *S*-adenosylmethionine, bioremediation, estrogen, methyltransferase, vitamin B12

## Abstract

Bacterial degradation of endocrine disrupting and carcinogenic estrogens is essential for their elimination from the environment. Recent studies of the denitrifying, estrogen-degrading *Denitratisoma* strain DHT3 revealed the conversion of estrogens to androgens by a putative cobalamin-dependent methyltransferase encoded by the *emtABCD* genes. The methyl donor and its continuous regeneration to initiate estradiol catabolism have remained unknown. Here, large-scale cultivation of the denitrifying bacterium Denitratisoma oestradiolicum with estrogen provided the biomass required for quantitative biochemical analyses. Soluble fractions of extracts from estradiol-grown cells catalyzed the *S*-adenosyl-l-methionine (SAM)- and Ti(III)-citrate-dependent conversion of 17β-estradiol/estrone to the respective androgens at 0.15 nmol min^−1 ^mg^−1^. Kinetic studies of 17β-estradiol methylation and reverse 1-dehydrotestosterone demethylation reactions indicated that the exergonic methyl transfer from SAM to the putative cobalamin drives the endergonic methyl transfer from the methylcobalamin intermediate to the phenolic ring A. Based on a high-quality circular genome from D. oestradiolicum, proteogenomic analyses identified a 17β-estradiol-induced gene cluster comprising *emtABCD* genes together with genes involved in SAM regeneration via l-serine and l-methionine. Consistent with this finding, l-methionine/ATP or l-serine/ATP/tetrahydrofolate/l-homocysteine substituted for SAM as methyl donors, further confirmed by the incorporation of the ^13^C-methyl-group from ^13^C-l-methonine into methyl(III)cobalamine and the estrone methylation product androsta-1,4-diene-3-one. This work demonstrates that during bacterial estrogen catabolism, the C1 pool is channeled toward the initiating methyl transfer to ring A. The effective cellular SAM regeneration system may serve as a model for whole-cell SAM-dependent methylation reactions of biotechnological interest.

## Abstract (precis)

Estrogens comprise a group of related hormones occurring in predominantly female vertebrates, with endocrine disrupting and carcinogenic potential. Microbial biodegradation of estrogens is essential for their elimination from surface waters and wastewater. Aerobic bacteria employ oxygenases for the initial cleavage of the aromatic ring A. In contrast, anaerobic degradation of estrogens is initiated by methyl transfer-dependent conversion into androgens involving a putative cobalamin-dependent methyltransferase system. The methyl donor for this unprecedented reaction and its stoichiometric regeneration have remained unknown. With the biomass obtained from large-scale fermentation of an estrogen-degrading denitrifying bacterium, we identified *S*-adenosyl-methionine (SAM) as the methyl donor for the cobalamin-mediated methyl transfer to estrogens. To continuously supply C1 units to initiate estrogen degradation, genes for SAM regeneration from estradiol-derived catabolites are highly upregulated. Data presented here shed light into biochemical processes involved in the globally important microbial degradation of estrogens.

## INTRODUCTION

**FIG 1:**
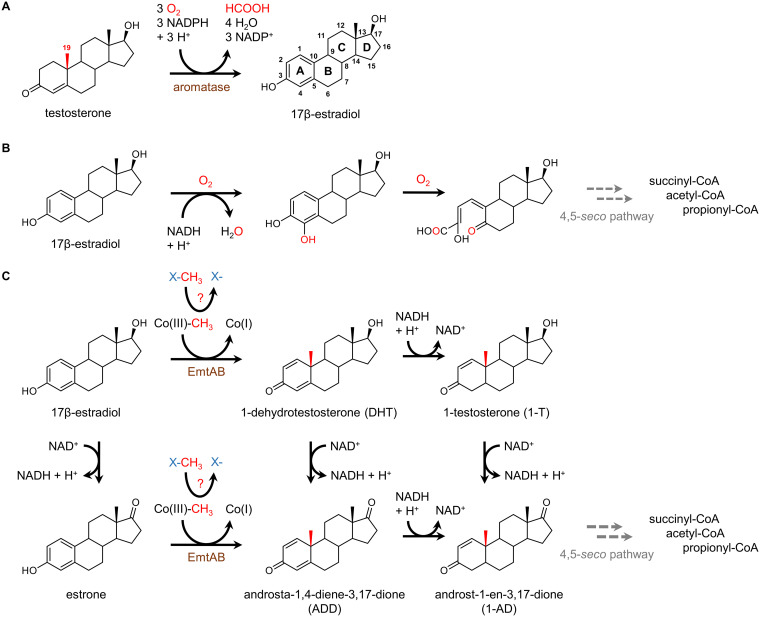
Biosynthesis in vertebrates and bacterial degradation of 17β-estradiol. (A) O_2_- and NADPH-dependent aromatization of testosterone to 17β-estradiol in vertebrates catalyzed by Cyp450 aromatases. (B) Aerobic bacterial estradiol degradation involving mono- and dioxygenase-dependent reactions. (C) Proposed 17β-estradiol degradation in denitrifying bacteria initiated by cobalamin-mediated methyl transfer resulting in the retroconversion of 17β-estradiol to 1-dehydrotestosterone that may be reduced to 1-testosterone or oxidized to androsta-1,4-diene-3,17-dione (ADD); likewise, estrone is methylated to ADD. All androgen intermediates are converted to androst-1-ene-3,17-dione (1-AD), the common intermediate of the 2,3-*seco* pathway. EmtAB, the proposed subunits of estrogen methyl transferase.

Estrogens are cholesterol-derived hormones that are involved in a large number of developmental, physiological, and reproductive functions in predominantly female animals, including humans (1). They are composed of a tetracyclic carbon skeleton with a phenolic ring A. Natural estrogens include estrone, 17β-estradiol (estradiol), and estratriol. The synthetic 17α-ethynyl-estradiol is a component of birth control pills. Biosynthesis of estradiol from testosterone proceeds via the O_2_- and NADPH-dependent oxidation of the angular C19 methyl functionality by cytochrome p450 aromatases (e.g., CYP19A1) (Fig. 1A) (2). During this reaction, the C19 methyl group is released as formate, coupled to the aromatization of ring A.

Once released into the environment from sewage treatment plants, livestock feedlots, or hospital effluents, estrogens cause adverse effects on aquatic life, e.g., by affecting physiology, behavior, and sexual development of fish (3–7). Moreover, estrogens are classified as group 1 carcinogens by the World Health Organization (https://monographs.iarc.fr/list-of-classifications/). Due to these negative attributes, their abundance in the environment is of global importance. Biodegradation is considered the major process for the elimination of estrogens from the environment (7–9). Complete biodegradation of estrogens is only accomplished by microorganisms, and is mainly hampered by their low solubility in water (e.g., 1.5 mg liter^−1^ for estradiol) (10).

Several aerobic estrogen-degrading bacteria have been isolated (9), but knowledge of the pathways, enzymes, and genes involved has remained surprisingly limited for a long time. In 2017, Wang et al. (11) reported that estradiol degradation in the aerobic alphaproteobacterium *Sphingomonas* strain KC8 is initiated by the dehydrogenation to estrone, followed by hydroxylation and cleavage of ring A (Fig. 1B). A specific ring-cleaving 4-hydroxyestrone 4,5-dioxygenase was isolated and, together with additional studies (12), the so-called 4,5-*seco* pathway of aerobic estrogen degradation was established. The further degradation of the A/B rings is still elusive, whereas the degradation of the C/D rings was suggested to proceed in a way similar to the degradation of cholesterol or androgens with 3aα-H-4α(3′-propanoate)-7αβ-methylhexadydro-1,5-indanedione (HIP) as a key intermediate (13).

Until very recently, even less was known about estrogen degradation in anaerobic bacteria. So far, three betaproteobacteria have been reported to use estrogens as carbon and, together with nitrate, as an energy source: Denitratisoma oestradiolicum DSM 16959 (14), *Denitratisoma* sp. strain DHT3 (15), and Steroidobacter denitrificans (16). In the pioneering study with *Denitratisoma* sp. strain DHT3, metabolite analyses revealed that estrogens such as estrone or estradiol are initially converted to androgens by methyl transfer to C10 (15). Multiple findings suggested that this methyl transfer is mediated by an unprecedented cobalamin-dependent methyltransferase complex encoded by the *emtABCD* genes (estradiol methyl transferase) (15) (Fig. 1C). Notably, similar genes are also present in *D. oestradiolicum* and S. denitrificans (14, 16). Sequence analyses suggested that *emtA* codes for the catalytic subunit of a methyltransferase related to monomethylamine methyltransferase MtmB from methanogenic archaea (17). It lacks the amber stop codon-encoded pyrrolysine that is typically found in archeal methylamine methyltransferases (18). EmtB is proposed to bind a corrinoid cofactor, whereas EmtC shows no similarities to known proteins. EmtD is similar to oxidoreductases possibly involved in the reductive regeneration of the active cob(I)-alamin state of the cofactor. Cob(I)amid cofactors sporadically oxidize to the inactive Co(II) state, which can be reactivated by ATP- (19–21) or SAM-dependent (22) electron transfer. In the vicinity of the *emtABCD* genes, a cluster of three further genes was assumed to play a role in ATP-dependent reductive regeneration of the cob(I)alamin state. It included a putative RACE-like protein (reductive activator of corrinoid enzyme) (19–21, 23). The 1-dehydrotestosterone (DHT) formed from estradiol is subsequently degraded by oxygen-independent reactions of the 2,3-*seco*-pathway, as evidenced by the identification of the corresponding genes in the genome of strain DHT3 (15). This pathway was established for anaerobic cholesterol and androgen degradation (24–26).

The milestone discovery of methyl transfer-dependent retroconversion of estrogens into androgens gave rise to a number of fundamental questions. In particular, the source of the methyl group and its continuous regeneration for initiating oxygen-independent estrogen degradation have remained unclear. Further, the proposed unprecedented methyl transfer from methylcobalamin to C10 of estrogen, coupled to dearomatization, is thermodynamically unfavorable. As understood so far, methyl transfer from methylcobalamin usually requires strong nucleophiles as methyl acceptors, such as thiols (l-homocysteine/coenzyme M), R-NH_2_ (tetrahydrofolate, THF), or Ni(I) (A-cluster in acetyl-CoA synthase) (22, 27–29). Here, we identified SAM as the stoichiometric methyl group donor for cobalamin-mediated estrogen methylation; its continuous regeneration is sustained by the upregulation of C1 metabolism and the channeling of its metabolites toward estrogen catabolism.

## RESULTS

### Large-scale cultivation of Denitratisoma oestradiolicum for quantitative *in vitro* assays.

In the previous study by Wang et al. (15), the conversion of estrogens to androgens in strain DHT3 was demonstrated in whole-cell suspensions and cell extracts. In assays with the latter, the limiting biomass available allowed solely qualitative endpoint determination of product formation after 1.5 h of incubation. The observed ATP dependence of estrogen methylation by cell extracts was assigned to the reductive reconstitution of an active cob(I)amide from an inactive cob(II)amide, probably catalyzed by a RACE-like protein (19, 20). The observed slightly stimulating, but nonstoichiometric effect of SAM on estrogen methylation was explained by catalytic reconstitution of methylcob(III)amide as described for methionine synthase (30). The proposed product of estradiol methylation, DHT, was not found in *in vitro* assays. Instead, androgen products with fully reduced ring A were identified, which, however, are not intermediates of the proposed 2,3-*seco* pathway (24, 25). In summary, the nature of the product, the source of the stoichiometric methyl donor, and the cob(II)alamin activation system of estrogen methylation were at issue.

To address these open questions by quantitative biochemical approaches, *D. oestradiolicum* was chosen as a model organism for anaerobic estrogen degradation. In preliminary cultivation trials with estradiol (2 mM) and nitrate (15 mM) as carbon and energy sources, the bacterium reached higher densities compared to strain DHT3 (15). After large-scale cultivation in a 200-liter fermenter with a doubling time of 16 h in the early exponential phase, 122 g of cells (wet mass) was obtained, with 30 ± 5 mg protein per g wet cell mass (for the growth curve see Fig. S1 in the supplemental material). This served as biomass for the biochemical assays.

10.1128/mBio.01259-20.1 FIG S1 Growth of *D. oestradiolicum* with 2 mM 17β-estradiol and 10 mM nitrate in a 200-liter fermenter. Growth was determined by OD_578_ measurements after removal of 17β-estradiol by low-spin centrifugation (800 × *g*, 2 min and 4°C). A wet mass of 122 g was obtained. Download FIG S1, TIF file, 0.2 MB. Copyright © 2020 Jacoby et al. 2020 Jacoby et al. This content is distributed under the terms of the Creative Commons Attribution 4.0 International license.

### SAM-dependent methylation of estrogens to androgens.

**FIG 2:**
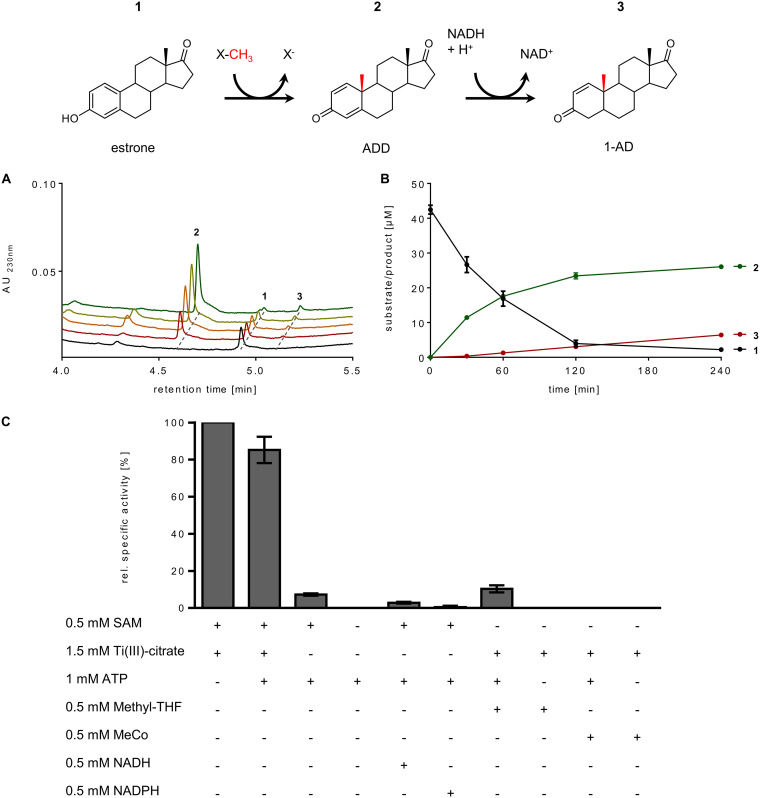
*In vitro* conversion of estrone to androsta-1,4-diene-3-one (ADD) by cell extracts. (A) UPLC analyses of estrone (0.1 mM) to ADD conversion by cell extracts of *D. oestradiolicum* in the presence of 0.5 mM SAM and 1.5 mM Ti(III)-citrate. ADD was slowly further reduced by traces of NADH present in extracts. (B) Time-dependent product and coproduct formation. (C) Methyl- and electron-donor dependence of estrone methylation on cosubstrates (100% activity corresponds to 0.15 ± 0.004 nmol min^−1 ^mg^−1^).

We tested the effect of various potential cosubstrates involved in methyl transfer and cob(I)alamin reconstitution by cell extracts comprising SAM (0.5 mM), N^5^-methyl-THF (0.5 mM), and methylcobalamin (0.5 mM) as potential methyl donors, and Ti(III)-citrate (1.5 mM), NAD(P)H (0.5 mM), and ATP (1 mM) as components involved in the reduction of the accidentally formed inactive cob(II)alamin. Strictly depending on SAM and Ti(III)-citrate, the time- and cell extract-dependent conversion of estradiol (50 μM) to DHT and a number of downstream products was observed by ultraperformance liquid chromatography (UPLC) analyses at a rate of 0.15 ± 0.01 nmol min^−1 ^mg^−1^ (Fig. S2 in the supplemental material). The formation of estrone, androsta-1,4-diene-3-one (ADD), and androst-1-en-3,17-dione (1-AD) can be rationalized by the presence of catalytic amounts of NAD^+^ in the cell extracts that serves as electron acceptor for the oxidation of the C-17 hydroxyl groups of estradiol and DHT to estrone and ADD, respectively. The NADH formed in this reaction is then used for the reduction of ADD to 1-AD and DHT to 1-testosterone (1-T), respectively, which regenerates NAD^+^ (Fig. 1C). To minimize the formation of downstream products, estradiol was replaced by estrone as the substrate that was converted at a virtually identical rate. In these assays, ADD was identified as the main product and 1-AD as the minor second product, whereas formation of further downstream products was negligible (Fig. 2A and B). Substitution of the artificial electron donor Ti(III)-citrate by the substrates of a putative RACE component, ATP and NAD(P)H, resulted in only a marginal methyl transfer rate. When SAM was replaced by the alternative methyl donors N^5^-methyl-THF or methylcobalamin, virtually no conversion of estradiol was observed. However, when ATP was added in combination with N^5^-methyl-THF, maximally 10% of the activity was recovered. These results suggest that SAM functions as the methyl donor for estradiol methylation and that a low-potential electron donor is essential for keeping the cofactor of estradiol methyltransferase (EMT) in the active cob(I)alamin state. The slight stimulating effect of ATP in assays with N^5^-methyl-THF suggests that the latter may serve as methyl donor for SAM synthesis via the cobalamin-containing methionine synthase and ATP-dependent methionine adenosyltransferase, providing that catalytic amounts of SAM or precursors of it, such as l-methionine, l-homocysteine, l-serine, etc., were present in the extracts used.

10.1128/mBio.01259-20.2 FIG S2 *In vitro* conversion of 17β-estradiol to dehydrotestosterone (DHT) and downstream products by cell-free extracts of *D. oestradiolicum* in the presence of 0.5 mM SAM and 1.5 mM Ti(III)-citrate. (B) Time-dependent product and coproduct formation. The following retention times were observed: 17β-estradiol (1), 4.57 min; estrone (2), 4.92 min; DHT (3), 4.33 min; ADD (4), 4.57 min; 1-testosterone (5), 4.94 min; 1-AD (6), 5.10 min. Download FIG S2, TIF file, 1.2 MB. Copyright © 2020 Jacoby et al. 2020 Jacoby et al. This content is distributed under the terms of the Creative Commons Attribution 4.0 International license.

In previous *in vitro* assays with strain DHT3, androgens with a fully reduced ring A in place of DHT were identified (15). The latter is an intermediate of the testosterone catabolic 2,3-*seco* pathway (24, 25), whereas androgens with a fully reduced ring A are not. They most likely arose as side products formed by an unspecific dehydrogenase. Such side reactions were not observed in the *in vitro* assays with extracts from *D. oestradiolicum* grown with estradiol.

### Kinetic parameters, stoichiometry, and inhibition of estrogen methylation.

**FIG 3:**
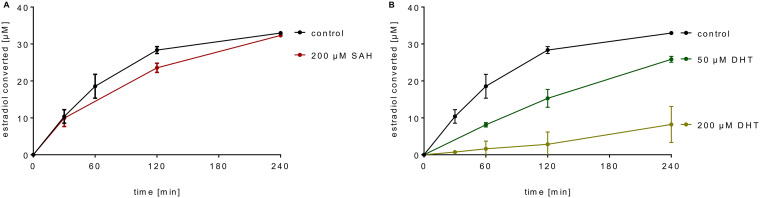
Product inhibition of the estrogen methyltransferase. Time- and SAM-dependent conversion of estradiol by cell extracts in the presence of the products *S*-adenosylhomocysteine (SAH) (A) and 1-dehydrotestosterone (DHT) (B).

The *K*_m_-values of the methyltransferase for estradiol and estrone were 35 ± 4 and 14 ± 3 μM, respectively (for Michaelis-Menten curves see Fig. S3 in the supplemental material). For determining the stoichiometry of methyl transfer from SAM to estrone, cell extracts were subjected to ammonium sulfate precipitation to remove nonproteinaceous molecules that may interfere with the determination of products. Using the solubilized and washed methyltransferase activity containing 70% ammonium sulfate fraction, the conversion of SAM to *S*-adenosyl-l-homocysteine (SAH) and estrone to ADD was followed with 0.8 mol SAH per mol ADD formed (Fig. S4 in the supplemental material). As SAM-dependent methyltransferases are typically inhibited by the product SAH (31), we tested the effect of SAH on SAM-dependent estradiol methylation. Even in the presence of 200 μM SAH, no significant inhibition was observed (Fig. 3A); SAH degradation by other enzymes was negligible in the course of the reaction. Instead, the product DHT inhibited estradiol methylation in a dose-dependent manner (Fig. 3B).

10.1128/mBio.01259-20.3 FIG S3 Michaelis-Menten curves for *K*_m_ value determination of estrone and 17β-estradiol. Download FIG S3, TIF file, 0.3 MB. Copyright © 2020 Jacoby et al. 2020 Jacoby et al. This content is distributed under the terms of the Creative Commons Attribution 4.0 International license.

10.1128/mBio.01259-20.4 FIG S4 Stoichiometry of ADD and SAH formation during SAM-dependent conversion of estrone (50 μM) to ADD by cell-free extracts. The amount of SAH and ADD was determined via UPLC-based analysis. Download FIG S4, TIF file, 0.2 MB. Copyright © 2020 Jacoby et al. 2020 Jacoby et al. This content is distributed under the terms of the Creative Commons Attribution 4.0 International license.

### Partial methyl transfer activities and the reverse reaction.

**FIG 4:**
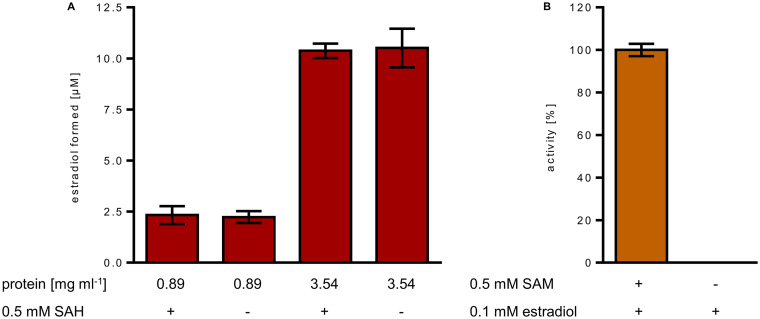
Analysis of partial methyl transfer reactions of estrogen methyltransferase (EMT). (A) UPLC analysis of estradiol formation from DHT (0.1 mM) by the reverse demethylation activity of the 70% ammonium sulfate precipitation protein fraction from cell extracts. SAH had no stimulating effect, indicating that no methyl transfer from DHT to SAH occurred. (B) UPLC analysis of EMT activities of the “methylated” protein fraction obtained after incubation with SAM in the absence of estradiol. After removal of excess SAM, the methylated protein component formed was not capable of estradiol methylation. In a control, this fraction again showed EMT activity after addition of SAM.

Cell-free extracts catalyzed the reverse demethylation of DHT to estradiol (Fig. 4A). By increasing the amount of cell extract 4-fold, the amount of estradiol formed from DHT increased likewise from 2.3 ± 0.4 μM to 10.4 ± 0.3 μM. This result suggests a stoichiometric methyl transfer from DHT to an abundant proteinaceous acceptor, most likely the corrinoid protein of EMT, with 2.8 ± 0.3 nmol methyl group transferred per mg protein in the cell extract. Addition of SAH did not increase the amount of estradiol formed from DHT further, indicating that the proposed methylcobalamin intermediate formed from DHT is not competent for SAH methylation. To test whether the proposed methylcobalamin intermediate is competent for methyl transfer to estradiol (forward reaction), we incubated extract with either SAM or DHT in the absence of estradiol, and washed the SAM- or DHT-methylated extract by ammonium sulfate precipitation to remove excess of SAM/DHT or other potential low-molecular methyl donors. Using these extracts, virtually no methyl transfer to estradiol was observed. In a control experiment, this extract catalyzed SAM-dependent estrogen methylation at the expected rate (Fig. 4B). Hence, the proposed methylcob(III)alamin intermediate was incompetent for methyl transfer to estradiol in the absence of SAM. In summary, we conclude that exergonic SAM-dependent methylation of the cob(I)alamin cofactor of EMT drives the unfavorable methyl transfer from the methylcob(III)alamin intermediate to estradiol forward.

### High-quality circular genome of *D. oestradiolicum* DSM 16959.

In the recent study by Chen et al. (32), a draft genome of *D. oestradiolicum* DSM 16959 was generated based on short read Illumina sequencing. Though the authors produced a high-quality sequence for strain DHT3 by leveraging long read sequencing using the nanopore technology, their sequence for strain DSM 16959 was left highly fragmented into 67 contigs (65 scaffolds). To provide a basis for extensive gene neighborhood investigations and to support high quality proteogenomic analyses, we generated a high-quality genomic sequence for *D. oestradiolicum* DSM 16959 through a hybrid approach combining both long and short reads. A single contig corresponding to the main bacterial chromosome was obtained, encompassing 4168563 bases (62% GC content) and harboring 3,986 coding sequences. The sequence of a 124849-base plasmid (63% GC content), unnoticed in the earlier draft assembly and encompassing 187 coding sequences, was reconstructed. Both sequences are available from the ENA/NCBI/DDBJ databases under accession numbers LR778301 and LR778302, respectively (study identifier PRJEB37013). Beyond gene neighborhood analyses, the availability of a fully contiguous genome sequence enables comparative genomics investigations, e.g., the identification of close genomes based on genome-level gene syntenies (Table S1 in the supplemental material). In particular, a direct comparison of the DHT3 and DSM 16959 genomes in terms of conserved gene synteny groups (syntons) highlighted significant differences in genome organization between the two strains (Fig. S5 in the supplemental material), consistent with the noticeable difference in genome length (around 500 kb).

10.1128/mBio.01259-20.5 FIG S5 Conserved synteny between *D. oestradiolicum* (upper) and *Denitratisoma* sp. DHT3 (bottom). Only synteny groups (syntons) harboring 10 or more genes are displayed. Pink: transposases and insertion sequences; blue: rRNA; green: tRNA. Inversions around the origin of replication are shown in red. Download FIG S5, TIF file, 2.2 MB. Copyright © 2020 Jacoby et al. 2020 Jacoby et al. This content is distributed under the terms of the Creative Commons Attribution 4.0 International license.

10.1128/mBio.01259-20.7 TABLE S1 Genome-level synteny statistics for *D. oestradiolicum* (top 20 closest genomes). Putative orthologous relations between two genomes were defined as gene pairs satisfying the bi-directional best hit (BBH) criterion, or a blastP alignment threshold, with a minimum of 35% sequence identity on 80% of the length of the smallest protein. These relations were subsequently used to search for conserved gene clusters, e.g., synteny groups (syntons) among several bacterial genomes. All possible kinds of chromosomal rearrangements were allowed (inversion, insertion/deletion). A gap parameter, representing the maximum number of consecutive genes which were not involved in a synteny group, was set to five genes. Download Table S1, DOCX file, 0.02 MB. Copyright © 2020 Jacoby et al. 2020 Jacoby et al. This content is distributed under the terms of the Creative Commons Attribution 4.0 International license.

### Proteogenomic analysis of anaerobic estrogen degradation.

**FIG 5:**
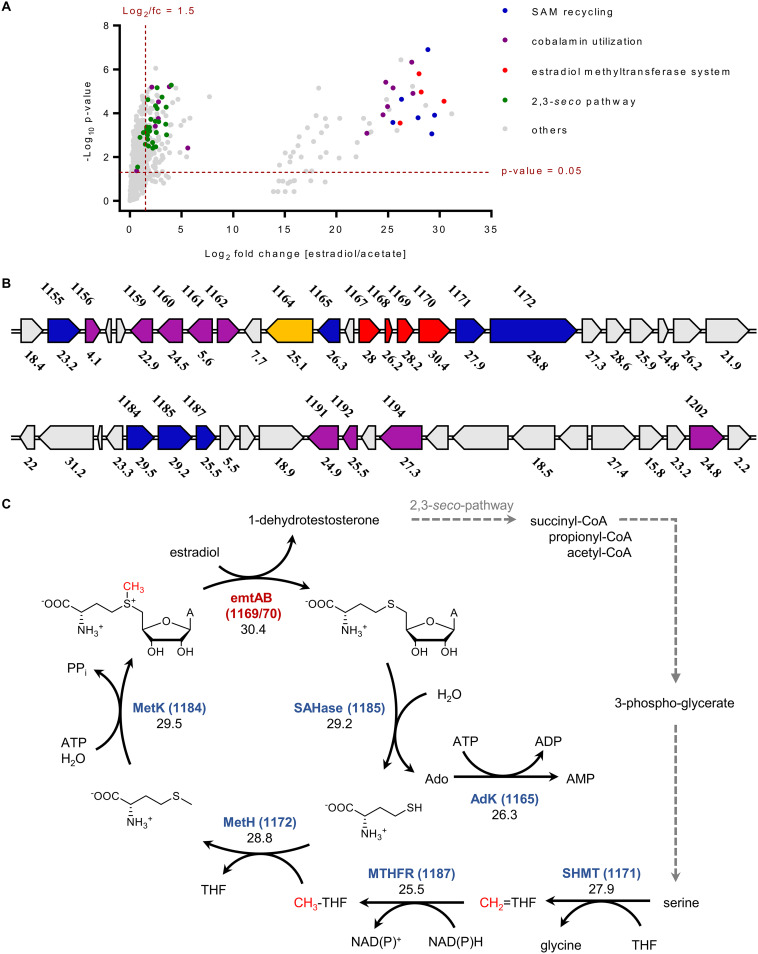
Differential proteome analysis indicates an estradiol-induced gene cluster involved in estradiol methylation/SAM recycling. (A) Volcano-plot of overall proteomic data (see Table S2 in the supplemental material). Genes were assigned to functions based either on their previous experimental identification or on their higher abundance in estradiol- versus acetate-grown cells, together with similarities to reported genes. The best hits are summarized in Table S3. (B) Estradiol-induced gene cluster comprising genes encoding SAM-regeneration proteins (blue), EMT (red), cobamide utilization/synthesis proteins (purple), and a putative RACE (orange). The numbers shown above the genes refer to gene annotation in the genome of *D. oestradiolicum* (DENOEST_v1_XXXX), the numbers below to the abundance fold changes in cells grown with estradiol versus acetate. (C) The deduced pathway for directing central C1 metabolism to estradiol methylation by estradiol-induced gene products with fold changes in cells grown with estradiol versus acetate. Abbreviations: EmtAB, estrogen methyltransferase; SAHase, *S*-adenosylhomocysteinase; MetH, l-methionine synthase; MetK, l-methionine adenosyltransferase; MTHFR, methylene-THF reductase; SHMT, l-serine hydroxymethyltransferase.

To identify estrogen-induced genes in *D. oestradiolicum* involved in the initial methyl transfer reaction, methyl donor regeneration, and sterane skeleton degradation, the proteome of *D. oestradiolicum* cells grown with estradiol was compared with that from cells grown with acetate (Fig. 5, for original data, see Table S2 in the supplemental material). Proteome analyses of biological triplicates for each growth substrate revealed high reliability of the data obtained (Fig. 5A). Genes involved in estrogen catabolism were categorized according to their function in estrogen activation (Fig. 5B), rings AB, and rings CD degradation (Fig. S6 in the supplemental material). They were assigned to these functions based on their higher abundance in estrogen- versus acetate-grown *D. oestradiolicum*, and on clear similarities with experimentally verified gene products (up to 98% amino acid sequence similarity). The best hits with previously identified genes from betaproteobacterial, gammaproteobacterial, or actinobacterial steroid degraders are summarized in Table S3 in the supplemental material.

10.1128/mBio.01259-20.6 FIG S6 Gene clusters putatively involved in rings AB and rings CD degradation during anaerobic growth of *D. oestradiolicum* with 17β-estradiol/nitrate. The numbers above the gene arrows refer to gene identification numbers in the genome of *D. oestradiolicum*, the numbers below to differential abundances (log2 ratios) in cells grown with 17β-estradiol versus acetate. Download FIG S6, TIF file, 0.5 MB. Copyright © 2020 Jacoby et al. 2020 Jacoby et al. This content is distributed under the terms of the Creative Commons Attribution 4.0 International license.

10.1128/mBio.01259-20.8 TABLE S2 Original data set of differential proteome analyses. Abundances of gene products, log2 ratios and *P* values for proteins are listed and compared in cells grown with 17β-estradiol/nitrate versus acetate/nitrate. Download Table S2, DOCX file, 0.1 MB. Copyright © 2020 Jacoby et al. 2020 Jacoby et al. This content is distributed under the terms of the Creative Commons Attribution 4.0 International license.

10.1128/mBio.01259-20.9 TABLE S3 List of differentially regulated genes in *D. oestradiolicum*; log2 ratios for protein abundances during growth with 17β-estradiol/nitrate and acetate/nitrate are shown. Similarities of differentially abundant gene products to those from other organisms are indicated. Download Table S3, DOCX file, 0.05 MB. Copyright © 2020 Jacoby et al. 2020 Jacoby et al. This content is distributed under the terms of the Creative Commons Attribution 4.0 International license.

In accordance with previous transcriptome analyses (15), the *EmtABCD* gene products (DENOEST_v1_1167-1170), and a RACE-like protein (DENOEST_v1_1164) were clearly more abundant in estradiol- versus acetate-grown cells (log_2_ > 25) (Fig. 5). In addition, a number of gene products involved in SAM regeneration were identified at higher abundances in estradiol- versus acetate-grown cells, including adenosyl-l-homocysteinase, adenosine kinase, l-serine hydroxymethyltransferase, N^5^,N^10^-methylene-THF reductase, methionine synthase, and methionine adenosyltransferase (log_2_ > 25, Tables S3 and S4 in the supplemental material). The genes are located in the vicinity of the *emtABCD* genes and share up to 98% amino acid sequence identity to homologous genes in the genome of strain DHT3. The identification of these estrogen-induced genes escaped detection during previous transcriptome analyses in strain DHT3. This finding may be explained by the fact that we compared cells grown with estradiol versus acetate and not estradiol versus testosterone. Second copies of genes involved in regeneration of SAM from l-serine and l-methionine were identified in the genome of *D. oestradiolicum* and were equally abundant in cells grown with estradiol and acetate (Table S4 in the supplemental material), suggesting that these second copies are involved in housekeeping C1 metabolism. In agreement with the transcriptome analyses in DHT3 (15), the abundance of gene products involved in vitamin B_12_ uptake and modification was increased by estradiol in *D. oestradiolicum*, confirming that all anaerobic estradiol degraders known so far depend on B_12_ in the culture medium. These species upregulate genes involved in uptake and processing B_12_-derived precursors to the active cobamide cofactor.

10.1128/mBio.01259-20.10 TABLE S4 Differential abundances of gene products involved in C1-metabolism/SAM regeneration in Denitratisoma oestradiolicum. For each predicted enzyme (based on E values of ≤1 e^−50^ and amino acid sequence identities of ≥30%), two gene copies are present in the genome. The log2 abundances of each gene product are given after proteome analyses of estradiol versus acetate grown cells. Only the genes DENOEST_v1_11165-1187 are higher in abundance in estradiol-grown cells. ND, not detectable. Download Table S4, DOCX file, 0.01 MB. Copyright © 2020 Jacoby et al. 2020 Jacoby et al. This content is distributed under the terms of the Creative Commons Attribution 4.0 International license.

Based on similarities with corresponding genes from other cholesterol or androgen degraders (Table S2 and S3 in the supplemental material), gene products putatively involved in sterane skeleton degradation were identified at higher abundances in *D. oestradiolicum* cells grown with estradiol versus acetate (Fig. S6 in the supplemental material). The corresponding β-oxidation genes are organized in two clusters, with each cluster being assigned to the degradation of rings AB and CD, respectively. The gene products putatively involved in rings AB degradation show high amino acid sequence similarities to those putatively involved in β-oxidation-like reactions from anaerobic steroid degraders, but only minor sequence similarities to those from aerobic degraders (Table S3 in the supplemental material). In contrast, gene products putatively involved in rings CD degradation are also highly similar to genes that have been identified in aerobic steroid degradation (13). These finding are consistent with the general assumption that the degradation of rings AB differs in aerobic and anaerobic steroid-degrading bacteria, whereas rings CD degradation proceeds via similar enzymes (24–26).

### Methyl-^13^C-l-methionine as methyl donor for estrogen and cobalamine methylation.

**FIG 6:**
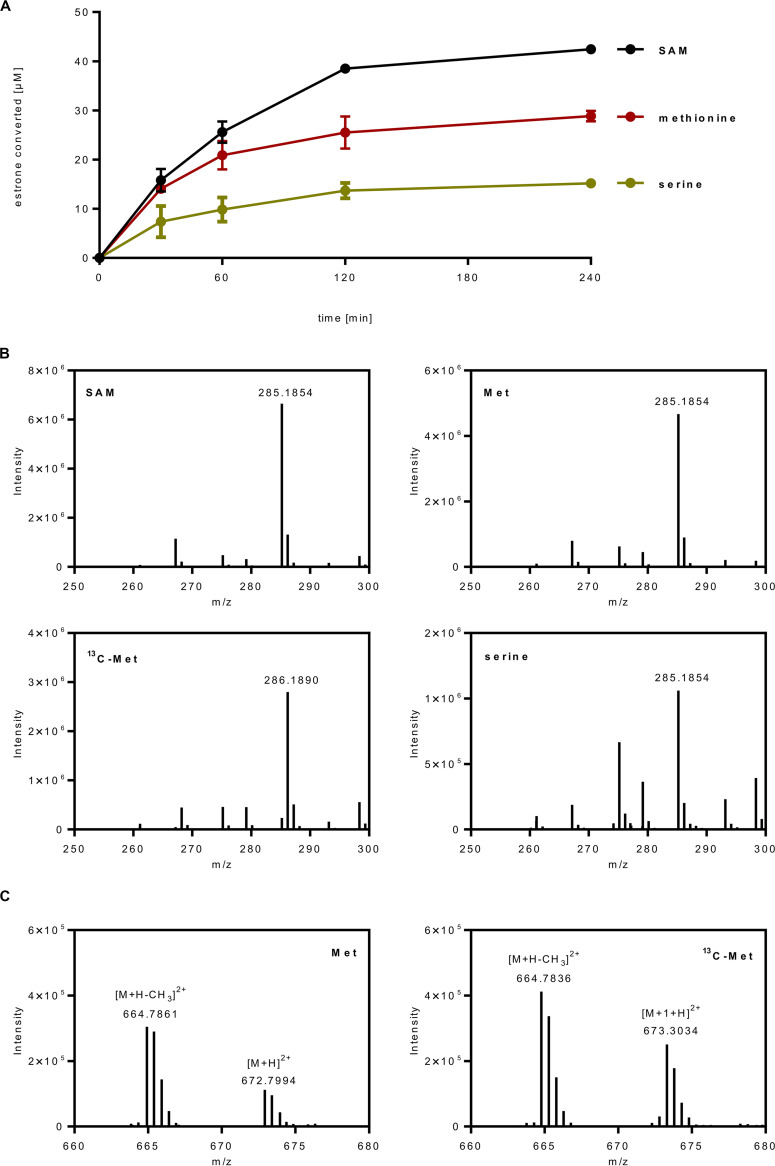
*In vitro* estrone methylation using l-methionine, methyl-^13^C-l-methionine, or l-serine as methyl donors for SAM regeneration. (A) UPLC analysis of the conversion of estrone (0.1 mM) to ADD by cell extracts of *D. oestradiolicum* in the presence of 0.5 mM SAM (black line), 0.5 mM l-methionine and 1 mM ATP (red line), or 0.5 mM l-serine, 1 mM ATP, 0.5 mM THF, and 0.5 mM l-homocysteine (green line). (B) ESI-Q-TOF analysis of the ADD product formed in the *in vitro* assays shown in (A) and in the presence of SAM, methionine (Met), methyl-^13^C-l-methionine (^13^C-Met), and serine. (C) ESI-Q-TOF analysis of the methylcob(III)alamin intermediate in the presence of Met and ^13^C-Met. Due to the doubly charged ions, an *m/z* shift of 0.5 was detected in in the assay with ^13^C-Met; the *m/z* = 664.79 peak is assigned to demethylated methylcob(III)alamin.

Results from differential proteome analyses suggest that SAM used for estradiol methylation is regenerated via l-methionine that is formed by l-methionine synthase from l-homocysteine and N^5^-methyl-THF (Fig. 5C). l-Serine is assumed to serve as the major source for loading THF with a C1 unit by l-serine hydroxymethyltransferase. Based on these findings, we designed *in vitro* assays with cell extracts in which SAM was replaced by either (i) l-methionine/ATP via l-methionine adenosyltransferase, or (ii) by l-serine/ATP/THF/NADH via l-methionine adenosyltransferase, N^5^,N^10^-methylene-THF reductase, and l-serine hydroxymethyltransferase. We determined the time- and cell extract-dependent conversion of estrone in the absence of SAM with both setups, at rates of 0.14 and 0.07 nmol min^−1 ^mg^−1^, respectively (Fig. 6A). Ultraperformance liquid chromatography-high-resolution mass spectrometry (UPLC-HRMS) analysis confirmed that with l-serine and l-methionine the same product was formed as with SAM as donor (*m/z* = 285.19, Fig. 6B). To further confirm methyl group transfer from l-methionine to estrone, we used methyl-^13^C-l-methonine as the methyl donor and analyzed the product by UPLC-HRMS. The shift to *m/z* = 286.19 is indicative for the transfer of the ^13^C-methyl group to estradiol (Fig. 6B).

The possibility to follow methyl group transfer from l-methionine to estrone motivated us to use a similar setup for the identification of the anticipated methylcob(III)alamin intermediate. For this purpose, l-methionine and ATP were incubated with 25% (vol/vol) (NH_4_)_2_SO_4_ precipitated cell extracts in the absence of estrone. UPLC-HRMS analysis identified coeluting compounds with *m/z* = 672.8 and 664.79, indicative of methyl(III)cobalamin and its demethylated form, respectively, as evidenced by comparison with an authentic methyl(III)cobalamin standard (double positively charged ions, Fig. 6C). In assays with methyl-^13^C-l-methionine, the *m/z* peak at 664.79 was unaffected, whereas the methyl(III)cobalamin peak shifted by *m/z* = 0.5 to 673.3 as expected for double positively charged ^13^C-methyl(III)cobalamin ([M + 1+H]^2+^). In control experiments without ATP, virtually no ^13^C-methyl(III)cobalamin was detected. Taken together, these finding corroborate the methyl(III)cobalamin intermediate of estrogen methyltransferase during SAM-dependent estrogen methylation.

## DISCUSSION

This work identified SAM as the previously unknown stoichiometric methyl donor for the conversion of estrogens into androgens by a cobalamin-dependent methyltransferase in anaerobic bacteria. The combination of SAM and cobalamin cofactors has so far only been described for a few radical SAM enzymes involved in radical-based methyl transfer reactions (33). In these enzymes, one SAM abstracts a hydrogen atom from the substrate, and the radical formed then serves as methyl radical acceptor from methylcob(III)alamin. The latter is regenerated from the cob(II)alamin intermediate at the expense of a second SAM and an electron provided by an external donor. However, there is no evidence for the involvement of a radical SAM enzyme in anaerobic estradiol degradation. Instead, estradiol methylation is likely to occur via a nonradical mechanism with the deprotonated ring A phenolate serving as a suitable nucleophilic methyl cation acceptor (15). As genes encoding classical, nonradical SAM-dependent methyltransferases are absent in the estradiol-induced gene clusters, there remains little doubt that anaerobic estrogen degradation is initiated by an unorthodox, cobalamin-dependent SAM:estrogen methyltransferase, encoded by the estrogen-induced *emtABCD* genes.

**FIG 7:**
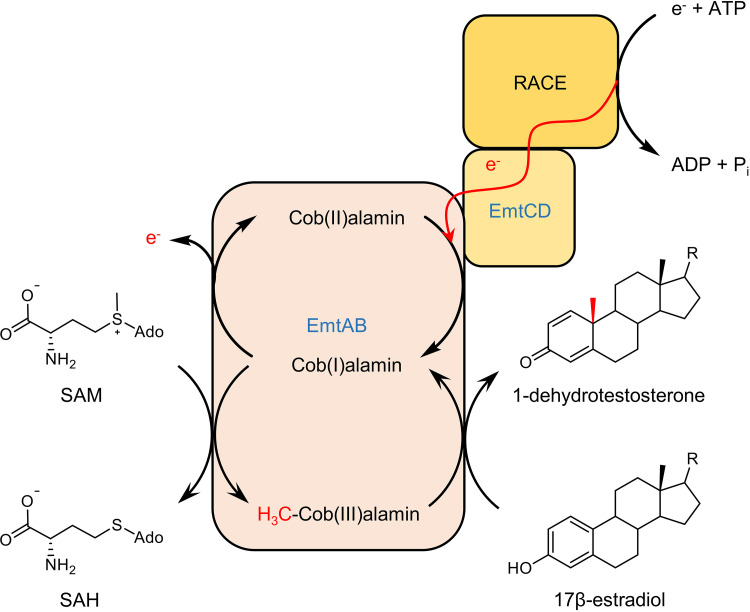
Proposed SAM-dependent conversion of estrogens into androgens by EMT. The EmtAB components transfer the methyl group from SAM to cob(I)alamin, and from the methylcob(III)alamin formed to estradiol. The exergonic first methyl transfer drives the endergonic second methyl transfer forward. The accidentally oxidized inactive cob(II)alamin is regenerated by electron transfer from an unknown electron donor probably mediated by ATP-dependent RACE, and the EmtCD components.

Methylation of carbon atoms usually depends on SAM as methyl donor either via radical or methyl cation transfer mechanisms (34, 35), but have never been reported for methylcobalamin- or methyl-THF-dependent methyltransferases. Our results suggest two partial reactions for the proposed SAM:estrogen methyltransferase system: (i) exergonic methyl transfer from SAM to cob(I)alamin, and (ii) endergonic methyl transfer from the methylcob(III)alamin intermediate to estradiol, with the first exergonic partial reaction pushing the second endergonic one forward (Fig. 7). A question rises; why there is no direct transfer from SAM to estradiol catalyzed by a conventional cobalamin-independent methyltransferase? It may be simply explained by the evolution of a methyl transfer system in which SAM- and cobalamin-dependent methyl transfer components of different origin have merged to EMT. However, one may also speculate that separation of SAM demethylation and estradiol methylation into two partial reactions may be beneficial for efficient estrogen methylation. E.g., unlike most of the known SAM-dependent methyltransferases, EMT is not inhibited by the product SAH, which appears to be important for unidirectional initiation of a catabolic pathway that provides the cell with carbon and energy. The use of a noncanonical SAM-and cobalamin-dependent methyltransferase may be important for preventing SAH inhibition.

Our results indicate that estradiol methylation strictly depends on both SAM and the artificial low-potential electron donor Ti(III)-citrate. The latter most likely keeps the EMT cofactor in the active cob(I)alamin state. NAD(P)H together with ATP, the assumed cosubstrates of a putative estradiol-induced RACE component, could not substitute for Ti(III)-citrate. Thus, the natural electron donor system for regenerating the active cob(I)alamin state needs to be studied in future work. The cofactor dependence identified in this work is in contrast to qualitative results reported in Wang et al. (15), where estradiol methylation essentially depended on ATP (22). This observed ATP dependence may be explained by l-methionine adenosyltransferase-catalyzed SAM synthesis from traces of SAM precursors present in the extracts used. The availability of higher biomass in the study presented here allowed for the conversion of much higher substrate amounts, which could be required for quantitative *in vitro* studies with protein fractions essentially free of interfering metabolites and side reactions.

It is obvious that standard cellular C1 metabolism, usually involved in biosynthetic and regulatory functions, can hardly cope with the stoichiometric provision of a C1 unit for estradiol catabolism (one methyl group per estradiol). To solve this problem, second copies of the genes involved in C1 metabolism from l-serine to SAM are present in the genomes of estrogen-degrading anaerobic bacteria. These genes are strongly upregulated during growth with estradiol to guarantee continuous stoichiometric SAM regeneration from catabolites formed during estradiol degradation via l-serine and l-methionine. Thus, oxygen-independent growth with estradiol does not only depend on a specific methyltransferase system and a B_12_-processing machinery, but also on an efficient channeling of C1 units to the initial methyl transfer reaction. In this light, it is important to note that the reported B_12_ dependence of estradiol-degrading anaerobes (15) has to be linked to two highly upregulated cobalamin-dependent enzymes: estradiol methyltransferase and l-methionine synthase involved in SAM regeneration. The optimized channeling of the cellular C1 pool toward SAM regeneration represents an attractive model for *in vivo* solutions for SAM-dependent methyl transfer reactions of biotechnological interest, e.g., in the synthesis of pharmaceuticals. There are currently considerable efforts to develop SAM regeneration systems for biocatalytic alkylation reactions (36, 37).

This work elucidated the cofactor requirement and regeneration of an unprecedented catabolic SAM- and cobalamin-dependent methyl transfer system involved in the microbial degradation of a globally important pollutant. The work also illustrates how the establishment of a catabolic pathway not only depends on the evolution/recruitment of proteins involved in uptake and catabolic and regulatory processes, but can also entail the redirection of existing anabolic reactions toward catabolic pathways for regeneration of a crucial cofactor.

## MATERIALS AND METHODS

### Chemicals and bacterial strains.

17β-estradiol, *S*-adenosyl-l-methionine (SAM), *S*-adenosyl-l-homocysteine (SAH), N^5^-methyltetrahydrofolate, tetrahydrofolate (THF), methylviologen, benzylviologen, methylcobalamin (MeCo), nicotinamidadenindinucleotide-phosphate (NADPH), l-homocysteine, l-serine, ATP (ATP), l-methionine, and methyl-^13^C-l-methionine were purchased from Carl Roth (Karlsruhe, Germany), Carbosynth (Compton Berkshire, UK), or Merck (Darmstadt, Germany). Other chemicals or reagents were of analytical or high-performance-liquid chromatographic (HPLC) grade. Denitratisoma oestradiolicum (DSM 16959) was obtained from the Deutsche Sammlung für Mikroorganismen und Zellkulturen (DSMZ) (Braunschweig, Germany). Escherichia Coli strain BL21(DE3) was purchased from New England BioLabs (Frankfurt am Main, Germany).

### Culture conditions and preparation of cell extracts.

*D. oestradiolicum* was cultivated under denitrifying conditions in bicarbonate-buffered medium (0.5 g liter^−1^ KH_2_PO_4_, 0.1 g liter^−1^ CaCl_2_, 0.5 g liter^−1^ NH_4_Cl, 0.8 mM MgSO_4_, and 0.1 mM CaCl_2_) at pH 6.9 supplemented with 1 mM 17β-estradiol, 5 mM NaNO_3_, 1 mM Na_2_SO_4_, vitamin-, trace element-, and selenium/tungsten solution at 28°C and harvested anaerobically in the exponential phase as previously described (14). Frozen cells were opened via a French pressure cell at 137 MPa, using two volumes of lysis buffer containing 50 mM 4-(2-hydroxyethyl)-1-piperazineethanesulfonic acid (HEPES)/KOH (pH 8.0) and 0.1 mg DNase I.

E. coli BL21(DE3) was cultivated in 2× YT medium (18 g liter^−1^ tryptone, 10 g liter^−1^ yeast extract, and 5 g liter^−1^ NaCl) supplemented with 100 μg ml^−1^ ampicillin. Induction of recombinant proteins was carried out in the early exponential phase of optical density at 578 nm (OD_578_) 0.4 to 0.8 with 200 ng ml^−1^ anhydrotetracycline for 20 h at 20°C; cells were harvested in the late exponential phase.

### Synthesis of 1-dehydrotestosterone.

DHT (1-dehydrotestosterone) was produced from commercially available testosterone. The enzyme assays employed (100 ml) contained 50 mM 3-(*N*-morpholino)propanesulfonic acid (MOPS)/KOH (pH 7.0), 10 mM K_3_(Fe[CN]_6_), 1 mM testosterone, 6% 2-hydroxypropyl-β-cyclodextrin (HPCD), and AcmB heterologously produced and enriched from E. coli BL21 (37). The product was extracted with twice the volume of ethyl acetate, evaporated (40°C, 2.4 × 10^7^ mPa), and purified by preparative high-performance liquid chromatography (HPLC) using a 2-propanol/H_2_O gradient. Samples were lyophilized and diluted in 2-propanol.

### Enzyme assays.

The conversion of estradiol/estrone to DHT/androsta-1,4-diene-3-one (ADD) was determined anaerobically in a glove box (95% N_2_, 5% H_2_, by vol) at 30°C in the dark as follows:

(i) The SAM-dependent conversion was measured in the presence of 50 mM HEPES/KOH (pH 8.0), 50 to 100 μM estradiol or estrone, 200 μM SAM, 1 mM MgCl_2_, 1.5 mM Ti(III)-citrate, and 10% (vol/vol) cell-free extract of *D. oestradiolicum* or 5% (vol/vol) of the 70% (NH_4_)_2_SO_4_-precipitated protein fraction. For determination of product and SAH stoichiometry, soluble proteins from *D. oestradiolicum* were subjected to (NH_4_)_2_SO_4_ precipitation. At 40% saturation, the activity remained in the supernatant, whereas at 70% the enzyme was found in the precipitate. The pellet was washed twice with 70% (NH_4_)_2_SO_4_-saturated 50 mM HEPES/KOH (pH 8.0) and diluted in 50 mM HEPES/KOH (pH 8.0) prior to use.
(ii) The conversion of estradiol or estrone with l-methionine as methyl donor was carried out in the presence of 50 mM HEPES/KOH (pH 8.0), 50 μM estradiol or estrone, 1 mM ATP, 1 mM MgCl_2_, 1.5 mM Ti(III)-citrate, 0.5 mM l-methionine and 10% (vol/vol) cell-free extract of *D. oestradiolicum*. For mass spectrometry-based detection of the methylcob(III)alamin intermediate, estrogens were omitted from the assays, and 25% (vol/vol) (NH_4_)_2_SO_4_-precipitated extracts of *D. oestradiolicum* were used in *in vitro* assays. Samples for mass spectrometry analysis were taken after 30 min incubation.
(iii) The conversion of estradiol/estrone with l-serine as methyl-donor was determined in the presence of 50 mM HEPES/KOH (pH 8.0), 50 μM estradiol/estrone, 1 mM ATP, 1 mM MgCl_2_, 1.5 mM Ti(III)-citrate, 0.5 mM NADH, 0.5 mM THF, 0.5 mM l-serine, 0.5 mM l-homocysteine and 10% (vol/vol) cell-free extract of *D. oestradiolicum*.

All enzyme assays were stopped by adding two volumes of 2-propanol (for analysis of steroid compounds) or 0.5% formic acid (analysis of SAH). After 2-fold centrifugation at 10,000 × *g*, the supernatant was applied to an Acquity H-class UPLC system (Waters) for analysis.

### UPLC analysis of steroid compounds.

An Acquity UPLC CSH C18 (1.7 μM, 2.1 × 100 mm, Waters) column was used (flow rate: 0.35 ml min^−1^; 40°C column temperature) to separate steroid compounds and methylcob(III)alamin using an acetonitrile gradient from 5% to 95% in 10 mM aqueous NH_4_OAc. For SAM and SAH analysis, an Acquity UPLC HSS T3 (1.8 μM, 2.1 × 100 mm, Waters) column was used (flowrate: 0.2 ml min^−1^; 30°C column temperature) applying an acetonitrile gradient from 0% to 20% in 0.1% (vol/vol) aqueous formic acid.

### Liquid chromatography-electrospray ionization-mass spectrometry analyses of steroid compounds.

Steroid metabolites were analyzed by an Acquity I-class UPLC system (Waters) using an Acquity UPLC CSH C18 (1.7 μm, 2.1 × 100 mm) column coupled to a Synapt G2-Si HRMS ESI-Q-TOF device (Waters). The acetonitrile gradient was as described above. Samples were measured in positive mode with a capillary voltage of 3 kV, 100°C source temperature, 450°C desolvation temperature, 750 liter min^−1^ N_2_ desolvation gas flow, and 30 liter min^−1^ N_2_ cone gas flow. LC-MS metabolites were evaluated using MassLynx (Waters). Metabolites were identified by their retention times, UV-vis spectra, by comparison with authentic standards, and *m/z* values.

### Proteome analysis.

*D. oestradiolicum* was grown in biological triplicates in 100-ml sealed bottles using 1 mM estradiol or 25 mM acetate as carbon source in the presence of 5 mM NaNO_3_ that was added continuously after consumption of NaNO_3_. Cells were harvested in the exponential growth phase (for estrogen-fed cells: OD_578nm_ 0.2 to 0.25; for acetate-fed cells: OD_578nm_ 0.25 to 0.3). The bacterial cell pellet was dissolved in 1 ml lysis buffer (8 M urea, 2 M thiourea, 1 mM phenylmethylsulfonyl fluoride, and disrupted by bead beating (FastPrep-24, MP Biomedicals, Santa Ana, CA, USA) (5.5 ms, 1 min, 3 cycles) followed by ultrasonication (UP50H, Hielscher, Germany) (cycle 0.5, amplitude 60%), and centrifugation (10,000 × *g*, 10 min). The protein lysate was loaded onto an SDS gel and run for 10 min. The gel pieces were cut, washed, and incubated with 25 mM 1,4-dithiothreitol (in 20 mM ammonium bicarbonate) for 1 h and 100 mM iodoacetamide (in 20 mM ammonium bicarbonate) for 30 min. Afterward it was destained, dehydrated, and proteolytically cleaved overnight at 37°C with trypsin (Promega). Peptides were extracted and desalted using ZipTip-μC18 tips (Merck Millipore, Darmstadt, Germany) and resuspended in 0.1% formic acid. Peptide lysates were injected into nano HPLC (UltiMate 3000 RSLCnano, Dionex, Thermo Fisher Scientific, Waltham, MA, USA). Peptide separation was performed on a C_18_ reverse-phase trapping column (C_18_ PepMap100, 300 μm × 5 mm, particle size 5 μm) (nanoViper, Thermo Fisher Scientific, Waltham, MA, USA), followed by a C_18_-reverse-phase analytical column (Acclaim PepMap 100, 75 μm x 25 cm, particle size 3 μm) (nanoViper, Thermo Fisher Scientific). Mass spectrometric analysis of peptides was performed on a Q Exactive HF mass spectrometer (Thermo Fisher Scientific, Waltham, MA, USA) coupled with a TriVersa NanoMate (Advion, Ltd., Harlow, UK) source in LC chip coupling mode. LC gradient, ionization mode, and mass spectrometry mode have been used as described (38).

MS data were analyzed by the Proteome Discoverer software (v.2.3, Thermo Fisher Scientific, Waltham, MA, USA) using SEQUEST HT as search engine against the protein-coding sequences of *D. oestradiolicum*. Search settings were set to trypsin (Full); maximum missed cleavage: 2; precursor mass tolerance: 10 ppm; fragment mass tolerance: 0.02 Da; two missed cleavage sites; dynamic modifications oxidation (Met); static modifications carbamidomethylation (Cys). Only peptides that passed the FDR thresholds set in the Percolator node of <1% FDR q value, and were rank 1 peptides, were considered for protein identification.

### Genome sequencing and assembly.

Genomic DNA of *D. oestradiolicum* was extracted using the GenElute bacterial genomic DNA kit (Sigma-Aldrich), and sequencing was carried out by combining short read (Illumina) and long read (Oxford Nanopore) technologies. For nanopore sequencing, library preparation was performed starting with 1 μg of DNA, following the 1D Native barcoding genomic DNA (with EXP-NBD103 and SQK-LSK109) protocol (Oxford Nanopore). The library was sequenced using a nanopore R9.4.1 flow cell (Oxford Nanopore) and the MinION device, with the MinKNOW v.1.14.1 and Albacore v.2.3.1 softwares. For Illumina sequencing, 250 ng of DNA was sonicated to a 100 to 1,000 bp size range using the E220 Covaris Focused-ultrasonicator instrument (Covaris, Inc.). The fragments were end-repaired, 3′-adenylated, and NEXTflex HT Barcodes (Bioo Scientific Corporation) were added using the NEBNext DNA modules products (New England BioLabs). After two consecutive clean-ups with 1×AMPure XP, the ligated product was amplified by 12 PCR cycles using the Kapa Hifi Hotstart NGS library amplification kit (Kapa Biosystems), followed by purification with 0.6×AMPure XP. After library-profile analysis conducted with an Agilent 2100 Bioanalyzer (Agilent Technologies) and qPCR quantification (MxPro, Agilent Technologies), the library was sequenced on an Illumina HiSeq 2500 platform (2 × 250 bp; Illumina, Inc.). Reads were trimmed, and low-quality nucleotides (Q < 20), sequencing adaptors, and primer sequences were discarded, as well as reads shorter than 30 nucleotides after trimming.

The long reads generated with the nanopore technology were first corrected using the canu software error correction module (39) and half of the Illumina reads. The corrected nanopore reads were then assembled using the smart *de novo* engine (Jue Ruan, unpublished, https://github.com/ruanjue/smartdenovo, yielding a single 4.17-Mbp unitig corresponding to the main chromosome, along with the sequence of a 124-Kbp plasmid. Both sequences were subjected to three rounds of error correction (polishing) using the pilon software (40). Both the main chromosome and the plasmid sequences were uploaded to the Microscope platform (41) for annotation, comparative genomic analyses, and human interaction.

### Data availability.

Genome sequences are available from the ENA/NCBI/DDBJ databases under accession numbers LR778301 and LR778302, respectively (study identifier PRJEB37013).
