# Supplementary figures and images for: Channeling C1 Metabolism toward S-Adenosylmethionine-Dependent Conversion of Estrogens to Androgens in Estrogen-Degrading Bacteria
Source: mBio. 2020 Aug 25;11(4):e01259-20. doi: 10.1128/mBio.01259-20 (PMC7448270; doi:10.1128/mBio.01259-20)

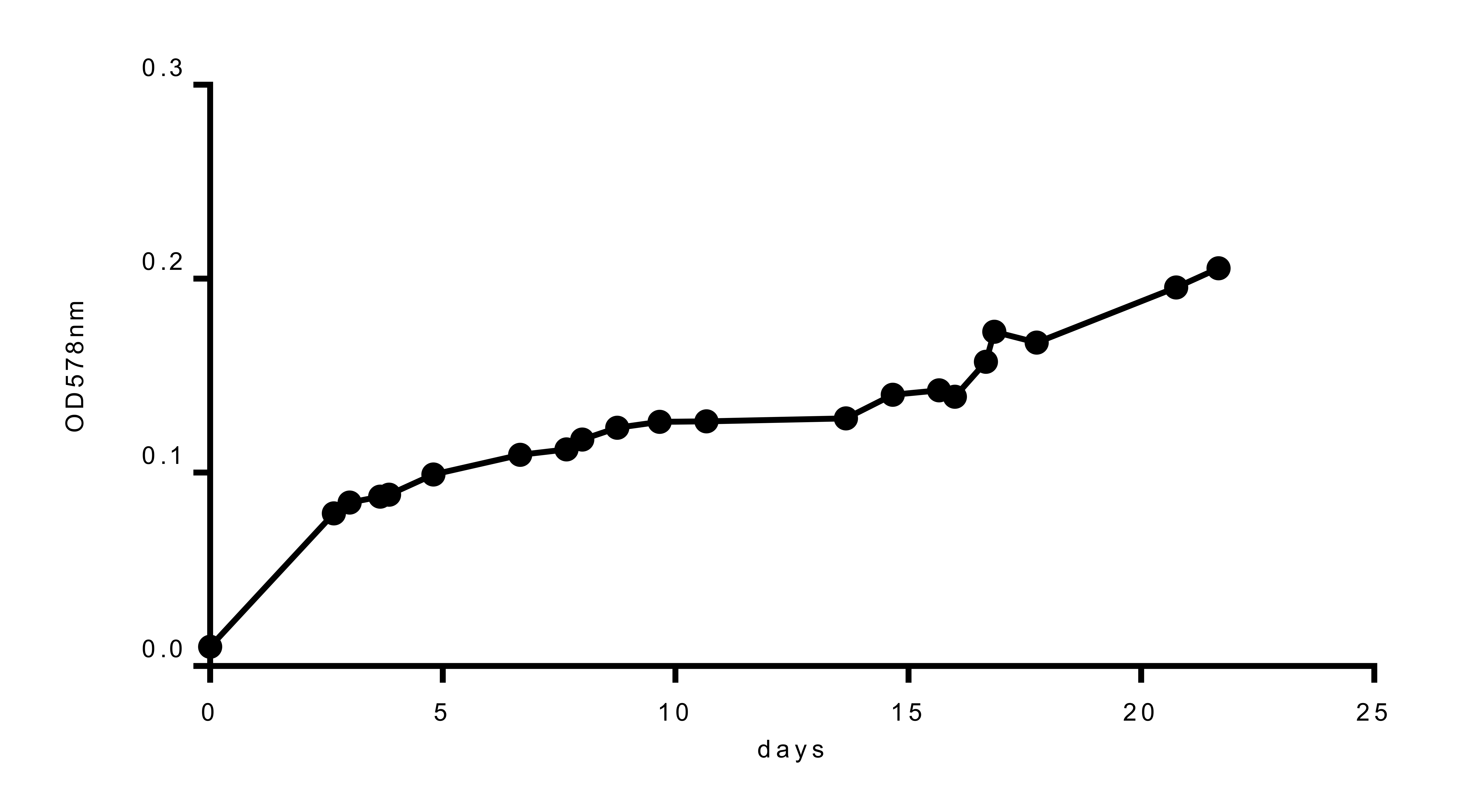

Supplement: FIG S1 [file mBio.01259-20-sf001.tif]

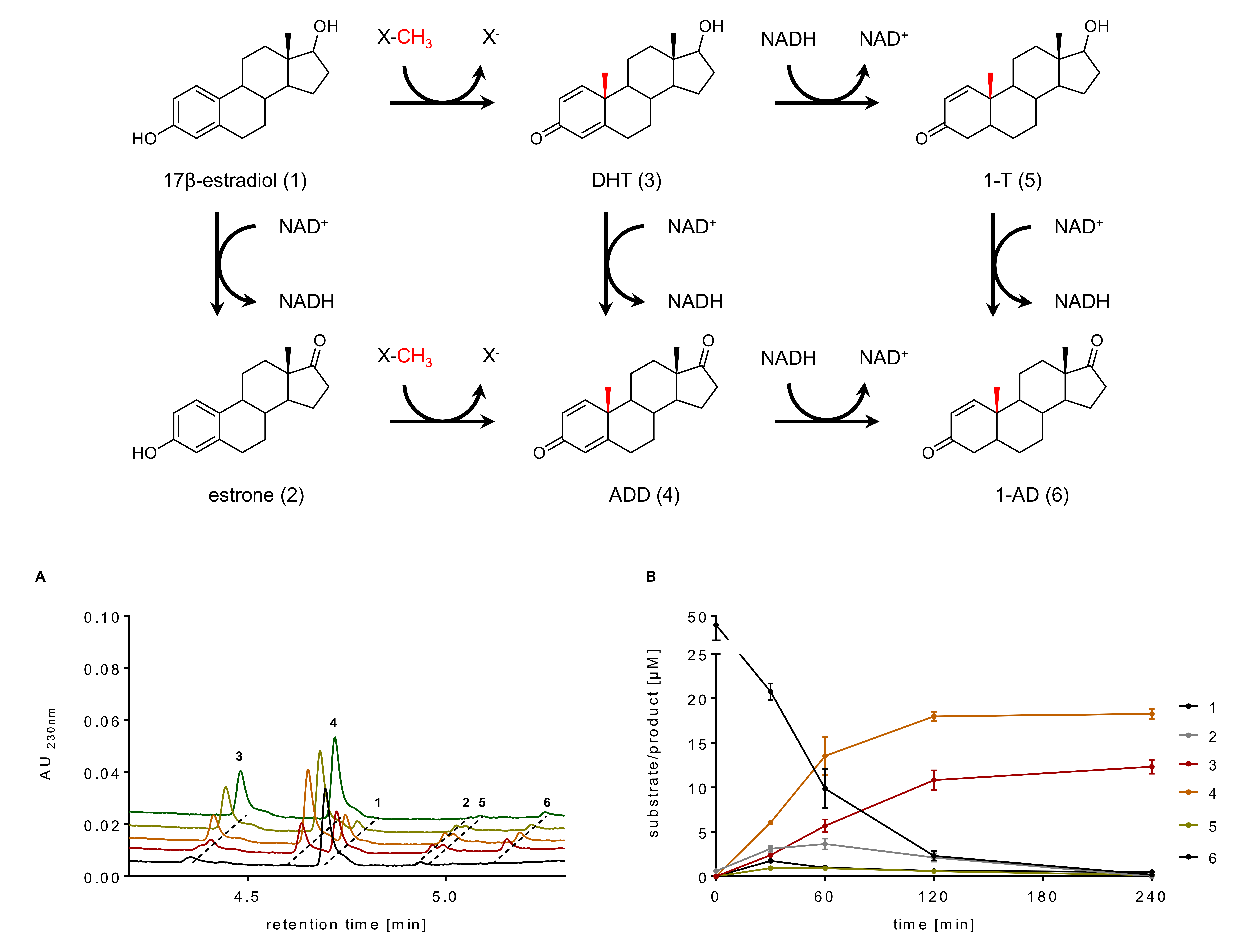

Supplement: FIG S2 [file mBio.01259-20-sf002.tif]

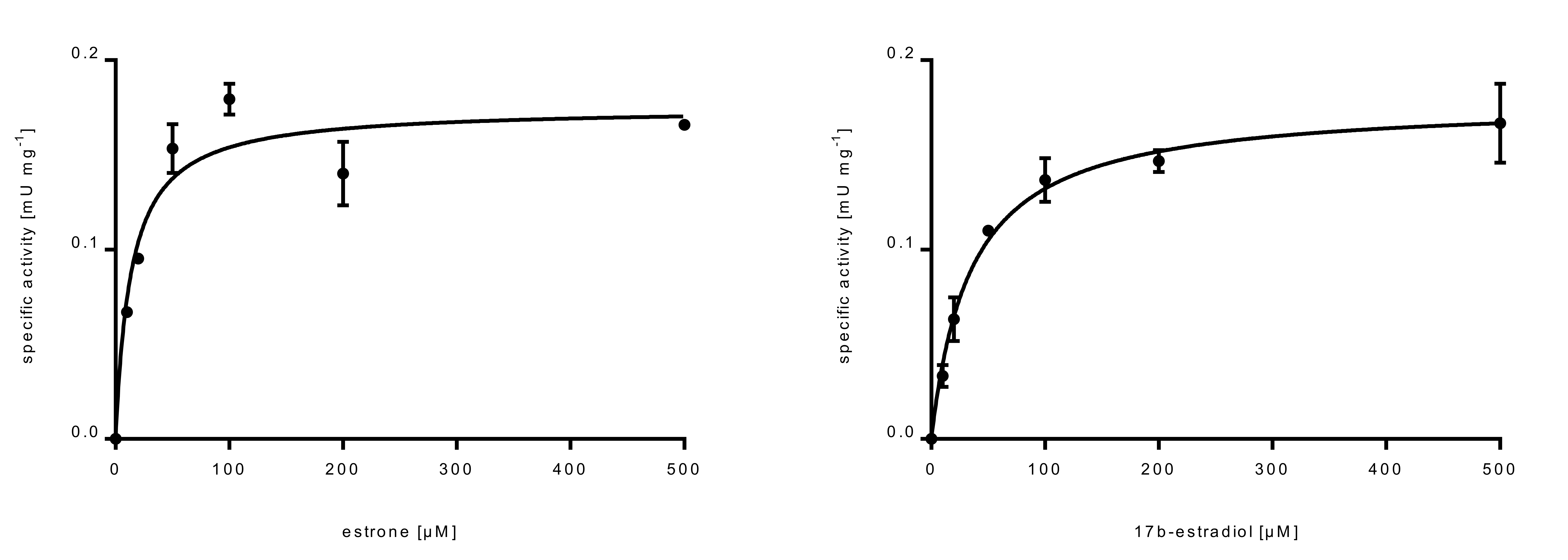

Supplement: FIG S3 [file mBio.01259-20-sf003.tif]

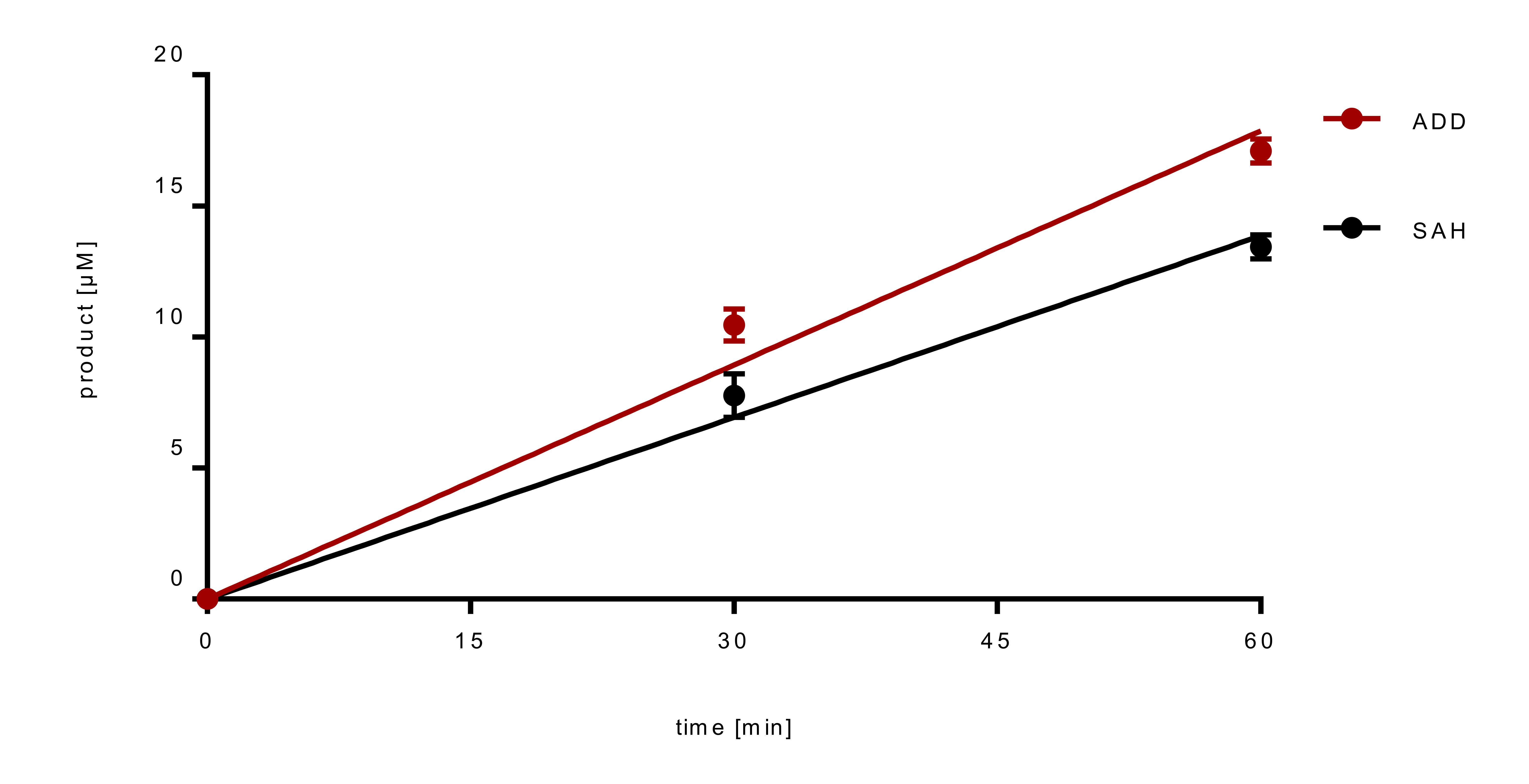

Supplement: FIG S4 [file mBio.01259-20-sf004.tif]

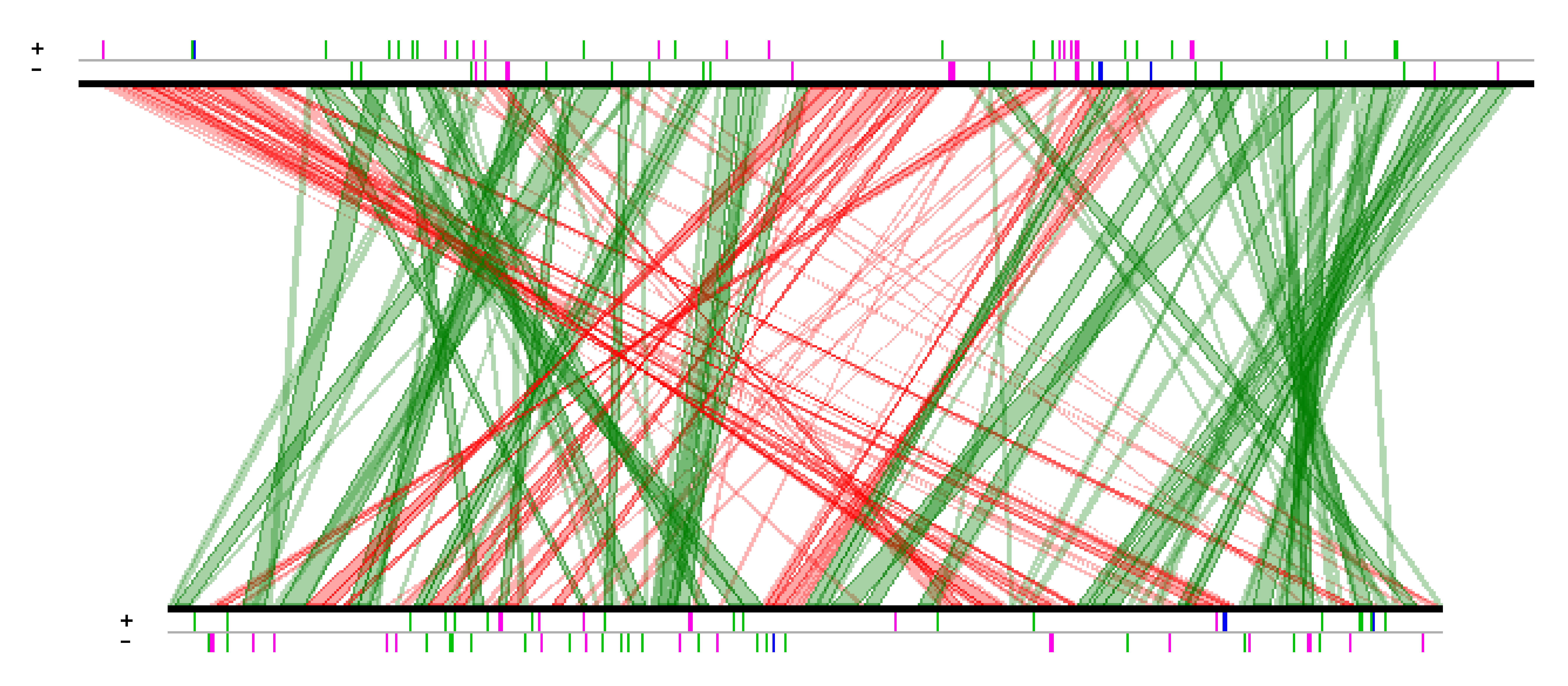

Supplement: FIG S5 [file mBio.01259-20-sf005.tif]

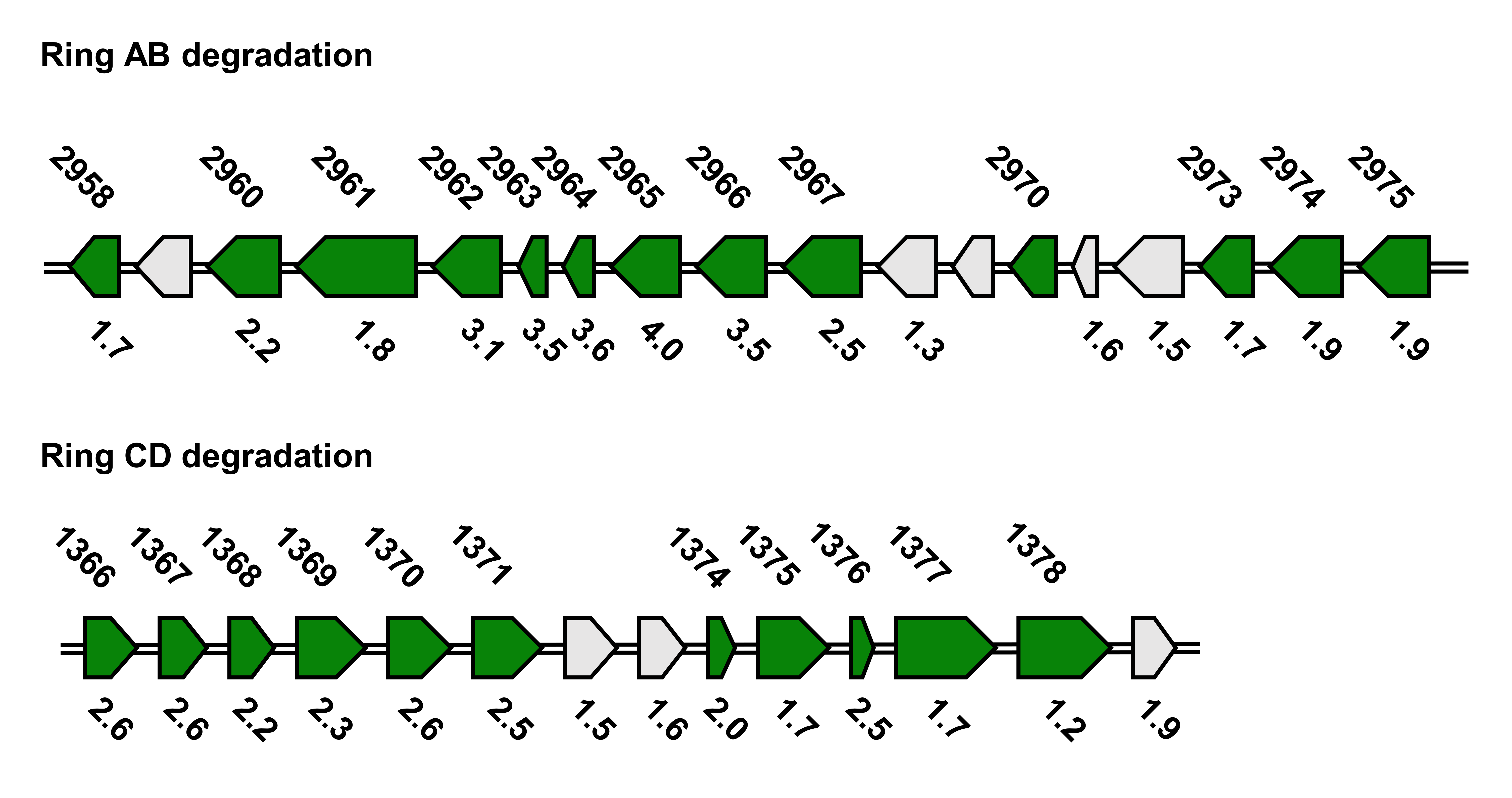

Supplement: FIG S6 [file mBio.01259-20-sf006.tif]
