# Supplementary material for: Channeling C1 Metabolism toward S-Adenosylmethionine-Dependent Conversion of Estrogens to Androgens in Estrogen-Degrading Bacteria
Source: mBio. 2020 Aug 25;11(4):e01259-20. doi: 10.1128/mBio.01259-20 (PMC7448270; doi:10.1128/mBio.01259-20)
Supplement: TABLE S1 [file mBio.01259-20-st001.docx]

**Table S1.** Genome-level synteny statistics for *D. oestradiolicum* (top 20 closest genomes). Putative orthologous relations between two genomes were defined as gene pairs satisfying the bi-directional best hit (BBH) criterion, or a blastP alignment threshold, a minimum of 35% sequence identity on 80% of the length of the smallest protein. These relations were subsequently used to search for conserved gene clusters, e.g. synteny groups (syntons) among several bacterial genomes. All possible kinds of chromosomal rearrangements were allowed (inversion, insertion/deletion). A gap parameter, representing the maximum number of consecutive genes, which were not involved in a synteny group, was set to five genes.

| Nb | % | Nb | % | Synton Nb | Min | Avg | Max | Replicon Name |
| --- | --- | --- | --- | --- | --- | --- | --- | --- |
| 3960 | 98.90 | 3881 | 96.93 | 1 | 3960 | 3960.00 | 3960 | *Denitratisoma oestradiolicum* DSM16959 unknown DSM_v1_u DSMu |
| 2646 | 66.08 | 2548 | 63.64 | 441 | 1 | 6.84 | 59 | *Denitratisoma* sp. DHT3 DHT3 chromosome DHT3_v1_ DHT3 |
| 1705 | 42.58 | 2071 | 51.72 | 473 | 1 | 4.04 | 40 | *Azoarcus tolulyticus* ATCC 51758 WGS FTMD01_ FTMD01 |
| 1704 | 42.56 | 2036 | 50.85 | 393 | 1 | 4.71 | 51 | *Dechloromonas aromatica* RCB chromosome Daro_;Daro_R CP000089 |
| 1695 | 42.33 | 1982 | 49.50 | 443 | 1 | 4.14 | 29 | *Thauera terpenica* 58Eu WGS TTPv1_ TTP |
| 1671 | 41.73 | 2100 | 52.45 | 650 | 1 | 3.62 | 57 | *Thauera selenatis* AX ATCC 55363 WGS CACRv1_ NZ_CACR |
| 1637 | 40.88 | 1955 | 48.83 | 434 | 1 | 4.14 | 47 | *Thauera* sp. nov. Piv1 chromosome C4PIVTH_v1_ C4PIVTH |
| 1622 | 40.51 | 1968 | 49.15 | 454 | 1 | 3.91 | 29 | Thauera sp. 27 WGS AMXBv1_ NZ_AMXB |
| 1621 | 40.48 | 1824 | 45.55 | 417 | 1 | 4.23 | 41 | *Sterolibacterium denitrificans* Chol chromosome SDENCHOL_v1_ SDENCHOL |
| 1588 | 39.66 | 1943 | 48.53 | 442 | 1 | 3.92 | 46 | *Thauera* sp. 28 WGS AMXAv1_ NZ_AMXA |
| 1573 | 39.29 | 1911 | 47.73 | 407 | 1 | 4.15 | 47 | *Thauera* sp. 63 WGS AMXCv1_ NZ_AMXC |
| 1563 | 39.04 | 1900 | 47.45 | 396 | 1 | 4.23 | 52 | *Thauera* sp. MZ1T chromosome Tmz1t_ NC_011662 |
| 1548 | 38.66 | 1876 | 46.85 | 417 | 1 | 4.02 | 43 | *Aromatoleum aromaticum* EbN1 chromosome c1A CR555306 |
| 1506 | 37.61 | 1810 | 45.20 | 403 | 1 | 4.09 | 45 | *Thauera chlorobenzoica* 3CB1 WGS TCB_v1_ TCB |
| 1489 | 37.19 | 1868 | 46.65 | 369 | 1 | 4.24 | 44 | Candidatus *Accumulibacter phosphatis* clade IIA UW-1 chromosome CAP2UW1_ NC_013194 |
| 1484 | 37.06 | 1865 | 46.58 | 428 | 1 | 3.79 | 46 | *Thauera aminoaromatica* S2 WGS TAAv1_ TAA |
| 1481 | 36.99 | 1930 | 48.20 | 480 | 1 | 3.44 | 46 | *Thauera phenylacetica* B4P WGS AMXFv1_ NZ_AMXF |
| 1461 | 36.49 | 1792 | 44.76 | 342 | 1 | 4.48 | 41 | *Methyloversatilis universalis* FAM500 WGS MFAM500v1_ MFAM500 |
| 1454 | 36.31 | 1787 | 44.63 | 338 | 1 | 4.50 | 41 | *Methyloversatilis universalis* FAM5 WGS METUNv2_ AFHG |
| 1447 | 36.14 | 1834 | 45.80 | 402 | 1 | 3.88 | 32 | *Thauera linaloolentis* 47Lol = DSM 12138 WGS AMXEv1_ NZ_AMXE |
