# Supplementary material for: Channeling C1 Metabolism toward S-Adenosylmethionine-Dependent Conversion of Estrogens to Androgens in Estrogen-Degrading Bacteria
Source: mBio. 2020 Aug 25;11(4):e01259-20. doi: 10.1128/mBio.01259-20 (PMC7448270; doi:10.1128/mBio.01259-20)
Supplement: TABLE S2 [file mBio.01259-20-st002.docx]

**Table S2.** Original data set of differential proteome analyses: abundances of gene products, log2 ratios and p-values for proteins are listed and compared in cells grown with 17β-estradiol/nitrate vs acetate/nitrate.

| Accession | Description | log2/fc | p-value |
| --- | --- | --- | --- |
| DENOEST_v1_p0150 | ID:63767093 Conjugative relaxase domain-containing protein [Denitratisoma oestradiolicum Fahrbach xx] | 1.335772 | 0.001391 |
| DENOEST_v1_p0148 | ID:63767091 conserved protein of unknown function [Denitratisoma oestradiolicum Fahrbach xx] | 0.97492 | 0.004026 |
| DENOEST_v1_p0147 | ID:63767090 conserved protein of unknown function [Denitratisoma oestradiolicum Fahrbach xx] | 0.427376 | 0.008351 |
| DENOEST_v1_p0146 | ID:63767089 conserved membrane protein of unknown function [Denitratisoma oestradiolicum Fahrbach xx] | 1.152001 | 0.002676 |
| DENOEST_v1_p0139 | ID:63767082 Conjugal transfer protein TraE [Denitratisoma oestradiolicum Fahrbach xx] | -0.6225 | 0.018264 |
| DENOEST_v1_p0138 | ID:63767081 Conjugal transfer protein TraK [Denitratisoma oestradiolicum Fahrbach xx] | -1.40119 | 0.038696 |
| DENOEST_v1_p0134 | ID:63767077 protein of unknown function [Denitratisoma oestradiolicum Fahrbach xx] | -19.9368 | 0.003619 |
| DENOEST_v1_p0133 | ID:63767076 Type-IV secretion system protein TraC (fragment) [Denitratisoma oestradiolicum Fahrbach xx] | -22.6406 | 2.07E-07 |
| DENOEST_v1_p0132 | ID:63767075 conserved protein of unknown function [Denitratisoma oestradiolicum Fahrbach xx] | -3.09918 | 0.006617 |
| DENOEST_v1_p0131 | ID:63767074 Peptidase S26 [Denitratisoma oestradiolicum Fahrbach xx] | -1.72855 | 0.024941 |
| DENOEST_v1_p0128 | ID:63767071 Type-F conjugative transfer system pilin assembly protein TrbC [Denitratisoma oestradiolicum Fahrbach xx] | -0.6148 | 0.011016 |
| DENOEST_v1_p0124 | ID:63767067 Conjugal transfer protein TraH [Denitratisoma oestradiolicum Fahrbach xx] | -1.60405 | 0.000164 |
| DENOEST_v1_p0123 | ID:63767066 TraG domain protein [Denitratisoma oestradiolicum Fahrbach xx] | -1.39097 | 0.004309 |
| DENOEST_v1_p0114 | ID:63767057 conserved protein of unknown function [Denitratisoma oestradiolicum Fahrbach xx] | -0.84147 | 0.014735 |
| DENOEST_v1_p0096 | ID:63767039 Cobyrinic acid a,c-diamide synthase [Denitratisoma oestradiolicum Fahrbach xx] | 0.646352 | 0.041809 |
| DENOEST_v1_p0095 | ID:63767038 ParB-like partition protein [Denitratisoma oestradiolicum Fahrbach xx] | 0.659932 | 0.013346 |
| DENOEST_v1_p0093 | ID:63767036 conserved protein of unknown function [Denitratisoma oestradiolicum Fahrbach xx] | 0.884157 | 0.005558 |
| DENOEST_v1_p0089 | ID:63767032 Replication initiator protein A [Denitratisoma oestradiolicum Fahrbach xx] | 0.981921 | 0.004055 |
| DENOEST_v1_p0087 | ID:63767030 conserved protein of unknown function [Denitratisoma oestradiolicum Fahrbach xx] | 1.194673 | 1.67E-05 |
| DENOEST_v1_p0081 | ID:63767024 Single-stranded DNA-binding protein [Denitratisoma oestradiolicum Fahrbach xx] | -1.76233 | 0.002508 |
| DENOEST_v1_p0076 | ID:63767019 Methyltransferase type 11 [Denitratisoma oestradiolicum Fahrbach xx] | -6.50373 | 0.007522 |
| DENOEST_v1_p0063 | ID:63767006 conserved protein of unknown function [Denitratisoma oestradiolicum Fahrbach xx] | 0.37778 | 0.036968 |
| DENOEST_v1_p0053 | ID:63766996 DNA-binding response regulator (modular protein) [Denitratisoma oestradiolicum Fahrbach xx] | 0.672563 | 0.02755 |
| DENOEST_v1_p0051 | ID:63766994 conserved exported protein of unknown function [Denitratisoma oestradiolicum Fahrbach xx] | -1.05503 | 0.002231 |
| DENOEST_v1_p0042 | ID:63766985 Thiol:disulfide interchange protein [Denitratisoma oestradiolicum Fahrbach xx] | -1.16874 | 0.000155 |
| DENOEST_v1_p0040 | ID:63766983 Type-IV secretion system protein TraC (fragment) [Denitratisoma oestradiolicum Fahrbach xx] | -21.2461 | 0.000112 |
| DENOEST_v1_p0036 | ID:63766979 Conjugal transfer pilus assembly protein TraW [Denitratisoma oestradiolicum Fahrbach xx] | -0.97819 | 0.00421 |
| DENOEST_v1_p0028 | ID:63766971 TraF-like protein [Denitratisoma oestradiolicum Fahrbach xx] | -2.38247 | 0.000282 |
| DENOEST_v1_p0008 | ID:63766951 conserved protein of unknown function [Denitratisoma oestradiolicum Fahrbach xx] | 0.977456 | 0.000419 |
| DENOEST_v1_3948 | ID:63771172 conserved protein of unknown function [Denitratisoma oestradiolicum Fahrbach xx] | -2.14405 | 0.001447 |
| DENOEST_v1_3944 | ID:63771168 membrane protein of unknown function [Denitratisoma oestradiolicum Fahrbach xx] | 2.374571 | 0.000558 |
| DENOEST_v1_3943 | ID:63771167 conserved protein of unknown function [Denitratisoma oestradiolicum Fahrbach xx] | 15.50141 | 0.016611 |
| DENOEST_v1_3942 | ID:63771166 conserved protein of unknown function [Denitratisoma oestradiolicum Fahrbach xx] | 2.290346 | 0.00174 |
| DENOEST_v1_3941 | ID:63771165 conserved exported protein of unknown function [Denitratisoma oestradiolicum Fahrbach xx] | 0.401533 | 0.011189 |
| DENOEST_v1_3935 | ID:63771159 conserved protein of unknown function [Denitratisoma oestradiolicum Fahrbach xx] | -0.68173 | 0.032559 |
| DENOEST_v1_3934 | ID:63771158 conserved protein of unknown function [Denitratisoma oestradiolicum Fahrbach xx] | -0.79217 | 0.005909 |
| DENOEST_v1_3933 | ID:63771157 ABC transporter ATP-binding protein [Denitratisoma oestradiolicum Fahrbach xx] | -0.63692 | 0.002216 |
| DENOEST_v1_3931 | ID:63771155 gmd\| GDP-mannose 4,6-dehydratase [Denitratisoma oestradiolicum Fahrbach xx] | -0.82693 | 0.000344 |
| DENOEST_v1_3930 | ID:63771154 rmd\| GDP-6-deoxy-D-mannose reductase [Denitratisoma oestradiolicum Fahrbach xx] | -0.75422 | 0.043347 |
| DENOEST_v1_3927 | ID:63771151 conserved protein of unknown function [Denitratisoma oestradiolicum Fahrbach xx] | -1.07578 | 0.019401 |
| DENOEST_v1_3925 | ID:63771149 xanB\| Mannose-6-phosphate isomerase / Mannose-1-phosphate guanylyl transferase [Denitratisoma oestradiolicum Fahrbach xx] | -1.02298 | 0.001512 |
| DENOEST_v1_3924 | ID:63771148 Channel protein TolC [Denitratisoma oestradiolicum Fahrbach xx] | -0.54405 | 0.008977 |
| DENOEST_v1_3923 | ID:63771147 Type I secretion membrane fusion protein, HlyD family [Denitratisoma oestradiolicum Fahrbach xx] | -0.62618 | 0.009231 |
| DENOEST_v1_3921 | ID:63771145 Glutathione gamma-glutamylcysteinyltransferase [Denitratisoma oestradiolicum Fahrbach xx] | 0.788633 | 0.002013 |
| DENOEST_v1_3920 | ID:63771144 conserved protein of unknown function [Denitratisoma oestradiolicum Fahrbach xx] | 0.800351 | 0.000274 |
| DENOEST_v1_3919 | ID:63771143 conserved protein of unknown function [Denitratisoma oestradiolicum Fahrbach xx] | 1.991957 | 0.000436 |
| DENOEST_v1_3918 | ID:63771142 conserved protein of unknown function [Denitratisoma oestradiolicum Fahrbach xx] | 0.554573 | 0.010537 |
| DENOEST_v1_3917 | ID:63771141 nadA\| Quinolinate synthase A [Denitratisoma oestradiolicum Fahrbach xx] | -0.3911 | 0.003231 |
| DENOEST_v1_3915 | ID:63771139 Cation diffusion facilitator family transporter [Denitratisoma oestradiolicum Fahrbach xx] | 1.077392 | 0.001808 |
| DENOEST_v1_3914 | ID:63771138 glnA\| glutamine synthetase [Denitratisoma oestradiolicum Fahrbach xx] | -0.59723 | 2.06E-05 |
| DENOEST_v1_3909 | ID:63771133 birA\| putative biotin--acetyl-CoA-carboxylase ligase [Denitratisoma oestradiolicum Fahrbach xx] | -0.47413 | 0.012776 |
| DENOEST_v1_3907 | ID:63771131 trpB\| Tryptophan synthase beta chain 2 [Denitratisoma oestradiolicum Fahrbach xx] | -0.18058 | 0.0227 |
| DENOEST_v1_3902 | ID:63771126 putative TonB-dependent receptor [Denitratisoma oestradiolicum Fahrbach xx] | 22.62262 | 0.000171 |
| DENOEST_v1_3894 | ID:63771118 Acid--CoA ligase [Denitratisoma oestradiolicum Fahrbach xx] | 3.090551 | 0.002543 |
| DENOEST_v1_3891 | ID:63771115 RND family efflux transporter, MFP subunit [Denitratisoma oestradiolicum Fahrbach xx] | -0.57028 | 0.000479 |
| DENOEST_v1_3889 | ID:63771113 RND transporter [Denitratisoma oestradiolicum Fahrbach xx] | -0.68805 | 0.018945 |
| DENOEST_v1_3888 | ID:63771112 mdtB\| multidrug efflux system, subunit B [Denitratisoma oestradiolicum Fahrbach xx] | 2.455111 | 0.020513 |
| DENOEST_v1_3885 | ID:63771109 1-(5-phosphoribosyl)-5-((5-phosphoribosylamino)methylideneamino)imidazole-4-carboxamide isomerase [Denitratisoma oestradiolicum Fahrbach xx] | -0.40204 | 0.007905 |
| DENOEST_v1_3884 | ID:63771108 ubiE\| bifunctional 2-octaprenyl-6-methoxy-1,4-benzoquinone methylase and S-adenosylmethionine:2-DMK methyltransferase [Denitratisoma oestradiolicum Fahrbach xx] | -0.43682 | 0.008706 |
| DENOEST_v1_3881 | ID:63771105 nemA\| N-ethylmaleimide reductase [Denitratisoma oestradiolicum Fahrbach xx] | -0.82697 | 0.010919 |
| DENOEST_v1_3872 | ID:63771096 Diguanylate cyclase (GGDEF) domain-containing protein [Denitratisoma oestradiolicum Fahrbach xx] | -0.32141 | 0.00187 |
| DENOEST_v1_3860 | ID:63771084 conserved protein of unknown function [Denitratisoma oestradiolicum Fahrbach xx] | -2.41374 | 0.001523 |
| DENOEST_v1_3859 | ID:63771083 Methyltransferase [Denitratisoma oestradiolicum Fahrbach xx] | -22.4534 | 0.000359 |
| DENOEST_v1_3837 | ID:63771061 atpC\| F1 sector of membrane-bound ATP synthase, epsilon subunit [Denitratisoma oestradiolicum Fahrbach xx] | -1.69817 | 0.002415 |
| DENOEST_v1_3836 | ID:63771060 atpD\| membrane-bound ATP synthase , F1 sector, beta-subunit [Denitratisoma oestradiolicum Fahrbach xx] | -1.69372 | 0.000164 |
| DENOEST_v1_3835 | ID:63771059 atpG\| F1 sector of membrane-bound ATP synthase, gamma subunit [Denitratisoma oestradiolicum Fahrbach xx] | -1.68703 | 0.000243 |
| DENOEST_v1_3834 | ID:63771058 atpA\| membrane-bound ATP synthase , F1 sector, alpha-subunit [Denitratisoma oestradiolicum Fahrbach xx] | -1.62894 | 2.74E-05 |
| DENOEST_v1_3833 | ID:63771057 atpH\| ATP synthase subunit delta [Denitratisoma oestradiolicum Fahrbach xx] | -1.72336 | 0.002115 |
| DENOEST_v1_3832 | ID:63771056 atpF\| F0 sector of membrane-bound ATP synthase, subunit b [Denitratisoma oestradiolicum Fahrbach xx] | -1.66123 | 0.004151 |
| DENOEST_v1_3828 | ID:63771052 parB\| putative chromosome-partitioning protein ParB [Denitratisoma oestradiolicum Fahrbach xx] | -0.78166 | 0.000629 |
| DENOEST_v1_3827 | ID:63771051 conserved protein of unknown function [Denitratisoma oestradiolicum Fahrbach xx] | 0.499943 | 0.012267 |
| DENOEST_v1_3825 | ID:63771049 gidA\| glucose-inhibited cell-division protein [Denitratisoma oestradiolicum Fahrbach xx] | -0.47448 | 0.002438 |
| DENOEST_v1_3824 | ID:63771048 trmE\| GTPase [Denitratisoma oestradiolicum Fahrbach xx] | -1.20279 | 8.81E-05 |
| DENOEST_v1_3819 | ID:63771043 dnaN\| DNA polymerase III, beta subunit [Denitratisoma oestradiolicum Fahrbach xx] | -0.18716 | 0.027833 |
| DENOEST_v1_3818 | ID:63771042 gyrB\| DNA gyrase, subunit B [Denitratisoma oestradiolicum Fahrbach xx] | -0.57809 | 0.00296 |
| DENOEST_v1_3812 | ID:63771036 Monooxygenase [Denitratisoma oestradiolicum Fahrbach xx] | -1.0931 | 0.027575 |
| DENOEST_v1_3805 | ID:63771029 sufS\| selenocysteine lyase, PLP-dependent [Denitratisoma oestradiolicum Fahrbach xx] | -4.22706 | 7.68E-05 |
| DENOEST_v1_3803 | ID:63771027 sufC\| component of SufBCD complex, ATP-binding component of ABC superfamily [Denitratisoma oestradiolicum Fahrbach xx] | -2.41346 | 0.000906 |
| DENOEST_v1_3799 | ID:63771023 ribB\| 3,4-dihydroxy-2-butanone 4-phosphate synthase [Denitratisoma oestradiolicum Fahrbach xx] | -0.20195 | 0.046334 |
| DENOEST_v1_3797 | ID:63771021 transcription antitermination protein (modular protein) [Denitratisoma oestradiolicum Fahrbach xx] | -0.5474 | 0.020553 |
| DENOEST_v1_3792 | ID:63771016 recA\| DNA strand exchange and recombination protein with protease and nuclease activity. [Denitratisoma oestradiolicum Fahrbach xx] | -0.93843 | 0.000594 |
| DENOEST_v1_3790 | ID:63771014 lptB\| putative lipopolysaccharide transport protein B: ATP-binding component of ABC superfamily [Denitratisoma oestradiolicum Fahrbach xx] | -1.37485 | 8.83E-05 |
| DENOEST_v1_3789 | ID:63771013 rpoN\| RNA polymerase, sigma 54 (sigma N) factor [Denitratisoma oestradiolicum Fahrbach xx] | -0.57676 | 0.004087 |
| DENOEST_v1_3787 | ID:63771011 ptsN\| Phosphotransferase enzyme IIA component [Denitratisoma oestradiolicum Fahrbach xx] | -0.88169 | 0.004685 |
| DENOEST_v1_3786 | ID:63771010 hprK\| HPr kinase/phosphorylase [Denitratisoma oestradiolicum Fahrbach xx] | -0.70692 | 0.005076 |
| DENOEST_v1_3782 | ID:63771006 Serine-pyruvate transaminase [Denitratisoma oestradiolicum Fahrbach xx] | -0.60527 | 0.004355 |
| DENOEST_v1_3781 | ID:63771005 ydaO\| putative C32 tRNA thiolase [Denitratisoma oestradiolicum Fahrbach xx] | -1.32506 | 0.002009 |
| DENOEST_v1_3774 | ID:63770998 phaJ\| (R)-specific enoyl-CoA hydratase [Denitratisoma oestradiolicum Fahrbach xx] | -0.53029 | 0.006205 |
| DENOEST_v1_3769 | ID:63770993 conserved protein of unknown function [Denitratisoma oestradiolicum Fahrbach xx] | -16.3105 | 0.000128 |
| DENOEST_v1_3768 | ID:63770992 2,5-dichloro-2,5-cyclohexadiene-1,4-diol dehydrogenase [Denitratisoma oestradiolicum Fahrbach xx] | -0.83454 | 0.000392 |
| DENOEST_v1_3766 | ID:63770990 groES\| chaperone Hsp10, affects cell division [Denitratisoma oestradiolicum Fahrbach xx] | -0.65928 | 0.00599 |
| DENOEST_v1_3765 | ID:63770989 groEL\| chaperone Hsp60, peptide-dependent ATPase, heat shock protein [Denitratisoma oestradiolicum Fahrbach xx] | -0.44399 | 0.023948 |
| DENOEST_v1_3764 | ID:63770988 fadE\| acyl coenzyme A dehydrogenase [Denitratisoma oestradiolicum Fahrbach xx] | 1.184913 | 0.007603 |
| DENOEST_v1_3748 | ID:63770972 conserved protein of unknown function [Denitratisoma oestradiolicum Fahrbach xx] | -0.80544 | 0.041212 |
| DENOEST_v1_3745 | ID:63770969 acrF\| multidrug efflux system protein [Denitratisoma oestradiolicum Fahrbach xx] | 0.998605 | 0.017887 |
| DENOEST_v1_3740 | ID:63770964 Copper ABC transporter substrate-binding protein [Denitratisoma oestradiolicum Fahrbach xx] | 3.065319 | 0.013261 |
| DENOEST_v1_3736 | ID:63770960 LysR family transcriptional regulator [Denitratisoma oestradiolicum Fahrbach xx] | -0.74012 | 0.002646 |
| DENOEST_v1_3714 | ID:63770938 Flavin-dependent oxidoreductase, luciferase family (Includes alkanesulfonate monooxygenase SsuD and methylene tetrahydromethanopterin reductase) [Denitratisoma oestradiolicum Fahrbach xx] | 0.655114 | 0.015103 |
| DENOEST_v1_3713 | ID:63770937 Ketosteroid isomerase [Denitratisoma oestradiolicum Fahrbach xx] | 0.750743 | 0.005853 |
| DENOEST_v1_3712 | ID:63770936 cofE\| Coenzyme F420:L-glutamate ligase [Denitratisoma oestradiolicum Fahrbach xx] | -0.89746 | 0.002361 |
| DENOEST_v1_3711 | ID:63770935 cofD\| 2-phospho-L-lactate transferase [Denitratisoma oestradiolicum Fahrbach xx] | -0.42843 | 0.024552 |
| DENOEST_v1_3707 | ID:63770931 DNA topoisomerase III [Denitratisoma oestradiolicum Fahrbach xx] | -0.21158 | 0.030061 |
| DENOEST_v1_3703 | ID:63770927 def\| peptide deformylase [Denitratisoma oestradiolicum Fahrbach xx] | -0.44385 | 0.017955 |
| DENOEST_v1_3698 | ID:63770922 AAA family ATPase [Denitratisoma oestradiolicum Fahrbach xx] | -0.31915 | 0.018244 |
| DENOEST_v1_3697 | ID:63770921 Membrane-bound lytic murein transglycosylase A [Denitratisoma oestradiolicum Fahrbach xx] | -0.6238 | 0.012948 |
| DENOEST_v1_3694 | ID:63770918 aac\| Aculeacin-A acylase [Denitratisoma oestradiolicum Fahrbach xx] | 2.33869 | 0.006694 |
| DENOEST_v1_3675 | ID:63770899 qbdA\| Quinohemoprotein alcohol dehydrogenase ADH IIB [Denitratisoma oestradiolicum Fahrbach xx] | 0.685003 | 0.000416 |
| DENOEST_v1_3672 | ID:63770896 Arylsulfatase [Denitratisoma oestradiolicum Fahrbach xx] | 1.225417 | 5.32E-05 |
| DENOEST_v1_3671 | ID:63770895 conserved protein of unknown function [Denitratisoma oestradiolicum Fahrbach xx] | 1.272327 | 8.19E-05 |
| DENOEST_v1_3667 | ID:63770891 3-oxosteroid 1-dehydrogenase [Denitratisoma oestradiolicum Fahrbach xx] | 0.473009 | 0.014132 |
| DENOEST_v1_3665 | ID:63770889 putative Luciferase family protein [Denitratisoma oestradiolicum Fahrbach xx] | 0.906183 | 0.009992 |
| DENOEST_v1_3658 | ID:63770882 Carbon monoxide dehydrogenase [Denitratisoma oestradiolicum Fahrbach xx] | 2.501348 | 8.89E-07 |
| DENOEST_v1_3657 | ID:63770881 conserved protein of unknown function [Denitratisoma oestradiolicum Fahrbach xx] | 1.189224 | 0.00035 |
| DENOEST_v1_3656 | ID:63770880 Transporter [Denitratisoma oestradiolicum Fahrbach xx] | 3.125758 | 0.008482 |
| DENOEST_v1_3655 | ID:63770879 conserved protein of unknown function [Denitratisoma oestradiolicum Fahrbach xx] | 2.842911 | 0.004679 |
| DENOEST_v1_3654 | ID:63770878 Transcriptional regulator, LuxR-family [Denitratisoma oestradiolicum Fahrbach xx] | 0.488831 | 0.035523 |
| DENOEST_v1_3651 | ID:63770875 conserved protein of unknown function [Denitratisoma oestradiolicum Fahrbach xx] | -2.12011 | 0.000876 |
| DENOEST_v1_3650 | ID:63770874 conserved protein of unknown function [Denitratisoma oestradiolicum Fahrbach xx] | -3.55117 | 8.71E-05 |
| DENOEST_v1_3649 | ID:63770873 conserved protein of unknown function [Denitratisoma oestradiolicum Fahrbach xx] | -2.60369 | 0.000761 |
| DENOEST_v1_3646 | ID:63770870 nsrR\| HTH-type transcriptional regulator NsrR [Denitratisoma oestradiolicum Fahrbach xx] | -1.22945 | 0.000817 |
| DENOEST_v1_3644 | ID:63770868 ftsY\| Signal recognition particle receptor FtsY [Denitratisoma oestradiolicum Fahrbach xx] | -1.01058 | 0.000199 |
| DENOEST_v1_3637 | ID:63770861 conserved exported protein of unknown function [Denitratisoma oestradiolicum Fahrbach xx] | -0.27718 | 0.026296 |
| DENOEST_v1_3633 | ID:63770857 rplY\| 50S ribosomal protein L25 [Denitratisoma oestradiolicum Fahrbach xx] | -0.97965 | 0.000922 |
| DENOEST_v1_3632 | ID:63770856 pth\| peptidyl-tRNA hydrolase [Denitratisoma oestradiolicum Fahrbach xx] | -0.30004 | 0.040721 |
| DENOEST_v1_3624 | ID:63770848 hemE\| uroporphyrinogen decarboxylase [Denitratisoma oestradiolicum Fahrbach xx] | -0.61933 | 0.002028 |
| DENOEST_v1_3615 | ID:63770839 Thiol:disulfide interchange protein [Denitratisoma oestradiolicum Fahrbach xx] | -0.70721 | 0.008096 |
| DENOEST_v1_3614 | ID:63770838 SAM-dependent methyltransferase protein [Denitratisoma oestradiolicum Fahrbach xx] | -0.79113 | 0.027351 |
| DENOEST_v1_3609 | ID:63770833 proA\| Gamma-glutamyl phosphate reductase [Denitratisoma oestradiolicum Fahrbach xx] | 0.16647 | 0.045224 |
| DENOEST_v1_3607 | ID:63770831 lptE\| LPS-assembly lipoprotein LptE [Denitratisoma oestradiolicum Fahrbach xx] | -0.49114 | 0.01804 |
| DENOEST_v1_3606 | ID:63770830 exported protein of unknown function [Denitratisoma oestradiolicum Fahrbach xx] | -0.50585 | 0.015344 |
| DENOEST_v1_3605 | ID:63770829 leuS\| leucyl-tRNA synthetase [Denitratisoma oestradiolicum Fahrbach xx] | -0.9026 | 0.006059 |
| DENOEST_v1_3604 | ID:63770828 conserved exported protein of unknown function [Denitratisoma oestradiolicum Fahrbach xx] | -0.6981 | 0.002451 |
| DENOEST_v1_3598 | ID:63770822 Cobyric acid synthase [Denitratisoma oestradiolicum Fahrbach xx] | -1.81197 | 0.001252 |
| DENOEST_v1_3593 | ID:63770817 Porin Gram-negative type [Denitratisoma oestradiolicum Fahrbach xx] | -1.0442 | 0.000829 |
| DENOEST_v1_3590 | ID:63770814 metG\| methionyl-tRNA synthetase [Denitratisoma oestradiolicum Fahrbach xx] | -1.0303 | 0.034355 |
| DENOEST_v1_3589 | ID:63770813 mrp\| antiporter inner membrane protein [Denitratisoma oestradiolicum Fahrbach xx] | -0.48549 | 0.026847 |
| DENOEST_v1_3582 | ID:63770806 pgi\| glucosephosphate isomerase [Denitratisoma oestradiolicum Fahrbach xx] | 0.868132 | 0.001696 |
| DENOEST_v1_3580 | ID:63770804 Glycosyl hydrolase [Denitratisoma oestradiolicum Fahrbach xx] | 0.570038 | 0.003291 |
| DENOEST_v1_3578 | ID:63770802 glgC\| glucose-1-phosphate adenylyltransferase [Denitratisoma oestradiolicum Fahrbach xx] | 0.809076 | 0.001123 |
| DENOEST_v1_3577 | ID:63770801 glgB\| 1,4-alpha-glucan branching enzyme [Denitratisoma oestradiolicum Fahrbach xx] | 0.839828 | 0.037727 |
| DENOEST_v1_3574 | ID:63770798 glk\| Glucokinase [Denitratisoma oestradiolicum Fahrbach xx] | 0.529168 | 0.049639 |
| DENOEST_v1_3573 | ID:63770797 thyA\| thymidylate synthetase [Denitratisoma oestradiolicum Fahrbach xx] | 0.231332 | 0.036275 |
| DENOEST_v1_3571 | ID:63770795 conserved protein of unknown function [Denitratisoma oestradiolicum Fahrbach xx] | -0.43893 | 0.006432 |
| DENOEST_v1_3570 | ID:63770794 mogA\| molybdochelatase MogA, involved in Moco biosynthesis [Denitratisoma oestradiolicum Fahrbach xx] | -0.43102 | 0.007863 |
| DENOEST_v1_3569 | ID:63770793 yjgA\| conserved hypothetical protein [Denitratisoma oestradiolicum Fahrbach xx] | -0.44541 | 0.024497 |
| DENOEST_v1_3568 | ID:63770792 pmbA\| peptidase required for the maturation and secretion of the antibiotic peptide MccB17 [Denitratisoma oestradiolicum Fahrbach xx] | -0.45169 | 0.005614 |
| DENOEST_v1_3564 | ID:63770788 glyA\| serine hydroxymethyltransferase [Denitratisoma oestradiolicum Fahrbach xx] | -0.54425 | 0.005243 |
| DENOEST_v1_3563 | ID:63770787 nrdR\| transcriptional repressor of nrd genes [Denitratisoma oestradiolicum Fahrbach xx] | -0.4269 | 0.004883 |
| DENOEST_v1_3561 | ID:63770785 Uncharacterized outer-membrane protein y4mB [Denitratisoma oestradiolicum Fahrbach xx] | -2.30922 | 0.000878 |
| DENOEST_v1_3560 | ID:63770784 2-oxoglutarate ferredoxin oxidoreductase subunit beta [Denitratisoma oestradiolicum Fahrbach xx] | -0.52827 | 0.000398 |
| DENOEST_v1_3559 | ID:63770783 korA\| 2-ketoglutarate: NADP oxidoreductase subunit alpha [Denitratisoma oestradiolicum Fahrbach xx] | -0.42519 | 0.002639 |
| DENOEST_v1_3558 | ID:63770782 korC\| 2-ketoglutarate: NADP oxidoreductase subunit gamma [Denitratisoma oestradiolicum Fahrbach xx] | -0.50841 | 0.012826 |
| DENOEST_v1_3553 | ID:63770777 phnA\| conserved hypothetical protein [Denitratisoma oestradiolicum Fahrbach xx] | -1.02778 | 0.008273 |
| DENOEST_v1_3544 | ID:63770768 conserved exported protein of unknown function [Denitratisoma oestradiolicum Fahrbach xx] | 1.507117 | 0.00239 |
| DENOEST_v1_3535 | ID:63770759 conserved protein of unknown function [Denitratisoma oestradiolicum Fahrbach xx] | -0.6775 | 0.027414 |
| DENOEST_v1_3525 | ID:63770749 VOC family protein [Denitratisoma oestradiolicum Fahrbach xx] | -0.51468 | 0.032467 |
| DENOEST_v1_3518 | ID:63770742 conserved protein of unknown function [Denitratisoma oestradiolicum Fahrbach xx] | -1.72326 | 0.000839 |
| DENOEST_v1_3516 | ID:63770740 putative TonB-dependent receptor [Denitratisoma oestradiolicum Fahrbach xx] | 1.416476 | 0.002685 |
| DENOEST_v1_3513 | ID:63770737 putative TonB-dependent receptor [Denitratisoma oestradiolicum Fahrbach xx] | 1.701233 | 0.002638 |
| DENOEST_v1_3511 | ID:63770735 conserved exported protein of unknown function [Denitratisoma oestradiolicum Fahrbach xx] | 2.674607 | 0.000152 |
| DENOEST_v1_3509 | ID:63770733 pcp\| Outer membrane lipoprotein pcp [Denitratisoma oestradiolicum Fahrbach xx] | -1.79022 | 0.001108 |
| DENOEST_v1_3508 | ID:63770732 PfkB domain-containing protein [Denitratisoma oestradiolicum Fahrbach xx] | -1.28189 | 0.002913 |
| DENOEST_v1_3507 | ID:63770731 conserved protein of unknown function [Denitratisoma oestradiolicum Fahrbach xx] | -0.82646 | 0.03516 |
| DENOEST_v1_3506 | ID:63770730 prmA\| methylase for 50S ribosomal subunit protein L11 [Denitratisoma oestradiolicum Fahrbach xx] | -0.94445 | 0.001691 |
| DENOEST_v1_3505 | ID:63770729 accC\| biotin carboxylase (A subunit of acetyl-CoA carboxylase) [Denitratisoma oestradiolicum Fahrbach xx] | -0.561 | 0.003121 |
| DENOEST_v1_3504 | ID:63770728 accB\| acetyl CoA carboxylase, BCCP subunit [Denitratisoma oestradiolicum Fahrbach xx] | -0.89808 | 0.002798 |
| DENOEST_v1_3500 | ID:63770724 mpl\| UDP-N-acetylmuramate:L-alanyl-gamma-D-glutamyl-meso-diaminopimelate ligase [Denitratisoma oestradiolicum Fahrbach xx] | -0.70078 | 0.022972 |
| DENOEST_v1_3499 | ID:63770723 conserved exported protein of unknown function [Denitratisoma oestradiolicum Fahrbach xx] | -0.65334 | 0.013335 |
| DENOEST_v1_3498 | ID:63770722 Cytochrome c4 [Denitratisoma oestradiolicum Fahrbach xx] | 0.771281 | 0.003704 |
| DENOEST_v1_3495 | ID:63770719 Cytochrome c oxidase aa3, subunit II [Denitratisoma oestradiolicum Fahrbach xx] | 1.169579 | 0.002941 |
| DENOEST_v1_3494 | ID:63770718 ctaD\| Cytochrome c oxidase subunit 1 [Denitratisoma oestradiolicum Fahrbach xx] | 17.06517 | 0.043996 |
| DENOEST_v1_3492 | ID:63770716 Methyl-accepting chemotaxis sensory transducer [Denitratisoma oestradiolicum Fahrbach xx] | -1.48254 | 0.007694 |
| DENOEST_v1_3491 | ID:63770715 rpoD\| RNA polymerase, sigma 70 (sigma D) factor [Denitratisoma oestradiolicum Fahrbach xx] | -1.08179 | 0.00059 |
| DENOEST_v1_3490 | ID:63770714 dnaG\| DNA primase [Denitratisoma oestradiolicum Fahrbach xx] | 0.636115 | 0.009881 |
| DENOEST_v1_3488 | ID:63770712 rpsU\| 30S ribosomal subunit protein S21 [Denitratisoma oestradiolicum Fahrbach xx] | -1.56439 | 6.82E-05 |
| DENOEST_v1_3473 | ID:63770697 gmk\| guanylate kinase [Denitratisoma oestradiolicum Fahrbach xx] | 1.671217 | 0.001232 |
| DENOEST_v1_3472 | ID:63770696 yicC\| conserved hypothetical protein [Denitratisoma oestradiolicum Fahrbach xx] | -0.56563 | 0.005177 |
| DENOEST_v1_3469 | ID:63770693 Periplasmic nitrate reductase, electron transfer subunit [Denitratisoma oestradiolicum Fahrbach xx] | 1.481879 | 0.003761 |
| DENOEST_v1_3467 | ID:63770691 napG\| Ferredoxin-type protein NapG [Denitratisoma oestradiolicum Fahrbach xx] | 2.774276 | 0.001635 |
| DENOEST_v1_3466 | ID:63770690 napA\| nitrate reductase, periplasmic, large subunit [Denitratisoma oestradiolicum Fahrbach xx] | 1.300586 | 0.006507 |
| DENOEST_v1_3464 | ID:63770688 rph\| Ribonuclease PH [Denitratisoma oestradiolicum Fahrbach xx] | -0.76598 | 0.02613 |
| DENOEST_v1_3459 | ID:63770683 conserved protein of unknown function [Denitratisoma oestradiolicum Fahrbach xx] | 1.664491 | 1.13E-05 |
| DENOEST_v1_3452 | ID:63770676 Benzaldehyde lyase [Denitratisoma oestradiolicum Fahrbach xx] | 0.368392 | 0.005236 |
| DENOEST_v1_3449 | ID:63770673 Cytochrome c4 [Denitratisoma oestradiolicum Fahrbach xx] | -0.64377 | 0.002101 |
| DENOEST_v1_3448 | ID:63770672 engB\| putative GTP-binding protein EngB [Denitratisoma oestradiolicum Fahrbach xx] | -0.62132 | 0.000868 |
| DENOEST_v1_3447 | ID:63770671 hemB\| Delta-aminolevulinic acid dehydratase [Denitratisoma oestradiolicum Fahrbach xx] | -0.28951 | 0.023721 |
| DENOEST_v1_3445 | ID:63770669 argB\| Acetylglutamate kinase [Denitratisoma oestradiolicum Fahrbach xx] | -0.3306 | 0.001436 |
| DENOEST_v1_3444 | ID:63770668 Diguanylate cyclase/phosphodiesterase (GGDEF & EAL domains) withPAS/PAC sensor (fragment) [Denitratisoma oestradiolicum Fahrbach xx] | -4.21421 | 0.000561 |
| DENOEST_v1_3440 | ID:63770664 conserved protein of unknown function [Denitratisoma oestradiolicum Fahrbach xx] | -0.27647 | 0.015812 |
| DENOEST_v1_3436 | ID:63770660 conserved exported protein of unknown function [Denitratisoma oestradiolicum Fahrbach xx] | -1.24375 | 0.011975 |
| DENOEST_v1_3433 | ID:63770657 queE\| 7-carboxy-7-deazaguanine synthase [Denitratisoma oestradiolicum Fahrbach xx] | -0.60109 | 0.033456 |
| DENOEST_v1_3428 | ID:63770652 tolR\| Tol-Pal system protein TolR [Denitratisoma oestradiolicum Fahrbach xx] | -0.63047 | 0.003434 |
| DENOEST_v1_3425 | ID:63770649 conserved protein of unknown function [Denitratisoma oestradiolicum Fahrbach xx] | -0.41815 | 0.001024 |
| DENOEST_v1_3422 | ID:63770646 ruvB\| holliday junction helicase, subunit B [Denitratisoma oestradiolicum Fahrbach xx] | -0.46895 | 0.005897 |
| DENOEST_v1_3421 | ID:63770645 conserved protein of unknown function [Denitratisoma oestradiolicum Fahrbach xx] | -1.59152 | 0.019408 |
| DENOEST_v1_3420 | ID:63770644 ruvA\| component of RuvABC resolvasome, regulatory subunit [Denitratisoma oestradiolicum Fahrbach xx] | -0.80376 | 0.006253 |
| DENOEST_v1_3416 | ID:63770640 yebC\| conserved hypothetical protein [Denitratisoma oestradiolicum Fahrbach xx] | -0.63626 | 0.003459 |
| DENOEST_v1_3409 | ID:63770633 putative TonB-dependent receptor [Denitratisoma oestradiolicum Fahrbach xx] | -1.09321 | 3.12E-05 |
| DENOEST_v1_3408 | ID:63770632 putative TonB-dependent receptor [Denitratisoma oestradiolicum Fahrbach xx] | 0.360794 | 0.003665 |
| DENOEST_v1_3407 | ID:63770631 putative enzyme [Denitratisoma oestradiolicum Fahrbach xx] | -0.48577 | 0.023824 |
| DENOEST_v1_3406 | ID:63770630 metK\| methionine adenosyltransferase 1 [Denitratisoma oestradiolicum Fahrbach xx] | -0.94418 | 0.005454 |
| DENOEST_v1_3402 | ID:63770626 cheV\| Chemotaxis protein CheV-like [Denitratisoma oestradiolicum Fahrbach xx] | -0.21164 | 0.045971 |
| DENOEST_v1_3401 | ID:63770625 cheY\| chemotaxis regulator transmitting signal to flagellar motor component [Denitratisoma oestradiolicum Fahrbach xx] | -0.66599 | 0.049883 |
| DENOEST_v1_3396 | ID:63770620 Flagellar biosynthesis protein FlhF [Denitratisoma oestradiolicum Fahrbach xx] | -0.7029 | 0.00149 |
| DENOEST_v1_3389 | ID:63770613 Flagella basal body P-ring formation protein FlgA [Denitratisoma oestradiolicum Fahrbach xx] | 1.610848 | 0.007606 |
| DENOEST_v1_3385 | ID:63770609 Flagellar hook protein FlgE [Denitratisoma oestradiolicum Fahrbach xx] | 1.480465 | 0.00438 |
| DENOEST_v1_3382 | ID:63770606 flgH\| flagellar protein of basal-body outer-membrane L ring [Denitratisoma oestradiolicum Fahrbach xx] | 1.974247 | 0.005662 |
| DENOEST_v1_3370 | ID:63770594 Flagellar protein FliL [Denitratisoma oestradiolicum Fahrbach xx] | 0.635959 | 0.017978 |
| DENOEST_v1_3364 | ID:63770588 fliG\| flagellar motor switching and energizing component [Denitratisoma oestradiolicum Fahrbach xx] | -1.16898 | 0.033561 |
| DENOEST_v1_3359 | ID:63770583 Adenylate/guanylate cyclase [Denitratisoma oestradiolicum Fahrbach xx] | 3.825307 | 0.006282 |
| DENOEST_v1_3357 | ID:63770581 parE\| DNA topoisomerase IV, subunit B [Denitratisoma oestradiolicum Fahrbach xx] | -0.39935 | 0.040752 |
| DENOEST_v1_3354 | ID:63770578 conserved protein of unknown function [Denitratisoma oestradiolicum Fahrbach xx] | 15.03472 | 0.006508 |
| DENOEST_v1_3350 | ID:63770574 putative Flagellin [Denitratisoma oestradiolicum Fahrbach xx] | 0.804364 | 0.001725 |
| DENOEST_v1_3348 | ID:63770572 putative Flagellin [Denitratisoma oestradiolicum Fahrbach xx] | 0.844643 | 0.001049 |
| DENOEST_v1_3344 | ID:63770568 Methyltransferase, FkbM family [Denitratisoma oestradiolicum Fahrbach xx] | -0.14874 | 0.013393 |
| DENOEST_v1_3342 | ID:63770566 SAM-dependent methyltransferase [Denitratisoma oestradiolicum Fahrbach xx] | -0.26852 | 0.046251 |
| DENOEST_v1_3341 | ID:63770565 rfbG\| CDP-glucose 4,6-dehydratase [Denitratisoma oestradiolicum Fahrbach xx] | 0.147207 | 0.037003 |
| DENOEST_v1_3336 | ID:63770560 conserved protein of unknown function [Denitratisoma oestradiolicum Fahrbach xx] | 1.004494 | 0.009481 |
| DENOEST_v1_3335 | ID:63770559 narH\| Respiratory nitrate reductase subunit beta [Denitratisoma oestradiolicum Fahrbach xx] | 3.410228 | 0.004054 |
| DENOEST_v1_3334 | ID:63770558 conserved exported protein of unknown function [Denitratisoma oestradiolicum Fahrbach xx] | 0.946284 | 0.03859 |
| DENOEST_v1_3331 | ID:63770555 conserved protein of unknown function [Denitratisoma oestradiolicum Fahrbach xx] | 0.941272 | 8.34E-05 |
| DENOEST_v1_3329 | ID:63770553 putative TonB-dependent receptor [Denitratisoma oestradiolicum Fahrbach xx] | 1.654269 | 0.000715 |
| DENOEST_v1_3324 | ID:63770548 conserved protein of unknown function [Denitratisoma oestradiolicum Fahrbach xx] | 0.68414 | 0.002032 |
| DENOEST_v1_3323 | ID:63770547 putative enzyme [Denitratisoma oestradiolicum Fahrbach xx] | 0.890861 | 0.001404 |
| DENOEST_v1_3308 | ID:63770532 lipA\| lipoate synthase [Denitratisoma oestradiolicum Fahrbach xx] | -0.55279 | 0.01134 |
| DENOEST_v1_3305 | ID:63770529 D-amino acid aminotransferase [Denitratisoma oestradiolicum Fahrbach xx] | -0.50469 | 0.00145 |
| DENOEST_v1_3304 | ID:63770528 dacC\| D-alanyl-D-alanine carboxypeptidase DacC [Denitratisoma oestradiolicum Fahrbach xx] | -1.01009 | 0.004668 |
| DENOEST_v1_3295 | ID:63770519 yliK\| methylmalonyl-CoA mutase [Denitratisoma oestradiolicum Fahrbach xx] | 0.1994 | 0.031823 |
| DENOEST_v1_3292 | ID:63770516 cpnA\| Cyclopentanol dehydrogenase [Denitratisoma oestradiolicum Fahrbach xx] | 1.021496 | 0.000241 |
| DENOEST_v1_3287 | ID:63770511 Polyphosphate:AMP phosphotransferase [Denitratisoma oestradiolicum Fahrbach xx] | 0.300855 | 0.032494 |
| DENOEST_v1_3282 | ID:63770506 tktA\| transketolase 1, thiamin-binding [Denitratisoma oestradiolicum Fahrbach xx] | -0.5331 | 0.003714 |
| DENOEST_v1_3281 | ID:63770505 gapA\| glyceraldehyde-3-phosphate dehydrogenase A [Denitratisoma oestradiolicum Fahrbach xx] | -1.10613 | 0.002199 |
| DENOEST_v1_3280 | ID:63770504 pgk\| phosphoglycerate kinase [Denitratisoma oestradiolicum Fahrbach xx] | -0.60301 | 0.001141 |
| DENOEST_v1_3279 | ID:63770503 pykA\| pyruvate kinase II [Denitratisoma oestradiolicum Fahrbach xx] | -1.10371 | 0.003483 |
| DENOEST_v1_3278 | ID:63770502 fda\| fructose-1,6-bisphosphate aldolase, class II [Denitratisoma oestradiolicum Fahrbach xx] | -0.67402 | 0.000597 |
| DENOEST_v1_3275 | ID:63770499 yjcH\| conserved hypothetical protein; putative inner membrane protein involved in acetate transport [Denitratisoma oestradiolicum Fahrbach xx] | -7.02033 | 1.29E-05 |
| DENOEST_v1_3274 | ID:63770498 putative sodium:solute symporter [Denitratisoma oestradiolicum Fahrbach xx] | -4.80422 | 0.002973 |
| DENOEST_v1_3271 | ID:63770495 conserved exported protein of unknown function [Denitratisoma oestradiolicum Fahrbach xx] | 18.27726 | 7.08E-06 |
| DENOEST_v1_3267 | ID:63770491 conserved protein of unknown function [Denitratisoma oestradiolicum Fahrbach xx] | -17.5953 | 0.014728 |
| DENOEST_v1_3265 | ID:63770489 conserved membrane protein of unknown function [Denitratisoma oestradiolicum Fahrbach xx] | -1.80508 | 0.001519 |
| DENOEST_v1_3264 | ID:63770488 nirQ\| Denitrification regulatory protein NirQ [Denitratisoma oestradiolicum Fahrbach xx] | -22.1057 | 0.000421 |
| DENOEST_v1_3261 | ID:63770485 norB\| Nitric oxide reductase subunit B [Denitratisoma oestradiolicum Fahrbach xx] | -2.25754 | 0.03095 |
| DENOEST_v1_3260 | ID:63770484 norC\| Nitric oxide reductase subunit C [Denitratisoma oestradiolicum Fahrbach xx] | -2.43806 | 0.00268 |
| DENOEST_v1_3259 | ID:63770483 Exonuclease RNase T and DNA polymerase III [Denitratisoma oestradiolicum Fahrbach xx] | -19.9318 | 0.000766 |
| DENOEST_v1_3258 | ID:63770482 Cyclic nucleotide-binding protein [Denitratisoma oestradiolicum Fahrbach xx] | -4.91603 | 0.001302 |
| DENOEST_v1_3255 | ID:63770479 Sugar transport protein [Denitratisoma oestradiolicum Fahrbach xx] | -1.69764 | 0.001442 |
| DENOEST_v1_3254 | ID:63770478 Metallophosphatase family protein [Denitratisoma oestradiolicum Fahrbach xx] | -1.26537 | 0.000125 |
| DENOEST_v1_3253 | ID:63770477 putative serine/threonine-protein kinase [Denitratisoma oestradiolicum Fahrbach xx] | -2.70423 | 0.000806 |
| DENOEST_v1_3243 | ID:63770467 metH\| homocysteine-N5-methyltetrahydrofolate transmethylase, B12-dependent [Denitratisoma oestradiolicum Fahrbach xx] | 1.369103 | 0.000222 |
| DENOEST_v1_3242 | ID:63770466 hppA\| K(+)-insensitive pyrophosphate-energized proton pump [Denitratisoma oestradiolicum Fahrbach xx] | 1.087442 | 0.00184 |
| DENOEST_v1_3239 | ID:63770463 adk\| adenylate kinase [Denitratisoma oestradiolicum Fahrbach xx] | -0.3037 | 0.044088 |
| DENOEST_v1_3238 | ID:63770462 kdsB\| 3-deoxy-manno-octulosonate cytidylyltransferase [Denitratisoma oestradiolicum Fahrbach xx] | 0.17972 | 0.036382 |
| DENOEST_v1_3231 | ID:63770455 conserved exported protein of unknown function [Denitratisoma oestradiolicum Fahrbach xx] | 20.31483 | 0.000171 |
| DENOEST_v1_3229 | ID:63770453 sodB\| superoxide dismutase, Fe [Denitratisoma oestradiolicum Fahrbach xx] | -1.0687 | 0.001939 |
| DENOEST_v1_3225 | ID:63770449 Amidohydrolase [Denitratisoma oestradiolicum Fahrbach xx] | -0.37749 | 0.011265 |
| DENOEST_v1_3219 | ID:63770443 Glycosyl transferase family 1 [Denitratisoma oestradiolicum Fahrbach xx] | -0.26903 | 0.004645 |
| DENOEST_v1_3210 | ID:63770434 conserved protein of unknown function [Denitratisoma oestradiolicum Fahrbach xx] | -0.8321 | 0.003151 |
| DENOEST_v1_3209 | ID:63770433 Glycosyl transferase family 9 [Denitratisoma oestradiolicum Fahrbach xx] | -0.69313 | 0.010005 |
| DENOEST_v1_3207 | ID:63770431 polA\| fused DNA polymerase I 5'->3' exonuclease ; 3'->5' polymerase ; 3'->5' exonuclease [Denitratisoma oestradiolicum Fahrbach xx] | -0.39418 | 0.004791 |
| DENOEST_v1_3206 | ID:63770430 Cytokinin riboside 5'-monophosphate phosphoribohydrolase [Denitratisoma oestradiolicum Fahrbach xx] | -0.10806 | 0.027198 |
| DENOEST_v1_3201 | ID:63770425 conserved protein of unknown function [Denitratisoma oestradiolicum Fahrbach xx] | -0.79093 | 0.006698 |
| DENOEST_v1_3199 | ID:63770423 yhdE\| putative septum formation protein [Denitratisoma oestradiolicum Fahrbach xx] | -0.53544 | 0.024595 |
| DENOEST_v1_3196 | ID:63770420 rng\| ribonuclease G [Denitratisoma oestradiolicum Fahrbach xx] | -0.38825 | 0.017914 |
| DENOEST_v1_3188 | ID:63770412 metW\| Methionine biosynthesis protein MetW [Denitratisoma oestradiolicum Fahrbach xx] | -0.80577 | 0.040467 |
| DENOEST_v1_3185 | ID:63770409 conserved protein of unknown function [Denitratisoma oestradiolicum Fahrbach xx] | 2.762995 | 0.001192 |
| DENOEST_v1_3174 | ID:63770398 conserved exported protein of unknown function [Denitratisoma oestradiolicum Fahrbach xx] | -0.65103 | 0.002134 |
| DENOEST_v1_3164 | ID:63770388 pckG\| Phosphoenolpyruvate carboxykinase [GTP] [Denitratisoma oestradiolicum Fahrbach xx] | -1.18975 | 1.85E-05 |
| DENOEST_v1_3159 | ID:63770383 hbd\| 3-hydroxybutyryl-CoA dehydrogenase [Denitratisoma oestradiolicum Fahrbach xx] | -0.6804 | 4.34E-06 |
| DENOEST_v1_3148 | ID:63770372 rplK\| 50S ribosomal protein L11 [Denitratisoma oestradiolicum Fahrbach xx] | -0.9821 | 0.031478 |
| DENOEST_v1_3147 | ID:63770371 rplA\| 50S ribosomal protein L1 [Denitratisoma oestradiolicum Fahrbach xx] | -1.17931 | 0.0003 |
| DENOEST_v1_3146 | ID:63770370 rplJ\| 50S ribosomal subunit protein L10 [Denitratisoma oestradiolicum Fahrbach xx] | -1.10197 | 0.003548 |
| DENOEST_v1_3145 | ID:63770369 rplL\| 50S ribosomal protein L7/L12 [Denitratisoma oestradiolicum Fahrbach xx] | -1.51413 | 0.002974 |
| DENOEST_v1_3144 | ID:63770368 rpoB\| DNA-directed RNA polymerase beta chain (Transcriptase beta chain) (RNA polymerase beta subunit) [Denitratisoma oestradiolicum Fahrbach xx] | -1.44097 | 0.0305 |
| DENOEST_v1_3143 | ID:63770367 rpoC\| DNA-directed RNA polymerase beta' chain (Transcriptase beta' chain) (RNA polymerase beta' subunit) [Denitratisoma oestradiolicum Fahrbach xx] | -1.18718 | 0.036669 |
| DENOEST_v1_3141 | ID:63770365 rpsG\| 30S ribosomal protein S7 [Denitratisoma oestradiolicum Fahrbach xx] | -1.25751 | 3.06E-05 |
| DENOEST_v1_3140 | ID:63770364 fusA\| protein chain elongation factor EF-G, GTP-binding [Denitratisoma oestradiolicum Fahrbach xx] | -0.67398 | 0.003399 |
| DENOEST_v1_3139 | ID:63770363 tufB\| protein chain elongation factor EF-Tu, possible GTP-binding factor (duplicate of tufA) [Denitratisoma oestradiolicum Fahrbach xx] | -0.53927 | 0.000983 |
| DENOEST_v1_3138 | ID:63770362 rpsJ\| 30S ribosomal protein S10 [Denitratisoma oestradiolicum Fahrbach xx] | -1.40135 | 2.76E-05 |
| DENOEST_v1_3137 | ID:63770361 rplC\| 50S ribosomal subunit protein L3 [Denitratisoma oestradiolicum Fahrbach xx] | -1.21723 | 0.000777 |
| DENOEST_v1_3136 | ID:63770360 rplD\| 50S ribosomal subunit protein L4 [Denitratisoma oestradiolicum Fahrbach xx] | -1.30814 | 1.87E-05 |
| DENOEST_v1_3135 | ID:63770359 rplW\| 50S ribosomal subunit protein L23 [Denitratisoma oestradiolicum Fahrbach xx] | -0.76471 | 0.03111 |
| DENOEST_v1_3133 | ID:63770357 rpsS\| 30S ribosomal protein S19 [Denitratisoma oestradiolicum Fahrbach xx] | -1.30215 | 0.002533 |
| DENOEST_v1_3132 | ID:63770356 rplV\| 50S ribosomal protein L22 [Denitratisoma oestradiolicum Fahrbach xx] | -1.26744 | 0.001642 |
| DENOEST_v1_3131 | ID:63770355 rpsC\| 30S ribosomal subunit protein S3 [Denitratisoma oestradiolicum Fahrbach xx] | -0.93323 | 0.010916 |
| DENOEST_v1_3130 | ID:63770354 rplP\| 50S ribosomal protein L16 [Denitratisoma oestradiolicum Fahrbach xx] | -0.95716 | 0.000928 |
| DENOEST_v1_3128 | ID:63770352 rpsQ\| 30S ribosomal protein S17 [Denitratisoma oestradiolicum Fahrbach xx] | -1.02534 | 0.013784 |
| DENOEST_v1_3127 | ID:63770351 rplN\| 50S ribosomal protein L14 [Denitratisoma oestradiolicum Fahrbach xx] | -1.14761 | 0.001296 |
| DENOEST_v1_3125 | ID:63770349 rplE\| 50S ribosomal protein L5 [Denitratisoma oestradiolicum Fahrbach xx] | -1.19133 | 0.00771 |
| DENOEST_v1_3124 | ID:63770348 rpsN\| 30S ribosomal protein S14 [Denitratisoma oestradiolicum Fahrbach xx] | -0.93503 | 0.005607 |
| DENOEST_v1_3123 | ID:63770347 rpsH\| 30S ribosomal subunit protein S8 [Denitratisoma oestradiolicum Fahrbach xx] | -1.09609 | 7.31E-05 |
| DENOEST_v1_3122 | ID:63770346 rplF\| 50S ribosomal subunit protein L6 [Denitratisoma oestradiolicum Fahrbach xx] | -1.2684 | 0.000182 |
| DENOEST_v1_3121 | ID:63770345 rplR\| 50S ribosomal protein L18 [Denitratisoma oestradiolicum Fahrbach xx] | -0.98998 | 0.000628 |
| DENOEST_v1_3120 | ID:63770344 rpsE\| 30S ribosomal subunit protein S5 [Denitratisoma oestradiolicum Fahrbach xx] | -1.12598 | 0.00015 |
| DENOEST_v1_3119 | ID:63770343 rpmD\| 50S ribosomal subunit protein L30 [Denitratisoma oestradiolicum Fahrbach xx] | -0.8426 | 0.034365 |
| DENOEST_v1_3118 | ID:63770342 rplO\| 50S ribosomal subunit protein L15 [Denitratisoma oestradiolicum Fahrbach xx] | -1.01459 | 0.004958 |
| DENOEST_v1_3117 | ID:63770341 secY\| preprotein translocase membrane subunit [Denitratisoma oestradiolicum Fahrbach xx] | 1.219123 | 0.02145 |
| DENOEST_v1_3116 | ID:63770340 infA\| translation initiation factor IF-1 [Denitratisoma oestradiolicum Fahrbach xx] | -0.59873 | 0.040184 |
| DENOEST_v1_3115 | ID:63770339 rpsM\| 30S ribosomal protein S13 [Denitratisoma oestradiolicum Fahrbach xx] | -0.80191 | 0.002593 |
| DENOEST_v1_3114 | ID:63770338 rpsK\| 30S ribosomal protein S11 [Denitratisoma oestradiolicum Fahrbach xx] | -0.99105 | 0.012169 |
| DENOEST_v1_3113 | ID:63770337 rpsD\| 30S ribosomal subunit protein S4 [Denitratisoma oestradiolicum Fahrbach xx] | -1.29036 | 3.72E-06 |
| DENOEST_v1_3112 | ID:63770336 rpoA\| RNA polymerase, alpha subunit [Denitratisoma oestradiolicum Fahrbach xx] | -0.67421 | 0.001414 |
| DENOEST_v1_3111 | ID:63770335 rplQ\| 50S ribosomal protein L17 [Denitratisoma oestradiolicum Fahrbach xx] | -1.4662 | 0.006785 |
| DENOEST_v1_3105 | ID:63770329 conserved exported protein of unknown function [Denitratisoma oestradiolicum Fahrbach xx] | 0.517344 | 0.021037 |
| DENOEST_v1_3102 | ID:63770326 yraO\| DnaA initiator-associating factor for replication initiation [Denitratisoma oestradiolicum Fahrbach xx] | -0.54594 | 0.02718 |
| DENOEST_v1_3099 | ID:63770323 yraL\| putative methyltransferase [Denitratisoma oestradiolicum Fahrbach xx] | -0.65176 | 0.039966 |
| DENOEST_v1_3097 | ID:63770321 conserved exported protein of unknown function [Denitratisoma oestradiolicum Fahrbach xx] | -0.89518 | 0.016012 |
| DENOEST_v1_3094 | ID:63770318 mraZ\| conserved hypothetical protein [Denitratisoma oestradiolicum Fahrbach xx] | -0.27125 | 0.02389 |
| DENOEST_v1_3091 | ID:63770315 ftsI\| transpeptidase involved in septal peptidoglycan synthesis (penicillin-binding protein 3) [Denitratisoma oestradiolicum Fahrbach xx] | -0.77076 | 0.029324 |
| DENOEST_v1_3084 | ID:63770308 murC\| UDP-N-acetylmuramate:L-alanine ligase [Denitratisoma oestradiolicum Fahrbach xx] | -0.63007 | 0.029247 |
| DENOEST_v1_3081 | ID:63770305 ftsQ\| Cell division protein FtsQ [Denitratisoma oestradiolicum Fahrbach xx] | -0.78894 | 0.020382 |
| DENOEST_v1_3079 | ID:63770303 ftsZ\| GTP-binding tubulin-like cell division protein [Denitratisoma oestradiolicum Fahrbach xx] | -1.44369 | 0.001027 |
| DENOEST_v1_3078 | ID:63770302 lpxC\| UDP-3-O-acyl N-acetylglucosamine deacetylase [Denitratisoma oestradiolicum Fahrbach xx] | -0.51134 | 0.026396 |
| DENOEST_v1_3075 | ID:63770299 secA\| preprotein translocase subunit, ATPase [Denitratisoma oestradiolicum Fahrbach xx] | -0.77366 | 0.000813 |
| DENOEST_v1_3074 | ID:63770298 mtnA\| Methylthioribose-1-phosphate isomerase [Denitratisoma oestradiolicum Fahrbach xx] | -0.77929 | 0.015278 |
| DENOEST_v1_3073 | ID:63770297 ald\| 5-(methylthio)ribulose-1-phosphate aldolase [Denitratisoma oestradiolicum Fahrbach xx] | -1.06722 | 0.019667 |
| DENOEST_v1_3072 | ID:63770296 Alpha/beta hydrolase fold protein [Denitratisoma oestradiolicum Fahrbach xx] | -0.62757 | 0.005657 |
| DENOEST_v1_3070 | ID:63770294 trpD\| Anthranilate phosphoribosyltransferase [Denitratisoma oestradiolicum Fahrbach xx] | -0.34406 | 0.003234 |
| DENOEST_v1_3069 | ID:63770293 trpG\| bifunctional protein:[Includes: para-aminobenzoate synthase glutamine amidotransferase component II (ADC synthase); anthranilate synthase component II] [Denitratisoma oestradiolicum Fahrbach xx] | -0.56547 | 0.005548 |
| DENOEST_v1_3067 | ID:63770291 argH\| argininosuccinate lyase (Arginosuccinase) (ASAL) [Denitratisoma oestradiolicum Fahrbach xx] | -0.57969 | 0.039679 |
| DENOEST_v1_3052 | ID:63770276 3'-5' exonuclease [Denitratisoma oestradiolicum Fahrbach xx] | 0.652629 | 0.001347 |
| DENOEST_v1_3049 | ID:63770273 Uncharacterized oxidoreductase MexAM1_META1p0182 [Denitratisoma oestradiolicum Fahrbach xx] | 0.639021 | 0.018999 |
| DENOEST_v1_3047 | ID:63770271 Signal transduction protein [Denitratisoma oestradiolicum Fahrbach xx] | 0.8081 | 0.007659 |
| DENOEST_v1_3045 | ID:63770269 waaD\| ADP-L-glycero-D-mannoheptose-6-epimerase, NAD(P)-binding [Denitratisoma oestradiolicum Fahrbach xx] | -0.53243 | 0.000544 |
| DENOEST_v1_3044 | ID:63770268 rfaE\| D-beta-D-heptose 7-phosphate kinase [Denitratisoma oestradiolicum Fahrbach xx] | -0.55865 | 0.005035 |
| DENOEST_v1_3043 | ID:63770267 udg\| UDP-glucose 6-dehydrogenase [Denitratisoma oestradiolicum Fahrbach xx] | -0.59259 | 0.005649 |
| DENOEST_v1_3040 | ID:63770264 ihfB\| integration host factor (IHF), DNA-binding protein, beta subunit [Denitratisoma oestradiolicum Fahrbach xx] | -0.49626 | 0.030845 |
| DENOEST_v1_3039 | ID:63770263 rpsA\| 30S ribosomal protein S1 [Denitratisoma oestradiolicum Fahrbach xx] | -1.30316 | 7.67E-05 |
| DENOEST_v1_3035 | ID:63770259 nirS\| Nitrite reductase [Denitratisoma oestradiolicum Fahrbach xx] | 1.799417 | 0.00054 |
| DENOEST_v1_3029 | ID:63770253 Short-chain dehydrogenase [Denitratisoma oestradiolicum Fahrbach xx] | -0.65495 | 0.018614 |
| DENOEST_v1_3007 | ID:63770231 conserved protein of unknown function [Denitratisoma oestradiolicum Fahrbach xx] | -1.13455 | 0.016734 |
| DENOEST_v1_3002 | ID:63770226 conserved protein of unknown function [Denitratisoma oestradiolicum Fahrbach xx] | 0.373062 | 0.034437 |
| DENOEST_v1_2997 | ID:63770221 himA\| integration host factor (IHF), alpha subunit, DNA-binding protein, DNA replication [Denitratisoma oestradiolicum Fahrbach xx] | -0.4896 | 0.008185 |
| DENOEST_v1_2996 | ID:63770220 pheT\| phenylalanine tRNA synthetase, beta subunit [Denitratisoma oestradiolicum Fahrbach xx] | -0.84241 | 0.001397 |
| DENOEST_v1_2995 | ID:63770219 pheS\| phenylalanine tRNA synthetase, alpha subunit [Denitratisoma oestradiolicum Fahrbach xx] | -0.46395 | 0.003313 |
| DENOEST_v1_2992 | ID:63770216 infC\| Translation initiation factor IF-3 [Denitratisoma oestradiolicum Fahrbach xx] | -0.93849 | 0.001176 |
| DENOEST_v1_2988 | ID:63770212 Cytochrome C oxidase subunit II [Denitratisoma oestradiolicum Fahrbach xx] | 0.879188 | 0.000857 |
| DENOEST_v1_2985 | ID:63770209 Electron transporter SenC [Denitratisoma oestradiolicum Fahrbach xx] | 1.166813 | 0.00075 |
| DENOEST_v1_2983 | ID:63770207 conserved protein of unknown function [Denitratisoma oestradiolicum Fahrbach xx] | 0.442349 | 0.043216 |
| DENOEST_v1_2978 | ID:63770202 Diguanylate cyclase [Denitratisoma oestradiolicum Fahrbach xx] | 0.31267 | 0.011461 |
| DENOEST_v1_2976 | ID:63770200 Response regulator with CheY-like receiver, AAA-type ATPase, and DNA-binding domains [Denitratisoma oestradiolicum Fahrbach xx] | 0.310653 | 0.016061 |
| DENOEST_v1_2975 | ID:63770199 putative Formyl-CoA transferase [Denitratisoma oestradiolicum Fahrbach xx] | 1.881174 | 0.00044 |
| DENOEST_v1_2974 | ID:63770198 putative enzyme [Denitratisoma oestradiolicum Fahrbach xx] | 1.910237 | 0.000658 |
| DENOEST_v1_2973 | ID:63770197 menB\| 1,4-dihydroxy-2-naphthoyl-CoA synthase [Denitratisoma oestradiolicum Fahrbach xx] | 1.725389 | 2.41E-05 |
| DENOEST_v1_2972 | ID:63770196 Acetyl-CoA acetyltransferase [Denitratisoma oestradiolicum Fahrbach xx] | 1.486672 | 0.002602 |
| DENOEST_v1_2971 | ID:63770195 conserved protein of unknown function [Denitratisoma oestradiolicum Fahrbach xx] | 1.618638 | 0.000488 |
| DENOEST_v1_2970 | ID:63770194 Enoyl-CoA hydratase/isomerase [Denitratisoma oestradiolicum Fahrbach xx] | 0.966848 | 0.001259 |
| DENOEST_v1_2969 | ID:63770193 fno\| F420-dependent NADP reductase [Denitratisoma oestradiolicum Fahrbach xx] | 0.739869 | 0.028201 |
| DENOEST_v1_2968 | ID:63770192 Amidohydrolase 2 [Denitratisoma oestradiolicum Fahrbach xx] | 1.287251 | 0.000772 |
| DENOEST_v1_2967 | ID:63770191 L-carnitine dehydratase/bile acid-inducible protein F [Denitratisoma oestradiolicum Fahrbach xx] | 2.451867 | 0.000235 |
| DENOEST_v1_2966 | ID:63770190 Acyl-CoA dehydrogenase domain-containing protein [Denitratisoma oestradiolicum Fahrbach xx] | 3.462032 | 0.000315 |
| DENOEST_v1_2965 | ID:63770189 L-carnitine dehydratase/bile acid-inducible protein F [Denitratisoma oestradiolicum Fahrbach xx] | 4.003986 | 5.28E-06 |
| DENOEST_v1_2964 | ID:63770188 conserved protein of unknown function [Denitratisoma oestradiolicum Fahrbach xx] | 3.549738 | 0.000962 |
| DENOEST_v1_2963 | ID:63770187 MaoC domain protein dehydratase [Denitratisoma oestradiolicum Fahrbach xx] | 3.502679 | 5.25E-05 |
| DENOEST_v1_2962 | ID:63770186 Acyl-CoA dehydrogenase domain-containing protein [Denitratisoma oestradiolicum Fahrbach xx] | 3.106022 | 1.86E-05 |
| DENOEST_v1_2961 | ID:63770185 Putative Succinyl-CoA:(R)-benzylsuccinate CoA-transferase subunit BbsF (modular protein) [Denitratisoma oestradiolicum Fahrbach xx] | 1.792188 | 0.003154 |
| DENOEST_v1_2960 | ID:63770184 putative Succinyl-CoA:(R)-benzylsuccinate CoA-transferase subunit BbsF [Denitratisoma oestradiolicum Fahrbach xx] | 2.195787 | 0.002169 |
| DENOEST_v1_2958 | ID:63770182 putative 1,2-epoxyphenylacetyl-CoA isomerase [Denitratisoma oestradiolicum Fahrbach xx] | 1.69425 | 0.000503 |
| DENOEST_v1_2956 | ID:63770180 capA\| Capsule biosynthesis protein CapA [Denitratisoma oestradiolicum Fahrbach xx] | 2.791967 | 0.000244 |
| DENOEST_v1_2955 | ID:63770179 nirS\| Nitrite reductase [Denitratisoma oestradiolicum Fahrbach xx] | 0.142457 | 0.026063 |
| DENOEST_v1_2927 | ID:63770151 conserved hypothetical protein; putative signal peptide [Denitratisoma oestradiolicum Fahrbach xx] | -0.35228 | 0.035155 |
| DENOEST_v1_2922 | ID:63770146 radA\| repair protein radA homolog [Denitratisoma oestradiolicum Fahrbach xx] | -0.23677 | 0.032651 |
| DENOEST_v1_2920 | ID:63770144 conserved exported protein of unknown function [Denitratisoma oestradiolicum Fahrbach xx] | 0.82089 | 0.038959 |
| DENOEST_v1_2919 | ID:63770143 alaS\| alanyl-tRNA synthetase [Denitratisoma oestradiolicum Fahrbach xx] | -0.43345 | 0.007567 |
| DENOEST_v1_2918 | ID:63770142 conserved protein of unknown function [Denitratisoma oestradiolicum Fahrbach xx] | -0.41749 | 0.009361 |
| DENOEST_v1_2913 | ID:63770137 rfaF\| ADP-heptose--LPS heptosyltransferase 2 [Denitratisoma oestradiolicum Fahrbach xx] | -0.56051 | 0.005767 |
| DENOEST_v1_2912 | ID:63770136 conserved protein of unknown function [Denitratisoma oestradiolicum Fahrbach xx] | -1.44869 | 0.007567 |
| DENOEST_v1_2910 | ID:63770134 conserved protein of unknown function [Denitratisoma oestradiolicum Fahrbach xx] | 0.419447 | 0.003287 |
| DENOEST_v1_2892 | ID:63770116 nudJ\| Phosphatase NudJ [Denitratisoma oestradiolicum Fahrbach xx] | -0.59777 | 0.006099 |
| DENOEST_v1_2890 | ID:63770114 trmU\| tRNA (5-methylaminomethyl-2-thiouridylate)-methyltransferase [Denitratisoma oestradiolicum Fahrbach xx] | -0.38625 | 0.013373 |
| DENOEST_v1_2888 | ID:63770112 yibF\| Uncharacterized GST-like protein YibF [Denitratisoma oestradiolicum Fahrbach xx] | -1.11446 | 0.001524 |
| DENOEST_v1_2886 | ID:63770110 purB\| adenylosuccinate lyase [Denitratisoma oestradiolicum Fahrbach xx] | -0.88573 | 0.00892 |
| DENOEST_v1_2882 | ID:63770106 secF\| SecYEG protein translocase auxillary subunit [Denitratisoma oestradiolicum Fahrbach xx] | 0.673618 | 0.004498 |
| DENOEST_v1_2881 | ID:63770105 secD\| SecYEG protein translocase auxillary subunit [Denitratisoma oestradiolicum Fahrbach xx] | 2.221997 | 0.00019 |
| DENOEST_v1_2879 | ID:63770103 yajC\| SecYEG protein translocase auxillary subunit [Denitratisoma oestradiolicum Fahrbach xx] | -0.70312 | 0.010334 |
| DENOEST_v1_2871 | ID:63770095 Cytochrome c5 [Denitratisoma oestradiolicum Fahrbach xx] | -2.09729 | 0.005452 |
| DENOEST_v1_2870 | ID:63770094 dhmA\| Haloalkane dehalogenase [Denitratisoma oestradiolicum Fahrbach xx] | -0.53951 | 0.001586 |
| DENOEST_v1_2866 | ID:63770090 minC\| putative septum site-determining protein MinC [Denitratisoma oestradiolicum Fahrbach xx] | 0.879 | 0.018034 |
| DENOEST_v1_2865 | ID:63770089 minD\| cell division inhibitor, a membrane ATPase,activates minC [Denitratisoma oestradiolicum Fahrbach xx] | -0.36417 | 0.003594 |
| DENOEST_v1_2864 | ID:63770088 minE\| cell division topological specificity factor [Denitratisoma oestradiolicum Fahrbach xx] | -0.69389 | 0.000426 |
| DENOEST_v1_2863 | ID:63770087 hemC\| hydroxymethylbilane synthase [Denitratisoma oestradiolicum Fahrbach xx] | -0.49996 | 0.003268 |
| DENOEST_v1_2861 | ID:63770085 conserved protein of unknown function [Denitratisoma oestradiolicum Fahrbach xx] | -0.26778 | 0.003897 |
| DENOEST_v1_2859 | ID:63770083 hemF\| coproporphyrinogen III oxidase [Denitratisoma oestradiolicum Fahrbach xx] | -0.33101 | 0.009605 |
| DENOEST_v1_2857 | ID:63770081 purD\| phosphoribosylamine--glycine ligase (GARS) (Glycinamide ribonucleotide synthetase) (Phosphoribosylglycinamide synthetase) [Denitratisoma oestradiolicum Fahrbach xx] | -0.33514 | 0.040417 |
| DENOEST_v1_2856 | ID:63770080 purH\| bifunctional protein [Includes: phosphoribosylaminoimidazolecarboxamide formyltransferase (AICAR transformylase); IMP cyclohydrolase (Inosinicase) (IMP synthetase) (ATIC)] [Denitratisoma oestradiolicum Fahrbach xx] | -0.50789 | 0.00405 |
| DENOEST_v1_2850 | ID:63770074 RidA family protein [Denitratisoma oestradiolicum Fahrbach xx] | 0.805632 | 0.033118 |
| DENOEST_v1_2848 | ID:63770072 pepP\| proline aminopeptidase P II [Denitratisoma oestradiolicum Fahrbach xx] | -0.31758 | 0.000191 |
| DENOEST_v1_2844 | ID:63770068 lptD\| LPS-assembly protein LptD [Denitratisoma oestradiolicum Fahrbach xx] | -0.57403 | 0.006493 |
| DENOEST_v1_2842 | ID:63770066 pdxA\| 4-hydroxy-L-threonine phosphate dehydrogenase, NAD-dependent [Denitratisoma oestradiolicum Fahrbach xx] | -0.41559 | 0.00575 |
| DENOEST_v1_2833 | ID:63770057 Serine/threonine protein phosphatase [Denitratisoma oestradiolicum Fahrbach xx] | 0.337744 | 0.027873 |
| DENOEST_v1_2829 | ID:63770053 rhlE\| RNA helicase [Denitratisoma oestradiolicum Fahrbach xx] | -1.47738 | 5.24E-05 |
| DENOEST_v1_2823 | ID:63770047 qseB\| DNA-binding response regulator in two-component regulatory system with QseC [Denitratisoma oestradiolicum Fahrbach xx] | 0.67817 | 0.003131 |
| DENOEST_v1_2821 | ID:63770045 conserved protein of unknown function [Denitratisoma oestradiolicum Fahrbach xx] | 0.745885 | 0.009339 |
| DENOEST_v1_2820 | ID:63770044 Cyclic nucleotide-binding protein [Denitratisoma oestradiolicum Fahrbach xx] | -0.79953 | 0.001801 |
| DENOEST_v1_2817 | ID:63770041 Cytochrome P450 [Denitratisoma oestradiolicum Fahrbach xx] | 0.82354 | 0.004239 |
| DENOEST_v1_2815 | ID:63770039 Radical SAM protein [Denitratisoma oestradiolicum Fahrbach xx] | -0.41804 | 0.000768 |
| DENOEST_v1_2814 | ID:63770038 Translation elongation factor 2 (EF-2/EF-G) [Denitratisoma oestradiolicum Fahrbach xx] | 0.196752 | 0.007655 |
| DENOEST_v1_2811 | ID:63770035 ubiG\| 3-demethylubiquinone-9 3-methyltransferase and 2-octaprenyl-6-hydroxy phenol methylase [Denitratisoma oestradiolicum Fahrbach xx] | -0.369 | 0.001639 |
| DENOEST_v1_2809 | ID:63770033 mtaD\| 5-methylthioadenosine/S-adenosylhomocysteine deaminase [Denitratisoma oestradiolicum Fahrbach xx] | 0.676857 | 0.014014 |
| DENOEST_v1_2808 | ID:63770032 gyrA\| DNA gyrase (type II topoisomerase), subunit A [Denitratisoma oestradiolicum Fahrbach xx] | -0.44387 | 0.000546 |
| DENOEST_v1_2807 | ID:63770031 serC\| 3-phosphoserine/phosphohydroxythreonine aminotransferase [Denitratisoma oestradiolicum Fahrbach xx] | -0.2647 | 0.025692 |
| DENOEST_v1_2806 | ID:63770030 Phosphoglycerate dehydrogenase-like oxidoreductase [Denitratisoma oestradiolicum Fahrbach xx] | -0.44116 | 0.004774 |
| DENOEST_v1_2805 | ID:63770029 pheA\| Chorismate mutase / Prephenate dehydratase [Denitratisoma oestradiolicum Fahrbach xx] | 1.306586 | 0.013709 |
| DENOEST_v1_2803 | ID:63770027 Prephenate dehydrogenase [Denitratisoma oestradiolicum Fahrbach xx] | -0.5524 | 0.000716 |
| DENOEST_v1_2795 | ID:63770019 accA\| acetyl-CoA carboxylase, carboxytransferase, alpha subunit [Denitratisoma oestradiolicum Fahrbach xx] | -1.05999 | 0.000628 |
| DENOEST_v1_2793 | ID:63770017 ndk\| nucleoside diphosphate kinase (NDK) (NDP kinase) (Nucleoside-2-P kinase) [Denitratisoma oestradiolicum Fahrbach xx] | -1.50056 | 0.000969 |
| DENOEST_v1_2792 | ID:63770016 yfgB\| putative Fe-S containing enzyme [Denitratisoma oestradiolicum Fahrbach xx] | -1.37291 | 0.019202 |
| DENOEST_v1_2789 | ID:63770013 ispG\| 4-hydroxy-3-methylbut-2-en-1-yl diphosphate synthase (flavodoxin) [Denitratisoma oestradiolicum Fahrbach xx] | -0.70931 | 0.000319 |
| DENOEST_v1_2787 | ID:63770011 conserved protein of unknown function [Denitratisoma oestradiolicum Fahrbach xx] | -0.75189 | 0.003352 |
| DENOEST_v1_2786 | ID:63770010 bamB\| Outer membrane protein assembly factor BamB [Denitratisoma oestradiolicum Fahrbach xx] | -0.62939 | 0.000895 |
| DENOEST_v1_2785 | ID:63770009 der\| GTPase involved in ribosome synthesis and maintenance [Denitratisoma oestradiolicum Fahrbach xx] | -0.88812 | 0.000172 |
| DENOEST_v1_2782 | ID:63770006 hflK\| Protein HflK [Denitratisoma oestradiolicum Fahrbach xx] | -0.61923 | 0.016803 |
| DENOEST_v1_2781 | ID:63770005 hflC\| Protein HflC [Denitratisoma oestradiolicum Fahrbach xx] | -0.65654 | 0.001611 |
| DENOEST_v1_2779 | ID:63770003 hisZ\| ATP phosphoribosyltransferase regulatory subunit [Denitratisoma oestradiolicum Fahrbach xx] | -0.80567 | 0.000668 |
| DENOEST_v1_2778 | ID:63770002 purA\| adenylosuccinate synthetase [Denitratisoma oestradiolicum Fahrbach xx] | -0.97185 | 0.000534 |
| DENOEST_v1_2760 | ID:63769984 Molybdopterin-binding protein [Denitratisoma oestradiolicum Fahrbach xx] | 4.434875 | 7.14E-06 |
| DENOEST_v1_2759 | ID:63769983 Granule-associated-like protein [Denitratisoma oestradiolicum Fahrbach xx] | -1.77055 | 0.020509 |
| DENOEST_v1_2756 | ID:63769980 kdsC\| 3-deoxy-D-manno-octulosonate 8-phosphate phosphatase [Denitratisoma oestradiolicum Fahrbach xx] | -2.4354 | 6.13E-05 |
| DENOEST_v1_2755 | ID:63769979 kdsD\| D-arabinose 5-phosphate isomerase [Denitratisoma oestradiolicum Fahrbach xx] | -0.38913 | 0.037609 |
| DENOEST_v1_2735 | ID:63769959 conserved protein of unknown function [Denitratisoma oestradiolicum Fahrbach xx] | 0.78003 | 0.014451 |
| DENOEST_v1_2726 | ID:63769950 conserved protein of unknown function [Denitratisoma oestradiolicum Fahrbach xx] | -0.47521 | 0.016995 |
| DENOEST_v1_2724 | ID:63769948 Transketolase [Denitratisoma oestradiolicum Fahrbach xx] | 0.564446 | 0.034899 |
| DENOEST_v1_2721 | ID:63769945 putative TonB-dependent receptor [Denitratisoma oestradiolicum Fahrbach xx] | 0.811157 | 0.002061 |
| DENOEST_v1_2714 | ID:63769938 putative TonB-dependent receptor [Denitratisoma oestradiolicum Fahrbach xx] | 2.093384 | 0.003138 |
| DENOEST_v1_2708 | ID:63769932 pdhB\| Pyruvate dehydrogenase E1 component, beta subunit [Denitratisoma oestradiolicum Fahrbach xx] | 19.00701 | 0.006188 |
| DENOEST_v1_2701 | ID:63769925 putative TonB-dependent receptor [Denitratisoma oestradiolicum Fahrbach xx] | 1.234466 | 2.39E-05 |
| DENOEST_v1_2666 | ID:63769890 ppa\| inorganic pyrophosphatase [Denitratisoma oestradiolicum Fahrbach xx] | -0.90329 | 0.006323 |
| DENOEST_v1_2664 | ID:63769888 nadE\| Glutamine-dependent NAD(+) synthetase [Denitratisoma oestradiolicum Fahrbach xx] | -1.3318 | 0.000894 |
| DENOEST_v1_2663 | ID:63769887 glnK\| regulatory protein, P-II 2, for nitrogen assimilation by glutamine synthetase, regulates GlnL (NRII) and GlnE (ATase) [Denitratisoma oestradiolicum Fahrbach xx] | -1.38074 | 9.67E-06 |
| DENOEST_v1_2658 | ID:63769882 lolA\| Outer-membrane lipoprotein carrier protein [Denitratisoma oestradiolicum Fahrbach xx] | -0.25113 | 0.040981 |
| DENOEST_v1_2657 | ID:63769881 cspE\| DNA-binding transcriptional repressor [Denitratisoma oestradiolicum Fahrbach xx] | -2.21792 | 0.001726 |
| DENOEST_v1_2655 | ID:63769879 infA\| translation initiation factor IF-1 [Denitratisoma oestradiolicum Fahrbach xx] | -1.94205 | 0.009648 |
| DENOEST_v1_2652 | ID:63769876 Flavin oxidoreductase [Denitratisoma oestradiolicum Fahrbach xx] | 2.14089 | 0.002483 |
| DENOEST_v1_2650 | ID:63769874 TonB-dependent receptor [Denitratisoma oestradiolicum Fahrbach xx] | -25.0308 | 3.75E-07 |
| DENOEST_v1_2649 | ID:63769873 Glycosyl hydrolase [Denitratisoma oestradiolicum Fahrbach xx] | -4.10091 | 0.000122 |
| DENOEST_v1_2648 | ID:63769872 conserved protein of unknown function [Denitratisoma oestradiolicum Fahrbach xx] | -18.2372 | 0.001116 |
| DENOEST_v1_2646 | ID:63769870 TonB-dependent receptor [Denitratisoma oestradiolicum Fahrbach xx] | -22.6733 | 9.88E-05 |
| DENOEST_v1_2645 | ID:63769869 2-(1,2-epoxy-1,2-dihydrophenyl)acetyl-CoA isomerase [Denitratisoma oestradiolicum Fahrbach xx] | -0.34561 | 0.049911 |
| DENOEST_v1_2643 | ID:63769867 linB\| 2,5-dichloro-2,5-cyclohexadiene-1,4-diol dehydrogenase [Denitratisoma oestradiolicum Fahrbach xx] | 1.00805 | 0.014089 |
| DENOEST_v1_2641 | ID:63769865 regA\| Photosynthetic apparatus regulatory protein RegA [Denitratisoma oestradiolicum Fahrbach xx] | -1.28922 | 0.000849 |
| DENOEST_v1_2639 | ID:63769863 conserved exported protein of unknown function [Denitratisoma oestradiolicum Fahrbach xx] | -1.26114 | 0.006852 |
| DENOEST_v1_2638 | ID:63769862 conserved protein of unknown function [Denitratisoma oestradiolicum Fahrbach xx] | -1.00012 | 0.011466 |
| DENOEST_v1_2634 | ID:63769858 lysA\| Diaminopimelate decarboxylase [Denitratisoma oestradiolicum Fahrbach xx] | -0.16218 | 0.030326 |
| DENOEST_v1_2620 | ID:63769844 glmU\| fused N-acetyl glucosamine-1-phosphate uridyltransferase ; glucosamine-1-phosphate acetyl transferase [Denitratisoma oestradiolicum Fahrbach xx] | -0.51002 | 0.001021 |
| DENOEST_v1_2609 | ID:63769833 hisC\| Histidinol-phosphate aminotransferase [Denitratisoma oestradiolicum Fahrbach xx] | -0.28468 | 0.006051 |
| DENOEST_v1_2606 | ID:63769830 hisA\| phosphoribosylformimino-5-aminoimidazole carboxamide isomerase [Denitratisoma oestradiolicum Fahrbach xx] | -0.78928 | 0.022068 |
| DENOEST_v1_2605 | ID:63769829 hisF\| imidazole glycerol phosphate synthase, catalytic subunit with HisH [Denitratisoma oestradiolicum Fahrbach xx] | -0.68346 | 0.019537 |
| DENOEST_v1_2604 | ID:63769828 hisIE\| fragment of bifunctional protein [Includes: phosphoribosyl-AMP cyclohydrolase; phosphoribosyl-ATP pyrophosphatase] (part 1) [Denitratisoma oestradiolicum Fahrbach xx] | -0.11309 | 0.017934 |
| DENOEST_v1_2601 | ID:63769825 tatA\| TatABCE protein translocation system subunit [Denitratisoma oestradiolicum Fahrbach xx] | -1.59588 | 0.001301 |
| DENOEST_v1_2597 | ID:63769821 GTP cyclohydrolase 1 type 2 homolog [Denitratisoma oestradiolicum Fahrbach xx] | 0.862136 | 0.001245 |
| DENOEST_v1_2593 | ID:63769817 General secretion pathway protein GspD [Denitratisoma oestradiolicum Fahrbach xx] | -1.30349 | 0.001222 |
| DENOEST_v1_2588 | ID:63769812 xcpR\| Type II secretion system protein E [Denitratisoma oestradiolicum Fahrbach xx] | 0.608017 | 0.000394 |
| DENOEST_v1_2584 | ID:63769808 conserved protein of unknown function [Denitratisoma oestradiolicum Fahrbach xx] | -1.01232 | 0.037319 |
| DENOEST_v1_2583 | ID:63769807 conserved exported protein of unknown function [Denitratisoma oestradiolicum Fahrbach xx] | -0.94704 | 0.003833 |
| DENOEST_v1_2582 | ID:63769806 exported protein of unknown function [Denitratisoma oestradiolicum Fahrbach xx] | -0.91658 | 0.000227 |
| DENOEST_v1_2573 | ID:63769797 putative enzyme [Denitratisoma oestradiolicum Fahrbach xx] | 1.029273 | 0.039108 |
| DENOEST_v1_2567 | ID:63769791 Acyl-CoA dehydrogenase domain protein [Denitratisoma oestradiolicum Fahrbach xx] | 0.759342 | 0.008987 |
| DENOEST_v1_2566 | ID:63769790 conserved protein of unknown function [Denitratisoma oestradiolicum Fahrbach xx] | -0.47324 | 0.002743 |
| DENOEST_v1_2564 | ID:63769788 AMP-dependent synthetase and ligase [Denitratisoma oestradiolicum Fahrbach xx] | 0.374996 | 0.01253 |
| DENOEST_v1_2561 | ID:63769785 nodQ\| Sulfate adenylyltransferase subunit 1 / Adenylyl-sulfate kinase [Denitratisoma oestradiolicum Fahrbach xx] | -1.79087 | 3.01E-05 |
| DENOEST_v1_2560 | ID:63769784 cysD\| Sulfate adenylyltransferase subunit 2 [Denitratisoma oestradiolicum Fahrbach xx] | -1.89225 | 1.6E-05 |
| DENOEST_v1_2559 | ID:63769783 cysQ\| 3'(2'),5'-bisphosphate nucleotidase CysQ [Denitratisoma oestradiolicum Fahrbach xx] | -1.7022 | 0.000997 |
| DENOEST_v1_2548 | ID:63769772 putative enzyme [Denitratisoma oestradiolicum Fahrbach xx] | 3.668511 | 0.002127 |
| DENOEST_v1_2546 | ID:63769770 Acyl-CoA dehydrogenase domain-containing protein [Denitratisoma oestradiolicum Fahrbach xx] | 1.057843 | 0.036243 |
| DENOEST_v1_2545 | ID:63769769 L-sorbose 1-dehydrogenase [Denitratisoma oestradiolicum Fahrbach xx] | 0.863675 | 0.019917 |
| DENOEST_v1_2542 | ID:63769766 Aminotransferase [Denitratisoma oestradiolicum Fahrbach xx] | 0.968034 | 0.000217 |
| DENOEST_v1_2537 | ID:63769761 conserved exported protein of unknown function [Denitratisoma oestradiolicum Fahrbach xx] | 1.279329 | 0.004885 |
| DENOEST_v1_2536 | ID:63769760 conserved exported protein of unknown function [Denitratisoma oestradiolicum Fahrbach xx] | 0.903906 | 0.001799 |
| DENOEST_v1_2535 | ID:63769759 Sulfatase [Denitratisoma oestradiolicum Fahrbach xx] | 0.458479 | 0.000641 |
| DENOEST_v1_2534 | ID:63769758 Arylsulfatase [Denitratisoma oestradiolicum Fahrbach xx] | 0.497387 | 0.030662 |
| DENOEST_v1_2533 | ID:63769757 putative TonB-dependent receptor [Denitratisoma oestradiolicum Fahrbach xx] | 20.26036 | 0.010965 |
| DENOEST_v1_2532 | ID:63769756 Flavin-dependent oxidoreductase, luciferase family (Includes alkanesulfonate monooxygenase SsuD and methylene tetrahydromethanopterin reductase) [Denitratisoma oestradiolicum Fahrbach xx] | 0.268491 | 0.048931 |
| DENOEST_v1_2531 | ID:63769755 Helix-turn-helix protein, CopG [Denitratisoma oestradiolicum Fahrbach xx] | -0.39524 | 0.016748 |
| DENOEST_v1_2530 | ID:63769754 metY\| homocysteine synthase [Denitratisoma oestradiolicum Fahrbach xx] | -0.23178 | 0.002438 |
| DENOEST_v1_2528 | ID:63769752 yhfP\| putative quinone oxidoreductase YhfP [Denitratisoma oestradiolicum Fahrbach xx] | -1.8672 | 1.57E-05 |
| DENOEST_v1_2527 | ID:63769751 prpE\| propionyl-CoA synthetase [Denitratisoma oestradiolicum Fahrbach xx] | -2.86258 | 9.18E-07 |
| DENOEST_v1_2526 | ID:63769750 rpsP\| 30S ribosomal protein S16 [Denitratisoma oestradiolicum Fahrbach xx] | -1.31263 | 0.000461 |
| DENOEST_v1_2525 | ID:63769749 rimM\| Ribosome maturation factor RimM [Denitratisoma oestradiolicum Fahrbach xx] | -2.51012 | 0.002443 |
| DENOEST_v1_2523 | ID:63769747 rplS\| 50S ribosomal protein L19 [Denitratisoma oestradiolicum Fahrbach xx] | -0.97359 | 0.000113 |
| DENOEST_v1_2522 | ID:63769746 conserved exported protein of unknown function [Denitratisoma oestradiolicum Fahrbach xx] | 0.229735 | 0.033698 |
| DENOEST_v1_2521 | ID:63769745 putative Methyl-accepting chemotaxis sensory transducer [Denitratisoma oestradiolicum Fahrbach xx] | 1.237118 | 1.89E-05 |
| DENOEST_v1_2520 | ID:63769744 conserved protein of unknown function [Denitratisoma oestradiolicum Fahrbach xx] | 1.210174 | 0.005423 |
| DENOEST_v1_2517 | ID:63769741 dnaB\| replicative DNA helicase [Denitratisoma oestradiolicum Fahrbach xx] | -0.97384 | 0.042483 |
| DENOEST_v1_2516 | ID:63769740 Phosphate starvation-inducible protein PhoH [Denitratisoma oestradiolicum Fahrbach xx] | 0.543735 | 0.008087 |
| DENOEST_v1_2515 | ID:63769739 conserved exported protein of unknown function [Denitratisoma oestradiolicum Fahrbach xx] | 4.653142 | 2.32E-05 |
| DENOEST_v1_2513 | ID:63769737 cobO\| Cob(I)yrinic acid a,c-diamide adenosyltransferase [Denitratisoma oestradiolicum Fahrbach xx] | 2.456268 | 0.000393 |
| DENOEST_v1_2512 | ID:63769736 cobB\| Hydrogenobyrinate a,c-diamide synthase [Denitratisoma oestradiolicum Fahrbach xx] | 3.809267 | 6.14E-06 |
| DENOEST_v1_2511 | ID:63769735 bluB\| 5,6-dimethylbenzimidazole synthase [Denitratisoma oestradiolicum Fahrbach xx] | 3.205896 | 8.88E-05 |
| DENOEST_v1_2505 | ID:63769729 lysC\| aspartate kinase [Denitratisoma oestradiolicum Fahrbach xx] | -0.42935 | 0.027817 |
| DENOEST_v1_2503 | ID:63769727 fumC\| fumarate hydratase (fumarase C),aerobic Class II [Denitratisoma oestradiolicum Fahrbach xx] | 1.108763 | 0.032304 |
| DENOEST_v1_2502 | ID:63769726 mls\| Malate synthase [Denitratisoma oestradiolicum Fahrbach xx] | -4.02569 | 1.53E-05 |
| DENOEST_v1_2497 | ID:63769721 K348 [Denitratisoma oestradiolicum Fahrbach xx] | -0.6714 | 0.002663 |
| DENOEST_v1_2495 | ID:63769719 ccoP\| Cbb3-type cytochrome c oxidase subunit CcoP2 [Denitratisoma oestradiolicum Fahrbach xx] | -0.37021 | 0.01452 |
| DENOEST_v1_2492 | ID:63769716 conserved protein of unknown function [Denitratisoma oestradiolicum Fahrbach xx] | -0.32314 | 0.015794 |
| DENOEST_v1_2491 | ID:63769715 phaC\| Poly(3-hydroxyalkanoate) polymerase subunit PhaC [Denitratisoma oestradiolicum Fahrbach xx] | 1.25091 | 0.01807 |
| DENOEST_v1_2490 | ID:63769714 2-nitropropane dioxygenase [Denitratisoma oestradiolicum Fahrbach xx] | -0.48085 | 0.000899 |
| DENOEST_v1_2476 | ID:63769700 Porin [Denitratisoma oestradiolicum Fahrbach xx] | -22.8546 | 0.006644 |
| DENOEST_v1_2475 | ID:63769699 conserved exported protein of unknown function [Denitratisoma oestradiolicum Fahrbach xx] | -5.63448 | 0.003336 |
| DENOEST_v1_2474 | ID:63769698 bfr\| bacterioferritin, iron storage and detoxification protein [Denitratisoma oestradiolicum Fahrbach xx] | -0.77818 | 0.000562 |
| DENOEST_v1_2468 | ID:63769692 Ketosteroid isomerase-related protein [Denitratisoma oestradiolicum Fahrbach xx] | 0.523525 | 0.02205 |
| DENOEST_v1_2466 | ID:63769690 conserved membrane protein of unknown function [Denitratisoma oestradiolicum Fahrbach xx] | -2.51995 | 0.028198 |
| DENOEST_v1_2465 | ID:63769689 D-arabinose 1-dehydrogenase, Zn-dependent alcohol dehydrogenase family [Denitratisoma oestradiolicum Fahrbach xx] | -2.02391 | 0.00012 |
| DENOEST_v1_2464 | ID:63769688 conserved protein of unknown function [Denitratisoma oestradiolicum Fahrbach xx] | -2.75748 | 1.63E-05 |
| DENOEST_v1_2460 | ID:63769684 hslU\| molecular chaperone and ATPase component of HslUV protease [Denitratisoma oestradiolicum Fahrbach xx] | -0.36656 | 0.00286 |
| DENOEST_v1_2459 | ID:63769683 hslV\| peptidase component of the HslUV protease [Denitratisoma oestradiolicum Fahrbach xx] | -0.42725 | 0.002357 |
| DENOEST_v1_2441 | ID:63769665 FAD-linked oxidase [Denitratisoma oestradiolicum Fahrbach xx] | 0.642056 | 0.025493 |
| DENOEST_v1_2438 | ID:63769662 5-oxoprolinase [Denitratisoma oestradiolicum Fahrbach xx] | 0.468038 | 0.024008 |
| DENOEST_v1_2436 | ID:63769660 Amidohydrolase [Denitratisoma oestradiolicum Fahrbach xx] | -0.67134 | 0.002345 |
| DENOEST_v1_2434 | ID:63769658 conserved protein of unknown function [Denitratisoma oestradiolicum Fahrbach xx] | -0.76334 | 0.026905 |
| DENOEST_v1_2430 | ID:63769654 conserved protein of unknown function [Denitratisoma oestradiolicum Fahrbach xx] | 0.767053 | 0.015874 |
| DENOEST_v1_2420 | ID:63769644 conserved protein of unknown function [Denitratisoma oestradiolicum Fahrbach xx] | -0.36504 | 0.044123 |
| DENOEST_v1_2408 | ID:63769632 conserved exported protein of unknown function [Denitratisoma oestradiolicum Fahrbach xx] | -0.44063 | 0.012076 |
| DENOEST_v1_2398 | ID:63769622 putative TonB-dependent receptor [Denitratisoma oestradiolicum Fahrbach xx] | 0.784873 | 0.000489 |
| DENOEST_v1_2396 | ID:63769620 Disulfide bond formation protein DsbA [Denitratisoma oestradiolicum Fahrbach xx] | -3.58462 | 5.2E-05 |
| DENOEST_v1_2383 | ID:63769607 5-oxoprolinase [Denitratisoma oestradiolicum Fahrbach xx] | 0.682274 | 0.001426 |
| DENOEST_v1_2378 | ID:63769602 conserved protein of unknown function [Denitratisoma oestradiolicum Fahrbach xx] | 0.564291 | 0.005233 |
| DENOEST_v1_2368 | ID:63769592 nadC\| quinolinate phosphoribosyltransferase [Denitratisoma oestradiolicum Fahrbach xx] | 0.321988 | 0.040156 |
| DENOEST_v1_2362 | ID:63769586 conserved protein of unknown function [Denitratisoma oestradiolicum Fahrbach xx] | -2.93449 | 0.008535 |
| DENOEST_v1_2356 | ID:63769580 recQ\| ATP-dependent DNA helicase RecQ [Denitratisoma oestradiolicum Fahrbach xx] | -1.79264 | 0.000168 |
| DENOEST_v1_2354 | ID:63769578 ppiB\| peptidyl-prolyl cis-trans isomerase B (rotamase B) [Denitratisoma oestradiolicum Fahrbach xx] | -1.12807 | 0.003635 |
| DENOEST_v1_2353 | ID:63769577 ppiB\| peptidyl-prolyl cis-trans isomerase precursor (PPIase) (Rotamase) [Denitratisoma oestradiolicum Fahrbach xx] | -0.67183 | 0.000508 |
| DENOEST_v1_2350 | ID:63769574 cysS\| cysteinyl-tRNA synthetase [Denitratisoma oestradiolicum Fahrbach xx] | -0.55168 | 0.000481 |
| DENOEST_v1_2349 | ID:63769573 conserved protein of unknown function [Denitratisoma oestradiolicum Fahrbach xx] | -0.72217 | 0.000728 |
| DENOEST_v1_2348 | ID:63769572 dnaJ\| chaperone Hsp40, co-chaperone with DnaK [Denitratisoma oestradiolicum Fahrbach xx] | 1.690819 | 7.63E-05 |
| DENOEST_v1_2343 | ID:63769567 hrcA\| Heat-inducible transcription repressor HrcA [Denitratisoma oestradiolicum Fahrbach xx] | -0.87581 | 9.57E-06 |
| DENOEST_v1_2338 | ID:63769562 dapB\| dihydrodipicolinate reductase [Denitratisoma oestradiolicum Fahrbach xx] | -0.25369 | 0.033613 |
| DENOEST_v1_2337 | ID:63769561 carA\| carbamoyl-phosphate synthase, small chain [Denitratisoma oestradiolicum Fahrbach xx] | -0.59107 | 0.011188 |
| DENOEST_v1_2336 | ID:63769560 carB\| carbamoyl-phosphate synthase, large subunit [Denitratisoma oestradiolicum Fahrbach xx] | -0.56808 | 9.16E-05 |
| DENOEST_v1_2335 | ID:63769559 greA\| transcription elongation factor [Denitratisoma oestradiolicum Fahrbach xx] | -0.88949 | 0.001285 |
| DENOEST_v1_2332 | ID:63769556 rrmJ\| 23S rRNA methyltransferase [Denitratisoma oestradiolicum Fahrbach xx] | -1.22201 | 0.002219 |
| DENOEST_v1_2331 | ID:63769555 ftsH\| cell division protein [Denitratisoma oestradiolicum Fahrbach xx] | -0.46759 | 0.00368 |
| DENOEST_v1_2330 | ID:63769554 folP\| 7,8-dihydropteroate synthase [Denitratisoma oestradiolicum Fahrbach xx] | -0.36648 | 0.046484 |
| DENOEST_v1_2328 | ID:63769552 conserved protein of unknown function [Denitratisoma oestradiolicum Fahrbach xx] | -0.22817 | 0.010657 |
| DENOEST_v1_2317 | ID:63769541 pyrG\| CTP synthetase [Denitratisoma oestradiolicum Fahrbach xx] | -0.68419 | 0.000564 |
| DENOEST_v1_2316 | ID:63769540 kdsA\| 2-dehydro-3-deoxyphosphooctonate aldolase [Denitratisoma oestradiolicum Fahrbach xx] | -0.4922 | 0.017189 |
| DENOEST_v1_2314 | ID:63769538 eno\| enolase [Denitratisoma oestradiolicum Fahrbach xx] | -0.91469 | 0.001137 |
| DENOEST_v1_2312 | ID:63769536 cph1\| Phytochrome-like protein cph1 [Denitratisoma oestradiolicum Fahrbach xx] | -0.58833 | 0.012534 |
| DENOEST_v1_2310 | ID:63769534 cph2\| Phytochrome-like protein cph2 [Denitratisoma oestradiolicum Fahrbach xx] | -2.22281 | 0.007008 |
| DENOEST_v1_2307 | ID:63769531 conserved exported protein of unknown function [Denitratisoma oestradiolicum Fahrbach xx] | 1.37792 | 3.69E-05 |
| DENOEST_v1_2304 | ID:63769528 prmB\| 50S ribosomal protein L3 glutamine methyltransferase [Denitratisoma oestradiolicum Fahrbach xx] | -0.76149 | 0.001582 |
| DENOEST_v1_2302 | ID:63769526 Type IV pili twitching motility protein PilT [Denitratisoma oestradiolicum Fahrbach xx] | 0.283962 | 0.038685 |
| DENOEST_v1_2301 | ID:63769525 dapD\| 2,3,4,5-tetrahydropyridine-2-carboxylate N-succinyltransferase [Denitratisoma oestradiolicum Fahrbach xx] | -0.35811 | 0.019325 |
| DENOEST_v1_2296 | ID:63769520 dctP\| C4-dicarboxylate-binding periplasmic protein DctP [Denitratisoma oestradiolicum Fahrbach xx] | 1.313509 | 0.001676 |
| DENOEST_v1_2292 | ID:63769516 Diguanylate cyclase (GGDEF) domain-containing protein [Denitratisoma oestradiolicum Fahrbach xx] | 0.592221 | 0.000934 |
| DENOEST_v1_2291 | ID:63769515 cheB\| Chemotaxis response regulator protein-glutamate methylesterase 4 [Denitratisoma oestradiolicum Fahrbach xx] | 1.290994 | 0.001035 |
| DENOEST_v1_2289 | ID:63769513 Hybrid sensor histidine kinase/response regulator [Denitratisoma oestradiolicum Fahrbach xx] | 0.705428 | 0.032253 |
| DENOEST_v1_2288 | ID:63769512 Chemotaxis protein (modular protein) [Denitratisoma oestradiolicum Fahrbach xx] | 1.134493 | 0.001175 |
| DENOEST_v1_2287 | ID:63769511 Chemotaxis protein [Denitratisoma oestradiolicum Fahrbach xx] | 1.216109 | 0.000206 |
| DENOEST_v1_2286 | ID:63769510 Chemotaxis protein [Denitratisoma oestradiolicum Fahrbach xx] | 1.900779 | 7.69E-06 |
| DENOEST_v1_2285 | ID:63769509 Chemotaxis protein [Denitratisoma oestradiolicum Fahrbach xx] | 0.618297 | 0.03546 |
| DENOEST_v1_2283 | ID:63769507 CheW protein [Denitratisoma oestradiolicum Fahrbach xx] | 0.777004 | 0.003749 |
| DENOEST_v1_2281 | ID:63769505 nemA\| N-ethylmaleimide reductase [Denitratisoma oestradiolicum Fahrbach xx] | -0.20157 | 0.012177 |
| DENOEST_v1_2279 | ID:63769503 Short-chain dehydrogenase/reductase SDR [Denitratisoma oestradiolicum Fahrbach xx] | -0.23369 | 0.007036 |
| DENOEST_v1_2277 | ID:63769501 Vitamin B12-dependent ribonucleotide reductase [Denitratisoma oestradiolicum Fahrbach xx] | -0.87678 | 0.004424 |
| DENOEST_v1_2275 | ID:63769499 Enoyl-CoA hydratase [Denitratisoma oestradiolicum Fahrbach xx] | -0.37565 | 0.002446 |
| DENOEST_v1_2270 | ID:63769494 hoxU\| NAD-reducing hydrogenase HoxS subunit gamma [Denitratisoma oestradiolicum Fahrbach xx] | 0.508622 | 0.01899 |
| DENOEST_v1_2269 | ID:63769493 hoxF\| NAD-reducing hydrogenase HoxS subunit alpha [Denitratisoma oestradiolicum Fahrbach xx] | 0.258759 | 0.026787 |
| DENOEST_v1_2265 | ID:63769489 Purine-binding chemotaxis protein CheW [Denitratisoma oestradiolicum Fahrbach xx] | -0.34507 | 0.036448 |
| DENOEST_v1_2264 | ID:63769488 Methyl-accepting chemotaxis protein [Denitratisoma oestradiolicum Fahrbach xx] | -0.61174 | 0.022995 |
| DENOEST_v1_2263 | ID:63769487 Chemotaxis protein CheA [Denitratisoma oestradiolicum Fahrbach xx] | -0.46446 | 0.017381 |
| DENOEST_v1_2260 | ID:63769484 Two-component system sensor histidine kinase/response regulator [Denitratisoma oestradiolicum Fahrbach xx] | -0.66903 | 0.027371 |
| DENOEST_v1_2253 | ID:63769477 conserved protein of unknown function [Denitratisoma oestradiolicum Fahrbach xx] | -1.34183 | 0.002051 |
| DENOEST_v1_2249 | ID:63769473 conserved exported protein of unknown function [Denitratisoma oestradiolicum Fahrbach xx] | 1.623254 | 0.001403 |
| DENOEST_v1_2247 | ID:63769471 Histidine kinase-, DNA gyrase B-, and HSP90-like ATPase [Denitratisoma oestradiolicum Fahrbach xx] | 0.856667 | 0.005779 |
| DENOEST_v1_2246 | ID:63769470 Response regulator receiver protein [Denitratisoma oestradiolicum Fahrbach xx] | 0.825575 | 0.00052 |
| DENOEST_v1_2245 | ID:63769469 bioD\| dethiobiotin synthetase [Denitratisoma oestradiolicum Fahrbach xx] | 1.119772 | 0.008353 |
| DENOEST_v1_2244 | ID:63769468 3-oxoacyl-[acyl-carrier protein] reductase [Denitratisoma oestradiolicum Fahrbach xx] | 0.808634 | 0.016036 |
| DENOEST_v1_2242 | ID:63769466 Sulfotransferase family protein [Denitratisoma oestradiolicum Fahrbach xx] | 1.796403 | 0.039017 |
| DENOEST_v1_2238 | ID:63769462 conserved protein of unknown function [Denitratisoma oestradiolicum Fahrbach xx] | 2.291684 | 0.003671 |
| DENOEST_v1_2227 | ID:63769451 Electron transfer flavoprotein-ubiquinone oxidoreductase [Denitratisoma oestradiolicum Fahrbach xx] | -0.12319 | 0.033536 |
| DENOEST_v1_2226 | ID:63769450 rne\| fused ribonucleaseE: endoribonuclease ; RNA-binding protein ;RNA degradosome binding protein [Denitratisoma oestradiolicum Fahrbach xx] | -1.70289 | 0.00052 |
| DENOEST_v1_2221 | ID:63769445 Peptidase S49 [Denitratisoma oestradiolicum Fahrbach xx] | -0.9629 | 0.023224 |
| DENOEST_v1_2220 | ID:63769444 Uroporphyrin-III C/tetrapyrrole (Corrin/porphyrin) methyltransferase [Denitratisoma oestradiolicum Fahrbach xx] | -1.05657 | 0.012465 |
| DENOEST_v1_2218 | ID:63769442 conserved protein of unknown function [Denitratisoma oestradiolicum Fahrbach xx] | -1.18716 | 0.000479 |
| DENOEST_v1_2217 | ID:63769441 rpmF\| 50S ribosomal subunit protein L32 [Denitratisoma oestradiolicum Fahrbach xx] | -3.37021 | 0.00843 |
| DENOEST_v1_2214 | ID:63769438 fabD\| malonyl-CoA-[acyl-carrier-protein] transacylase [Denitratisoma oestradiolicum Fahrbach xx] | -0.68092 | 0.00458 |
| DENOEST_v1_2213 | ID:63769437 fabG\| 3-oxoacyl-[acyl-carrier-protein] reductase [Denitratisoma oestradiolicum Fahrbach xx] | -0.62505 | 0.022468 |
| DENOEST_v1_2211 | ID:63769435 fabF\| 3-oxoacyl-[acyl-carrier-protein] synthase II [Denitratisoma oestradiolicum Fahrbach xx] | -0.33827 | 0.02885 |
| DENOEST_v1_2209 | ID:63769433 conserved exported protein of unknown function [Denitratisoma oestradiolicum Fahrbach xx] | 0.375927 | 0.000866 |
| DENOEST_v1_2208 | ID:63769432 exported protein of unknown function [Denitratisoma oestradiolicum Fahrbach xx] | 0.46323 | 0.03894 |
| DENOEST_v1_2207 | ID:63769431 conserved protein of unknown function [Denitratisoma oestradiolicum Fahrbach xx] | 0.762505 | 0.002674 |
| DENOEST_v1_2204 | ID:63769428 nadB\| L-aspartate oxidase (quinolinate synthetase B) [Denitratisoma oestradiolicum Fahrbach xx] | 0.555153 | 0.016697 |
| DENOEST_v1_2199 | ID:63769423 putative periplasmic serine endoprotease DegP-like [Denitratisoma oestradiolicum Fahrbach xx] | -0.65182 | 0.000423 |
| DENOEST_v1_2197 | ID:63769421 lepA\| GTP-binding protein [Denitratisoma oestradiolicum Fahrbach xx] | -0.54814 | 0.023349 |
| DENOEST_v1_2194 | ID:63769418 conserved protein of unknown function [Denitratisoma oestradiolicum Fahrbach xx] | 0.906 | 0.03673 |
| DENOEST_v1_2192 | ID:63769416 era\| membrane-associated, 16S rRNA-binding GTPase [Denitratisoma oestradiolicum Fahrbach xx] | -0.24448 | 0.049702 |
| DENOEST_v1_2190 | ID:63769414 pdxJ\| Pyridoxine 5'-phosphate synthase [Denitratisoma oestradiolicum Fahrbach xx] | -0.71501 | 0.048223 |
| DENOEST_v1_2188 | ID:63769412 nagZ\| Beta-hexosaminidase [Denitratisoma oestradiolicum Fahrbach xx] | -0.44095 | 0.010234 |
| DENOEST_v1_2186 | ID:63769410 MBL fold metallo-hydrolase [Denitratisoma oestradiolicum Fahrbach xx] | -2.39534 | 0.000119 |
| DENOEST_v1_2185 | ID:63769409 Protein involved in RimO-mediated beta-methylthiolation of ribosomal protein S12 YcaO [Denitratisoma oestradiolicum Fahrbach xx] | -2.30892 | 0.047944 |
| DENOEST_v1_2180 | ID:63769404 Iron complex outermembrane recepter protein [Denitratisoma oestradiolicum Fahrbach xx] | 1.18005 | 0.002667 |
| DENOEST_v1_2179 | ID:63769403 uup-A\| ABC transporter ATP-binding protein uup-1 [Denitratisoma oestradiolicum Fahrbach xx] | -0.93502 | 0.044575 |
| DENOEST_v1_2171 | ID:63769395 Secretion system protein E [Denitratisoma oestradiolicum Fahrbach xx] | -0.70579 | 0.0015 |
| DENOEST_v1_2167 | ID:63769391 conserved protein of unknown function [Denitratisoma oestradiolicum Fahrbach xx] | 0.671043 | 0.024896 |
| DENOEST_v1_2166 | ID:63769390 putative Histidine kinase [Denitratisoma oestradiolicum Fahrbach xx] | 1.414538 | 0.012145 |
| DENOEST_v1_2157 | ID:63769381 conserved protein of unknown function [Denitratisoma oestradiolicum Fahrbach xx] | 0.500927 | 0.001006 |
| DENOEST_v1_2155 | ID:63769379 ATP-binding protein [Denitratisoma oestradiolicum Fahrbach xx] | -1.30237 | 9.39E-05 |
| DENOEST_v1_2154 | ID:63769378 Restriction endonuclease subunit S (fragment) [Denitratisoma oestradiolicum Fahrbach xx] | -1.22067 | 0.015148 |
| DENOEST_v1_2153 | ID:63769377 conserved protein of unknown function [Denitratisoma oestradiolicum Fahrbach xx] | -1.31886 | 0.003348 |
| DENOEST_v1_2152 | ID:63769376 conserved protein of unknown function [Denitratisoma oestradiolicum Fahrbach xx] | -0.5319 | 0.002959 |
| DENOEST_v1_2151 | ID:63769375 acnB\| bifunctional aconitate hydratase 2 and 2-methylisocitrate dehydratase [Denitratisoma oestradiolicum Fahrbach xx] | -2.41598 | 5.53E-07 |
| DENOEST_v1_2150 | ID:63769374 Aldolase [Denitratisoma oestradiolicum Fahrbach xx] | -2.29121 | 4.11E-05 |
| DENOEST_v1_2149 | ID:63769373 mdh\| malate dehydrogenase [Denitratisoma oestradiolicum Fahrbach xx] | -2.28073 | 6.1E-05 |
| DENOEST_v1_2148 | ID:63769372 Transcriptional regulator, GntR family [Denitratisoma oestradiolicum Fahrbach xx] | 1.523447 | 4.7E-05 |
| DENOEST_v1_2147 | ID:63769371 Succinate dehydrogenase [Denitratisoma oestradiolicum Fahrbach xx] | -1.56582 | 0.015148 |
| DENOEST_v1_2145 | ID:63769369 sdhA\| succinate dehydrogenase, flavoprotein subunit [Denitratisoma oestradiolicum Fahrbach xx] | -1.45227 | 2.34E-05 |
| DENOEST_v1_2144 | ID:63769368 sdhB\| succinate dehydrogenase, iron-sulfur subunit [Denitratisoma oestradiolicum Fahrbach xx] | -1.37699 | 0.000971 |
| DENOEST_v1_2142 | ID:63769366 gltA\| citrate synthase [Denitratisoma oestradiolicum Fahrbach xx] | -1.87398 | 2.22E-06 |
| DENOEST_v1_2141 | ID:63769365 sucA\| 2-oxoglutarate decarboxylase, thiamin-requiring [Denitratisoma oestradiolicum Fahrbach xx] | -2.96267 | 2.23E-06 |
| DENOEST_v1_2140 | ID:63769364 sucB\| dihydrolipoyltranssuccinase [Denitratisoma oestradiolicum Fahrbach xx] | -2.959 | 0.000535 |
| DENOEST_v1_2139 | ID:63769363 odhL\| Dihydrolipoyl dehydrogenase [Denitratisoma oestradiolicum Fahrbach xx] | -2.96047 | 2.62E-06 |
| DENOEST_v1_2138 | ID:63769362 zapE\| Cell division protein ZapE [Denitratisoma oestradiolicum Fahrbach xx] | -1.8539 | 0.002075 |
| DENOEST_v1_2135 | ID:63769359 slyD\| FKBP-type peptidyl-prolyl cis-trans isomerase SlyD [Denitratisoma oestradiolicum Fahrbach xx] | -0.67233 | 0.003122 |
| DENOEST_v1_2122 | ID:63769346 conserved protein of unknown function [Denitratisoma oestradiolicum Fahrbach xx] | -0.51244 | 0.009055 |
| DENOEST_v1_2117 | ID:63769341 TonB-dependent receptor [Denitratisoma oestradiolicum Fahrbach xx] | -21.3758 | 0.035763 |
| DENOEST_v1_2115 | ID:63769339 putative Electron transfer flavoprotein, beta subunit [Denitratisoma oestradiolicum Fahrbach xx] | 2.395123 | 0.031559 |
| DENOEST_v1_2114 | ID:63769338 conserved protein of unknown function [Denitratisoma oestradiolicum Fahrbach xx] | 1.939707 | 0.000516 |
| DENOEST_v1_2111 | ID:63769335 cutM\| Carbon monoxide dehydrogenase medium chain [Denitratisoma oestradiolicum Fahrbach xx] | 1.244268 | 0.001177 |
| DENOEST_v1_2108 | ID:63769332 Aldehyde oxidase and xanthine dehydrogenase molybdopterin binding protein [Denitratisoma oestradiolicum Fahrbach xx] | 1.112286 | 0.000738 |
| DENOEST_v1_2105 | ID:63769329 conserved protein of unknown function [Denitratisoma oestradiolicum Fahrbach xx] | 2.194105 | 0.002379 |
| DENOEST_v1_2102 | ID:63769326 Electron transfer flavoprotein-associated cytochrome b and CCG domain pair iron-sulfur cluster-binding oxidoreductase [Denitratisoma oestradiolicum Fahrbach xx] | 1.723287 | 1.92E-05 |
| DENOEST_v1_2099 | ID:63769323 putative NADH oxidase [Denitratisoma oestradiolicum Fahrbach xx] | 1.925327 | 0.001356 |
| DENOEST_v1_2098 | ID:63769322 conserved protein of unknown function [Denitratisoma oestradiolicum Fahrbach xx] | 1.203761 | 0.001134 |
| DENOEST_v1_2097 | ID:63769321 conserved protein of unknown function [Denitratisoma oestradiolicum Fahrbach xx] | 2.583533 | 0.001822 |
| DENOEST_v1_2095 | ID:63769319 putative Electron transfer flavoprotein subunit alpha [Denitratisoma oestradiolicum Fahrbach xx] | 2.604377 | 1.49E-05 |
| DENOEST_v1_2090 | ID:63769314 L-carnitine dehydratase/bile acid-inducible protein F [Denitratisoma oestradiolicum Fahrbach xx] | 0.62538 | 0.001183 |
| DENOEST_v1_2086 | ID:63769310 2-nitropropane dioxygenase NPD [Denitratisoma oestradiolicum Fahrbach xx] | 2.144829 | 0.000197 |
| DENOEST_v1_2084 | ID:63769308 putative Electron transfer flavoprotein, beta subunit [Denitratisoma oestradiolicum Fahrbach xx] | 2.523859 | 0.000678 |
| DENOEST_v1_2082 | ID:63769306 LLM class F420-dependent oxidoreductase [Denitratisoma oestradiolicum Fahrbach xx] | 0.638906 | 0.000136 |
| DENOEST_v1_2079 | ID:63769303 conserved protein of unknown function [Denitratisoma oestradiolicum Fahrbach xx] | -1.80294 | 0.006583 |
| DENOEST_v1_2072 | ID:63769296 conserved exported protein of unknown function [Denitratisoma oestradiolicum Fahrbach xx] | -0.86126 | 0.002381 |
| DENOEST_v1_2062 | ID:63769286 yfbE\| uridine 5'-(beta-1-threo-pentapyranosyl-4-ulose diphosphate) aminotransferase, PLP-dependent [Denitratisoma oestradiolicum Fahrbach xx] | 0.310352 | 0.005564 |
| DENOEST_v1_2058 | ID:63769282 yfbQ\| putative aminotransferase [Denitratisoma oestradiolicum Fahrbach xx] | -0.37676 | 0.011198 |
| DENOEST_v1_2057 | ID:63769281 hom\| Homoserine dehydrogenase [Denitratisoma oestradiolicum Fahrbach xx] | -0.87276 | 0.00181 |
| DENOEST_v1_2052 | ID:63769276 fadE\| Acyl-CoA dehydrogenase FadE26 [Denitratisoma oestradiolicum Fahrbach xx] | 0.354629 | 0.029496 |
| DENOEST_v1_2051 | ID:63769275 conserved protein of unknown function [Denitratisoma oestradiolicum Fahrbach xx] | 1.00558 | 0.001509 |
| DENOEST_v1_2050 | ID:63769274 conserved protein of unknown function [Denitratisoma oestradiolicum Fahrbach xx] | 1.12528 | 0.00051 |
| DENOEST_v1_2047 | ID:63769271 Acyl-CoA synthetase (AMP-forming)/AMP-acid ligase II [Denitratisoma oestradiolicum Fahrbach xx] | 0.923039 | 0.000365 |
| DENOEST_v1_2044 | ID:63769268 putative enoyl-CoA hydratase 1 [Denitratisoma oestradiolicum Fahrbach xx] | -1.37151 | 0.004882 |
| DENOEST_v1_2040 | ID:63769264 calB\| putative coniferyl aldehyde dehydrogenase [Denitratisoma oestradiolicum Fahrbach xx] | 0.562509 | 0.043381 |
| DENOEST_v1_2036 | ID:63769260 Twin-arginine translocation pathway signal protein [Denitratisoma oestradiolicum Fahrbach xx] | 0.549996 | 0.000626 |
| DENOEST_v1_2031 | ID:63769255 rlmD\| 23S rRNA (uracil(1939)-C(5))-methyltransferase RlmD [Denitratisoma oestradiolicum Fahrbach xx] | -0.97956 | 0.011627 |
| DENOEST_v1_2028 | ID:63769252 ratA\| Ribosome association toxin RatA [Denitratisoma oestradiolicum Fahrbach xx] | -1.57293 | 0.012856 |
| DENOEST_v1_2027 | ID:63769251 yfjF\| hypothetical protein [Denitratisoma oestradiolicum Fahrbach xx] | -0.49426 | 0.008986 |
| DENOEST_v1_2026 | ID:63769250 guaB\| IMP dehydrogenase [Denitratisoma oestradiolicum Fahrbach xx] | -0.89316 | 0.000159 |
| DENOEST_v1_2019 | ID:63769243 Single-stranded DNA-binding protein [Denitratisoma oestradiolicum Fahrbach xx] | -0.91552 | 0.01153 |
| DENOEST_v1_1979 | ID:63769203 Anaerobic dehydrogenase typically selenocysteine-containing [Denitratisoma oestradiolicum Fahrbach xx] | 0.914493 | 0.005223 |
| DENOEST_v1_1966 | ID:63769190 Plasmid stability protein StbA [Denitratisoma oestradiolicum Fahrbach xx] | -1.60132 | 0.004394 |
| DENOEST_v1_1954 | ID:63769178 Phosphonate ABC transporter permease [Denitratisoma oestradiolicum Fahrbach xx] | -0.87646 | 0.001576 |
| DENOEST_v1_1953 | ID:63769177 DNA helicase [Denitratisoma oestradiolicum Fahrbach xx] | -0.64206 | 0.001413 |
| DENOEST_v1_1951 | ID:63769175 conserved protein of unknown function [Denitratisoma oestradiolicum Fahrbach xx] | -0.59372 | 0.002027 |
| DENOEST_v1_1902 | ID:63769126 hupB\| DNA-binding protein HU-beta [Denitratisoma oestradiolicum Fahrbach xx] | -1.24593 | 0.020017 |
| DENOEST_v1_1899 | ID:63769123 Integrase [Denitratisoma oestradiolicum Fahrbach xx] | -0.6101 | 0.022183 |
| DENOEST_v1_1894 | ID:63769118 conserved protein of unknown function [Denitratisoma oestradiolicum Fahrbach xx] | 2.523447 | 0.01221 |
| DENOEST_v1_1889 | ID:63769113 conserved exported protein of unknown function [Denitratisoma oestradiolicum Fahrbach xx] | -2.5396 | 0.014845 |
| DENOEST_v1_1882 | ID:63769106 Multidrug efflux system subunit MdtA [Denitratisoma oestradiolicum Fahrbach xx] | 3.144902 | 0.045448 |
| DENOEST_v1_1871 | ID:63769095 conserved protein of unknown function [Denitratisoma oestradiolicum Fahrbach xx] | 1.477441 | 0.019724 |
| DENOEST_v1_1854 | ID:63769078 conserved protein of unknown function [Denitratisoma oestradiolicum Fahrbach xx] | -1.34509 | 0.014305 |
| DENOEST_v1_1853 | ID:63769077 Single-stranded DNA-binding protein [Denitratisoma oestradiolicum Fahrbach xx] | -0.68352 | 0.000988 |
| DENOEST_v1_1784 | ID:63769008 conserved protein of unknown function [Denitratisoma oestradiolicum Fahrbach xx] | -0.32733 | 0.010569 |
| DENOEST_v1_1773 | ID:63768997 hupB\| HU, DNA-binding transcriptional regulator, beta subunit [Denitratisoma oestradiolicum Fahrbach xx] | -0.57564 | 0.031285 |
| DENOEST_v1_1768 | ID:63768992 conserved protein of unknown function [Denitratisoma oestradiolicum Fahrbach xx] | -0.55277 | 0.019243 |
| DENOEST_v1_1754 | ID:63768978 Diheme cytochrome c [Denitratisoma oestradiolicum Fahrbach xx] | 1.174101 | 0.010041 |
| DENOEST_v1_1743 | ID:63768967 PPOX class F420-dependent enzyme [Denitratisoma oestradiolicum Fahrbach xx] | 0.507269 | 0.004784 |
| DENOEST_v1_1738 | ID:63768962 bcp\| putative peroxiredoxin bcp [Denitratisoma oestradiolicum Fahrbach xx] | -0.67537 | 0.006546 |
| DENOEST_v1_1736 | ID:63768960 conserved protein of unknown function [Denitratisoma oestradiolicum Fahrbach xx] | -0.84647 | 0.002249 |
| DENOEST_v1_1733 | ID:63768957 holB\| DNA-directed DNA polymerase III, delta subunit [Denitratisoma oestradiolicum Fahrbach xx] | -0.82531 | 0.00054 |
| DENOEST_v1_1729 | ID:63768953 NDMA-dependent alcohol dehydrogenase [Denitratisoma oestradiolicum Fahrbach xx] | 0.414449 | 0.012419 |
| DENOEST_v1_1715 | ID:63768939 efp\| Elongation factor P [Denitratisoma oestradiolicum Fahrbach xx] | -1.78623 | 0.000417 |
| DENOEST_v1_1712 | ID:63768936 tadA\| tRNA-specific adenosine deaminase [Denitratisoma oestradiolicum Fahrbach xx] | -0.49143 | 0.013295 |
| DENOEST_v1_1710 | ID:63768934 ppdK\| Pyruvate, phosphate dikinase [Denitratisoma oestradiolicum Fahrbach xx] | 0.672621 | 0.000555 |
| DENOEST_v1_1708 | ID:63768932 Pyridoxamine 5'-phosphate oxidase [Denitratisoma oestradiolicum Fahrbach xx] | 0.613865 | 0.046975 |
| DENOEST_v1_1706 | ID:63768930 Type II restriction endonuclease [Denitratisoma oestradiolicum Fahrbach xx] | -0.94625 | 0.022339 |
| DENOEST_v1_1702 | ID:63768926 cusA\| copper/silver efflux system, membrane component [Denitratisoma oestradiolicum Fahrbach xx] | 16.26717 | 0.003 |
| DENOEST_v1_1701 | ID:63768925 conserved protein of unknown function [Denitratisoma oestradiolicum Fahrbach xx] | -0.63417 | 0.022059 |
| DENOEST_v1_1700 | ID:63768924 Transporter [Denitratisoma oestradiolicum Fahrbach xx] | -0.48461 | 0.010274 |
| DENOEST_v1_1698 | ID:63768922 Cytochrome c family protein [Denitratisoma oestradiolicum Fahrbach xx] | -2.3596 | 3.63E-05 |
| DENOEST_v1_1695 | ID:63768919 conserved exported protein of unknown function [Denitratisoma oestradiolicum Fahrbach xx] | 0.64117 | 0.006278 |
| DENOEST_v1_1691 | ID:63768915 rnfC\| Ion-translocating oxidoreductase complex subunit C [Denitratisoma oestradiolicum Fahrbach xx] | -0.31508 | 0.001248 |
| DENOEST_v1_1689 | ID:63768913 rsxG\| putative oxidoreductase [Denitratisoma oestradiolicum Fahrbach xx] | -0.32899 | 0.000466 |
| DENOEST_v1_1686 | ID:63768910 conserved protein of unknown function [Denitratisoma oestradiolicum Fahrbach xx] | -0.97984 | 0.020837 |
| DENOEST_v1_1685 | ID:63768909 conserved hypothetical protein; putative ATPase [Denitratisoma oestradiolicum Fahrbach xx] | -0.7132 | 0.01427 |
| DENOEST_v1_1677 | ID:63768901 conserved exported protein of unknown function [Denitratisoma oestradiolicum Fahrbach xx] | 2.554235 | 0.047587 |
| DENOEST_v1_1671 | ID:63768895 rmuC\| DNA recombination protein RmuC homolog [Denitratisoma oestradiolicum Fahrbach xx] | -0.40646 | 0.014992 |
| DENOEST_v1_1669 | ID:63768893 fabI\| enoyl-[acyl-carrier-protein] reductase, NADH-dependent [Denitratisoma oestradiolicum Fahrbach xx] | -0.36486 | 0.030889 |
| DENOEST_v1_1668 | ID:63768892 futA\| Iron uptake protein A1 [Denitratisoma oestradiolicum Fahrbach xx] | -1.71913 | 3.83E-05 |
| DENOEST_v1_1663 | ID:63768887 pyrD\| dihydro-orotate oxidase, FMN-linked [Denitratisoma oestradiolicum Fahrbach xx] | -0.49384 | 0.019036 |
| DENOEST_v1_1650 | ID:63768874 ABC transporter ATP-binding protein [Denitratisoma oestradiolicum Fahrbach xx] | -0.76062 | 0.000755 |
| DENOEST_v1_1648 | ID:63768872 Efflux transporter, RND family, MFP subunit [Denitratisoma oestradiolicum Fahrbach xx] | -1.1872 | 0.012953 |
| DENOEST_v1_1647 | ID:63768871 TetR/AcrR family transcriptional regulator [Denitratisoma oestradiolicum Fahrbach xx] | -1.19741 | 0.00039 |
| DENOEST_v1_1644 | ID:63768868 linC\| 2,5-dichloro-2,5-cyclohexadiene-1,4-diol dehydrogenase [Denitratisoma oestradiolicum Fahrbach xx] | 0.336017 | 0.003622 |
| DENOEST_v1_1636 | ID:63768860 Alkyl hydroperoxide reductase AhpD [Denitratisoma oestradiolicum Fahrbach xx] | 2.425281 | 0.016138 |
| DENOEST_v1_1616 | ID:63768840 conserved protein of unknown function [Denitratisoma oestradiolicum Fahrbach xx] | -1.37047 | 0.006553 |
| DENOEST_v1_1614 | ID:63768838 protein of unknown function [Denitratisoma oestradiolicum Fahrbach xx] | -1.62768 | 0.000755 |
| DENOEST_v1_1612 | ID:63768836 Transcription elongation factor GreAB (modular protein) [Denitratisoma oestradiolicum Fahrbach xx] | -3.12865 | 0.000193 |
| DENOEST_v1_1605 | ID:63768829 yliG\| putative AdoMet-dependent methyltransferase, UPF0004 family [Denitratisoma oestradiolicum Fahrbach xx] | -0.21297 | 0.002111 |
| DENOEST_v1_1600 | ID:63768824 mvaB\| Hydroxymethylglutaryl-CoA lyase [Denitratisoma oestradiolicum Fahrbach xx] | 0.601187 | 0.02015 |
| DENOEST_v1_1599 | ID:63768823 accA\| Biotin carboxylase / Biotin carboxyl carrier protein [Denitratisoma oestradiolicum Fahrbach xx] | 1.045268 | 0.005157 |
| DENOEST_v1_1598 | ID:63768822 Enoyl-CoA hydratase [Denitratisoma oestradiolicum Fahrbach xx] | 0.440306 | 0.031178 |
| DENOEST_v1_1596 | ID:63768820 mmgC\| Acyl-CoA dehydrogenase [Denitratisoma oestradiolicum Fahrbach xx] | 0.546088 | 0.019064 |
| DENOEST_v1_1595 | ID:63768819 phaR\| Polyhydroxyalkanoate synthesis repressor PhaR [Denitratisoma oestradiolicum Fahrbach xx] | 0.703814 | 0.007159 |
| DENOEST_v1_1594 | ID:63768818 fabG\| 3-oxoacyl-[acyl-carrier-protein] reductase [Denitratisoma oestradiolicum Fahrbach xx] | -2.07484 | 6.39E-06 |
| DENOEST_v1_1593 | ID:63768817 fabG\| 3-oxoacyl-[acyl-carrier-protein] reductase [Denitratisoma oestradiolicum Fahrbach xx] | -0.55772 | 0.00672 |
| DENOEST_v1_1592 | ID:63768816 phaC\| Poly(3-hydroxyalkanoate) polymerase subunit PhaC [Denitratisoma oestradiolicum Fahrbach xx] | -1.0977 | 0.004644 |
| DENOEST_v1_1586 | ID:63768810 Oxidoreductase [Denitratisoma oestradiolicum Fahrbach xx] | -0.51633 | 0.011177 |
| DENOEST_v1_1585 | ID:63768809 Oxidoreductase [Denitratisoma oestradiolicum Fahrbach xx] | -0.42592 | 0.013844 |
| DENOEST_v1_1581 | ID:63768805 purL\| phosphoribosylformyl-glycineamide synthetase [Denitratisoma oestradiolicum Fahrbach xx] | -0.76506 | 4.14E-05 |
| DENOEST_v1_1579 | ID:63768803 phoB\| DNA-binding response regulator in two-component regulatory system with PhoR (or CreC) [Denitratisoma oestradiolicum Fahrbach xx] | -0.1645 | 0.03058 |
| DENOEST_v1_1578 | ID:63768802 phoU\| negative regulator of PhoR/PhoB two-component regulator [Denitratisoma oestradiolicum Fahrbach xx] | 0.363161 | 0.032296 |
| DENOEST_v1_1574 | ID:63768798 pstS\| phosphate transporter subunit ; periplasmic-binding component of ABC superfamily [Denitratisoma oestradiolicum Fahrbach xx] | -1.21155 | 0.000786 |
| DENOEST_v1_1570 | ID:63768794 conserved exported protein of unknown function [Denitratisoma oestradiolicum Fahrbach xx] | -19.783 | 0.00462 |
| DENOEST_v1_1568 | ID:63768792 napC\| nitrate reductase, cytochrome c-type,periplasmic [Denitratisoma oestradiolicum Fahrbach xx] | -17.9545 | 0.004305 |
| DENOEST_v1_1566 | ID:63768790 Histidine kinase [Denitratisoma oestradiolicum Fahrbach xx] | -1.12688 | 0.003221 |
| DENOEST_v1_1562 | ID:63768786 conserved protein of unknown function [Denitratisoma oestradiolicum Fahrbach xx] | 1.079643 | 0.019895 |
| DENOEST_v1_1548 | ID:63768772 hscA\| DnaK-like molecular chaperone specific for IscU [Denitratisoma oestradiolicum Fahrbach xx] | -0.5368 | 0.008781 |
| DENOEST_v1_1547 | ID:63768771 hscB\| Co-chaperone protein HscB homolog [Denitratisoma oestradiolicum Fahrbach xx] | -0.65934 | 0.001038 |
| DENOEST_v1_1546 | ID:63768770 iscA\| iron-binding protein , believed to be involved in Fe-S protein formation or repair [Denitratisoma oestradiolicum Fahrbach xx] | -1.67845 | 0.000129 |
| DENOEST_v1_1545 | ID:63768769 iscU\| iron-binding protein believed to be involved in Fe-S protein formation or repair; function as a scaffold for the assembly of the transiently bound Fe/S cluster [Denitratisoma oestradiolicum Fahrbach xx] | -0.885 | 0.005305 |
| DENOEST_v1_1544 | ID:63768768 iscS\| cysteine desulfurase used in synthesis of Fe-S cluster (tRNA 4-thiouridine sulfurtransferase ) [Denitratisoma oestradiolicum Fahrbach xx] | -0.56528 | 0.011235 |
| DENOEST_v1_1543 | ID:63768767 iscS\| cysteine desulfurase (tRNA sulfurtransferase), PLP-dependent [Denitratisoma oestradiolicum Fahrbach xx] | -0.68139 | 0.003934 |
| DENOEST_v1_1542 | ID:63768766 iscR\| DNA-binding transcriptional repressor [Denitratisoma oestradiolicum Fahrbach xx] | -1.43386 | 0.000987 |
| DENOEST_v1_1540 | ID:63768764 sucD\| succinyl-CoA synthetase, NAD(P)-binding, alpha subunit [Denitratisoma oestradiolicum Fahrbach xx] | -1.26745 | 0.000182 |
| DENOEST_v1_1539 | ID:63768763 sucC\| succinyl-CoA synthetase, beta subunit [Denitratisoma oestradiolicum Fahrbach xx] | -1.28033 | 3.33E-05 |
| DENOEST_v1_1538 | ID:63768762 Phenylacetic acid degradation protein PaaN [Denitratisoma oestradiolicum Fahrbach xx] | 0.495697 | 0.010482 |
| DENOEST_v1_1535 | ID:63768759 lpd\| lipoamide dehydrogenase, E3 component is part of three enzyme complexes [Denitratisoma oestradiolicum Fahrbach xx] | -1.27293 | 5.34E-06 |
| DENOEST_v1_1533 | ID:63768757 pdhB\| Dihydrolipoyllysine-residue acetyltransferase component of pyruvate dehydrogenase complex [Denitratisoma oestradiolicum Fahrbach xx] | -1.37124 | 0.000321 |
| DENOEST_v1_1532 | ID:63768756 aceE\| pyruvate dehydrogenase, decarboxylase component E1, thiamin-binding [Denitratisoma oestradiolicum Fahrbach xx] | -1.61275 | 0.000105 |
| DENOEST_v1_1527 | ID:63768751 dksA\| DNA-binding transcriptional regulator of rRNA transcription, DnaK suppressor protein [Denitratisoma oestradiolicum Fahrbach xx] | -0.47224 | 0.014086 |
| DENOEST_v1_1526 | ID:63768750 pnp\| polyribonucleotide nucleotidyltransferase (Polynucleotide phosphorylase) (PNPase) [Denitratisoma oestradiolicum Fahrbach xx] | -0.63042 | 0.000756 |
| DENOEST_v1_1525 | ID:63768749 rpsO\| 30S ribosomal protein S15 [Denitratisoma oestradiolicum Fahrbach xx] | -1.37643 | 0.000478 |
| DENOEST_v1_1523 | ID:63768747 rbfA\| Ribosome-binding factor A [Denitratisoma oestradiolicum Fahrbach xx] | -0.46424 | 0.014936 |
| DENOEST_v1_1522 | ID:63768746 infB\| translation initiation factor IF-2 [Denitratisoma oestradiolicum Fahrbach xx] | -0.6956 | 0.000345 |
| DENOEST_v1_1521 | ID:63768745 nusA\| transcription termination/antitermination L factor [Denitratisoma oestradiolicum Fahrbach xx] | -0.79018 | 0.000289 |
| DENOEST_v1_1520 | ID:63768744 yhbC\| conserved hypothetical protein [Denitratisoma oestradiolicum Fahrbach xx] | -0.89937 | 0.011014 |
| DENOEST_v1_1516 | ID:63768740 trpS\| Tryptophan--tRNA ligase [Denitratisoma oestradiolicum Fahrbach xx] | -0.41431 | 0.010277 |
| DENOEST_v1_1514 | ID:63768738 yciO\| conserved hypothetical protein [Denitratisoma oestradiolicum Fahrbach xx] | -1.72783 | 0.002432 |
| DENOEST_v1_1513 | ID:63768737 yciV\| conserved hypothetical protein [Denitratisoma oestradiolicum Fahrbach xx] | -1.28601 | 0.007287 |
| DENOEST_v1_1512 | ID:63768736 Alpha/beta hydrolase [Denitratisoma oestradiolicum Fahrbach xx] | -1.30183 | 0.000887 |
| DENOEST_v1_1511 | ID:63768735 gcvT\| aminomethyltransferase, tetrahydrofolate-dependent, subunit (T protein) of glycine cleavage complex [Denitratisoma oestradiolicum Fahrbach xx] | 1.582151 | 0.000743 |
| DENOEST_v1_1510 | ID:63768734 gcvH\| glycine cleavage complex lipoylprotein [Denitratisoma oestradiolicum Fahrbach xx] | 0.750218 | 0.024219 |
| DENOEST_v1_1509 | ID:63768733 gcvP\| glycine decarboxylase, PLP-dependent, subunit (protein P) of glycine cleavage complex [Denitratisoma oestradiolicum Fahrbach xx] | 1.384457 | 6.16E-05 |
| DENOEST_v1_1508 | ID:63768732 conserved exported protein of unknown function [Denitratisoma oestradiolicum Fahrbach xx] | 2.107617 | 0.000494 |
| DENOEST_v1_1507 | ID:63768731 conserved exported protein of unknown function [Denitratisoma oestradiolicum Fahrbach xx] | 3.194624 | 0.011102 |
| DENOEST_v1_1505 | ID:63768729 gdhA\| Glutamate dehydrogenase [Denitratisoma oestradiolicum Fahrbach xx] | 0.601648 | 0.000211 |
| DENOEST_v1_1504 | ID:63768728 Antitoxin (fragment) [Denitratisoma oestradiolicum Fahrbach xx] | -0.5774 | 0.021265 |
| DENOEST_v1_1503 | ID:63768727 putative toxin-antitoxin system toxin component, PIN family [Denitratisoma oestradiolicum Fahrbach xx] | -5.5146 | 0.043945 |
| DENOEST_v1_1500 | ID:63768724 Putative endonuclease (modular protein) [Denitratisoma oestradiolicum Fahrbach xx] | -1.16713 | 0.001403 |
| DENOEST_v1_1497 | ID:63768721 purN\| Phosphoribosylglycinamide formyltransferase [Denitratisoma oestradiolicum Fahrbach xx] | -0.39297 | 0.007001 |
| DENOEST_v1_1494 | ID:63768718 conserved protein of unknown function [Denitratisoma oestradiolicum Fahrbach xx] | -0.43708 | 0.017284 |
| DENOEST_v1_1492 | ID:63768716 lemA\| Protein LemA [Denitratisoma oestradiolicum Fahrbach xx] | 0.360046 | 0.001601 |
| DENOEST_v1_1491 | ID:63768715 Carnitine dehydratase [Denitratisoma oestradiolicum Fahrbach xx] | 0.586501 | 0.005039 |
| DENOEST_v1_1488 | ID:63768712 ycgR\| Flagellar brake protein YcgR [Denitratisoma oestradiolicum Fahrbach xx] | 0.719868 | 0.025243 |
| DENOEST_v1_1481 | ID:63768705 bcp\| Peroxiredoxin Bcp [Denitratisoma oestradiolicum Fahrbach xx] | -0.71875 | 0.000215 |
| DENOEST_v1_1472 | ID:63768696 acs\| acetyl-CoA synthetase [Denitratisoma oestradiolicum Fahrbach xx] | -2.35836 | 1.24E-05 |
| DENOEST_v1_1470 | ID:63768694 Fumarate hydratase class I [Denitratisoma oestradiolicum Fahrbach xx] | -0.48886 | 0.002741 |
| DENOEST_v1_1468 | ID:63768692 murI\| Glutamate racemase [Denitratisoma oestradiolicum Fahrbach xx] | -0.8501 | 0.026405 |
| DENOEST_v1_1461 | ID:63768685 ilvC\| acetohydroxy acid isomeroreductase and 2-dehydropantoate 2-reductase [Denitratisoma oestradiolicum Fahrbach xx] | -0.81203 | 0.000996 |
| DENOEST_v1_1460 | ID:63768684 ilvH\| acetolactate synthase isozyme III, small subunit [Denitratisoma oestradiolicum Fahrbach xx] | -0.78197 | 0.007063 |
| DENOEST_v1_1459 | ID:63768683 ilvI\| acetolactate synthase III, large subunit [Denitratisoma oestradiolicum Fahrbach xx] | -0.64348 | 0.00112 |
| DENOEST_v1_1456 | ID:63768680 conserved exported protein of unknown function [Denitratisoma oestradiolicum Fahrbach xx] | -0.49675 | 0.012336 |
| DENOEST_v1_1452 | ID:63768676 pepA\| aminopeptidase A, a cyteinylglycinase [Denitratisoma oestradiolicum Fahrbach xx] | -0.41546 | 0.016486 |
| DENOEST_v1_1449 | ID:63768673 putative 4-hydroxy-4-methyl-2-oxoglutarate aldolase [Denitratisoma oestradiolicum Fahrbach xx] | -0.91461 | 0.002098 |
| DENOEST_v1_1445 | ID:63768669 conserved protein of unknown function [Denitratisoma oestradiolicum Fahrbach xx] | 14.48096 | 0.023326 |
| DENOEST_v1_1429 | ID:63768653 L518 [Denitratisoma oestradiolicum Fahrbach xx] | 1.676858 | 0.04066 |
| DENOEST_v1_1428 | ID:63768652 galE\| UDP-galactose-4-epimerase [Denitratisoma oestradiolicum Fahrbach xx] | 0.664748 | 0.011138 |
| DENOEST_v1_1416 | ID:63768640 conserved protein of unknown function [Denitratisoma oestradiolicum Fahrbach xx] | 0.591417 | 0.021093 |
| DENOEST_v1_1414 | ID:63768638 Secretion system protein E [Denitratisoma oestradiolicum Fahrbach xx] | 0.405767 | 0.010952 |
| DENOEST_v1_1413 | ID:63768637 conserved protein of unknown function [Denitratisoma oestradiolicum Fahrbach xx] | 0.398297 | 0.042687 |
| DENOEST_v1_1411 | ID:63768635 Adenylate/guanylate cyclase with Chase sensor [Denitratisoma oestradiolicum Fahrbach xx] | 0.329154 | 0.00288 |
| DENOEST_v1_1408 | ID:63768632 Serine/threonine protein kinase [Denitratisoma oestradiolicum Fahrbach xx] | 0.184097 | 0.038922 |
| DENOEST_v1_1406 | ID:63768630 Peptidylprolyl isomerase [Denitratisoma oestradiolicum Fahrbach xx] | -0.60524 | 0.000172 |
| DENOEST_v1_1404 | ID:63768628 conserved hypothetical protein; putative membrane protease subunit [Denitratisoma oestradiolicum Fahrbach xx] | 0.646597 | 0.000527 |
| DENOEST_v1_1402 | ID:63768626 pps\| phosphoenolpyruvate synthase [Denitratisoma oestradiolicum Fahrbach xx] | 0.278876 | 0.023737 |
| DENOEST_v1_1393 | ID:63768617 SapC family protein [Denitratisoma oestradiolicum Fahrbach xx] | -0.354 | 0.038354 |
| DENOEST_v1_1392 | ID:63768616 SAM-dependent methyltransferase [Denitratisoma oestradiolicum Fahrbach xx] | -0.567 | 0.010507 |
| DENOEST_v1_1391 | ID:63768615 thrC\| Threonine synthase [Denitratisoma oestradiolicum Fahrbach xx] | -0.57056 | 0.002951 |
| DENOEST_v1_1389 | ID:63768613 putative O-succinylbenzoate--CoA ligase [Denitratisoma oestradiolicum Fahrbach xx] | 1.646189 | 0.00056 |
| DENOEST_v1_1388 | ID:63768612 conserved protein of unknown function [Denitratisoma oestradiolicum Fahrbach xx] | 1.496598 | 0.001176 |
| DENOEST_v1_1387 | ID:63768611 Carbon monoxide dehydrogenase subunit G [Denitratisoma oestradiolicum Fahrbach xx] | 2.68907 | 1.66E-05 |
| DENOEST_v1_1386 | ID:63768610 rpfG\| Cyclic di-GMP phosphodiesterase response regulator RpfG [Denitratisoma oestradiolicum Fahrbach xx] | 0.599194 | 0.016413 |
| DENOEST_v1_1385 | ID:63768609 putative two-component system sensor protein [Denitratisoma oestradiolicum Fahrbach xx] | 0.570202 | 0.033117 |
| DENOEST_v1_1381 | ID:63768605 Sulfurtransferase [Denitratisoma oestradiolicum Fahrbach xx] | 0.650192 | 0.026418 |
| DENOEST_v1_1379 | ID:63768603 conserved protein of unknown function [Denitratisoma oestradiolicum Fahrbach xx] | 1.86509 | 0.01175 |
| DENOEST_v1_1378 | ID:63768602 fadD\| 3-[(3aS,4S,7aS)-7a-methyl-1,5-dioxo-octahydro-1H-inden-4-yl]propanoyl:CoA ligase [Denitratisoma oestradiolicum Fahrbach xx] | 1.205387 | 0.000473 |
| DENOEST_v1_1377 | ID:63768601 3-oxosteroid 1-dehydrogenase [Denitratisoma oestradiolicum Fahrbach xx] | 1.699254 | 0.00098 |
| DENOEST_v1_1376 | ID:63768600 ksi\| Steroid Delta-isomerase [Denitratisoma oestradiolicum Fahrbach xx] | 2.504451 | 0.003406 |
| DENOEST_v1_1375 | ID:63768599 fadA\| 3-ketoacyl-CoA thiolase [Denitratisoma oestradiolicum Fahrbach xx] | 1.722967 | 0.001541 |
| DENOEST_v1_1374 | ID:63768598 putative enoyl-CoA hydratase 1 [Denitratisoma oestradiolicum Fahrbach xx] | 2.017298 | 0.000191 |
| DENOEST_v1_1373 | ID:63768597 3-oxoacyl-[acyl-carrier protein] reductase [Denitratisoma oestradiolicum Fahrbach xx] | 1.626772 | 0.000425 |
| DENOEST_v1_1372 | ID:63768596 Short-chain dehydrogenase [Denitratisoma oestradiolicum Fahrbach xx] | 1.485235 | 0.000703 |
| DENOEST_v1_1371 | ID:63768595 fadA\| Steroid 3-ketoacyl-CoA thiolase [Denitratisoma oestradiolicum Fahrbach xx] | 2.486836 | 4.55E-05 |
| DENOEST_v1_1370 | ID:63768594 Butyryl-CoA dehydrogenase [Denitratisoma oestradiolicum Fahrbach xx] | 2.557033 | 0.000756 |
| DENOEST_v1_1369 | ID:63768593 Acyl-CoA dehydrogenase [Denitratisoma oestradiolicum Fahrbach xx] | 2.317668 | 0.003511 |
| DENOEST_v1_1368 | ID:63768592 Enoyl-CoA hydratase [Denitratisoma oestradiolicum Fahrbach xx] | 2.215467 | 0.003962 |
| DENOEST_v1_1367 | ID:63768591 Glutaconate CoA-transferase subunit B [Denitratisoma oestradiolicum Fahrbach xx] | 2.622831 | 6.13E-05 |
| DENOEST_v1_1366 | ID:63768590 putative enzyme [Denitratisoma oestradiolicum Fahrbach xx] | 2.634505 | 6.82E-06 |
| DENOEST_v1_1353 | ID:63768577 qrcC\| Menaquinone reductase, iron-sulfur cluster-binding subunit [Denitratisoma oestradiolicum Fahrbach xx] | 1.642793 | 0.029712 |
| DENOEST_v1_1352 | ID:63768576 Molybdopterin oxidoreductase [Denitratisoma oestradiolicum Fahrbach xx] | 1.178828 | 0.002159 |
| DENOEST_v1_1347 | ID:63768571 yfhQ\| putative methyltransferase [Denitratisoma oestradiolicum Fahrbach xx] | -1.03654 | 0.009315 |
| DENOEST_v1_1346 | ID:63768570 suhB\| inositol monophosphatase [Denitratisoma oestradiolicum Fahrbach xx] | -0.58873 | 0.028402 |
| DENOEST_v1_1344 | ID:63768568 fpr\| ferredoxin--NADP+ reductase [Denitratisoma oestradiolicum Fahrbach xx] | -25.7251 | 0.000128 |
| DENOEST_v1_1335 | ID:63768559 sspA\| stringent starvation protein A [Denitratisoma oestradiolicum Fahrbach xx] | -0.77732 | 0.001855 |
| DENOEST_v1_1334 | ID:63768558 petC\| Ammonia monooxygenase gamma subunit [Denitratisoma oestradiolicum Fahrbach xx] | -0.93401 | 0.009626 |
| DENOEST_v1_1332 | ID:63768556 petA\| Ubiquinol-cytochrome c reductase iron-sulfur subunit [Denitratisoma oestradiolicum Fahrbach xx] | -0.79694 | 0.0006 |
| DENOEST_v1_1331 | ID:63768555 Succinyl-CoA--D-citramalate CoA-transferase [Denitratisoma oestradiolicum Fahrbach xx] | -0.90981 | 0.013774 |
| DENOEST_v1_1328 | ID:63768552 dnaX\| DNA polymerase III subunit gamma/tau [Denitratisoma oestradiolicum Fahrbach xx] | -0.79602 | 0.005397 |
| DENOEST_v1_1325 | ID:63768549 Phosphate transport regulator [Denitratisoma oestradiolicum Fahrbach xx] | -0.4557 | 0.041441 |
| DENOEST_v1_1324 | ID:63768548 conserved protein of unknown function [Denitratisoma oestradiolicum Fahrbach xx] | -0.71234 | 0.00722 |
| DENOEST_v1_1319 | ID:63768543 putative parvulin-type peptidyl-prolyl cis-trans isomerase [Denitratisoma oestradiolicum Fahrbach xx] | -0.29632 | 0.047456 |
| DENOEST_v1_1309 | ID:63768533 rpoS\| RNA polymerase, sigma S (sigma 38) factor [Denitratisoma oestradiolicum Fahrbach xx] | 0.23226 | 0.041594 |
| DENOEST_v1_1308 | ID:63768532 Lipoprotein NlpD/LppB homolog [Denitratisoma oestradiolicum Fahrbach xx] | 0.323783 | 0.034678 |
| DENOEST_v1_1305 | ID:63768529 htpG\| molecular chaperone HSP90 family [Denitratisoma oestradiolicum Fahrbach xx] | -0.29878 | 0.005695 |
| DENOEST_v1_1304 | ID:63768528 gst\| glutathionine S-transferase [Denitratisoma oestradiolicum Fahrbach xx] | -0.36658 | 0.0333 |
| DENOEST_v1_1300 | ID:63768524 tig\| Trigger factor [Denitratisoma oestradiolicum Fahrbach xx] | -1.17176 | 0.002212 |
| DENOEST_v1_1299 | ID:63768523 clpP\| ATP-dependent Clp protease proteolytic subunit (Endopeptidase Clp) (Caseinolytic protease) (Protease Ti) (Heat shock protein F21.5) [Denitratisoma oestradiolicum Fahrbach xx] | -0.32199 | 0.026931 |
| DENOEST_v1_1298 | ID:63768522 clpX\| ATP-dependent Clp protease ATP-binding subunit [Denitratisoma oestradiolicum Fahrbach xx] | -0.72303 | 0.000156 |
| DENOEST_v1_1297 | ID:63768521 lon\| DNA-binding ATP-dependent protease La [Denitratisoma oestradiolicum Fahrbach xx] | -0.86784 | 0.022638 |
| DENOEST_v1_1294 | ID:63768518 Aldo/keto reductase [Denitratisoma oestradiolicum Fahrbach xx] | -0.37549 | 0.00183 |
| DENOEST_v1_1293 | ID:63768517 putative TonB-dependent receptor [Denitratisoma oestradiolicum Fahrbach xx] | 0.816443 | 0.000672 |
| DENOEST_v1_1291 | ID:63768515 putative PCP degradation transcriptional activation protein [Denitratisoma oestradiolicum Fahrbach xx] | 0.326158 | 0.04589 |
| DENOEST_v1_1287 | ID:63768511 conserved protein of unknown function [Denitratisoma oestradiolicum Fahrbach xx] | 1.68003 | 0.028445 |
| DENOEST_v1_1277 | ID:63768501 gcdH\| glutaryl-CoA dehydrogenase [Denitratisoma oestradiolicum Fahrbach xx] | 0.923987 | 0.0013 |
| DENOEST_v1_1275 | ID:63768499 Outer membrane lipoprotein-sorting protein [Denitratisoma oestradiolicum Fahrbach xx] | 1.168358 | 0.000517 |
| DENOEST_v1_1274 | ID:63768498 LysR family transcriptional regulator [Denitratisoma oestradiolicum Fahrbach xx] | 1.093463 | 0.001511 |
| DENOEST_v1_1266 | ID:63768490 conserved protein of unknown function [Denitratisoma oestradiolicum Fahrbach xx] | 17.60741 | 0.043133 |
| DENOEST_v1_1264 | ID:63768488 conserved protein of unknown function [Denitratisoma oestradiolicum Fahrbach xx] | 2.045641 | 0.008045 |
| DENOEST_v1_1263 | ID:63768487 conserved protein of unknown function [Denitratisoma oestradiolicum Fahrbach xx] | 16.38647 | 0.001191 |
| DENOEST_v1_1262 | ID:63768486 conserved protein of unknown function [Denitratisoma oestradiolicum Fahrbach xx] | 1.588938 | 0.001354 |
| DENOEST_v1_1259 | ID:63768483 Filamentation induced by cAMP protein Fic [Denitratisoma oestradiolicum Fahrbach xx] | 0.321545 | 0.038812 |
| DENOEST_v1_1252 | ID:63768476 fdhD\| Sulfur carrier protein FdhD [Denitratisoma oestradiolicum Fahrbach xx] | -1.31157 | 0.043947 |
| DENOEST_v1_1250 | ID:63768474 nrfC\| formate-dependent nitrite reductase, 4Fe4S subunit [Denitratisoma oestradiolicum Fahrbach xx] | -1.52157 | 0.000502 |
| DENOEST_v1_1249 | ID:63768473 Molybdopterin oxidoreductase, molybdopterin binding subunit [Denitratisoma oestradiolicum Fahrbach xx] | -1.39655 | 2.78E-05 |
| DENOEST_v1_1247 | ID:63768471 dmsB\| putative dimethyl sulfoxide reductase iron-sulfur subunit B [Denitratisoma oestradiolicum Fahrbach xx] | 2.294731 | 0.00547 |
| DENOEST_v1_1246 | ID:63768470 Molybdopterin oxidoreductase, molybdopterin binding subunit [Denitratisoma oestradiolicum Fahrbach xx] | 1.706661 | 0.002011 |
| DENOEST_v1_1245 | ID:63768469 conserved protein of unknown function [Denitratisoma oestradiolicum Fahrbach xx] | -1.3873 | 0.000165 |
| DENOEST_v1_1242 | ID:63768466 conserved protein of unknown function [Denitratisoma oestradiolicum Fahrbach xx] | 1.089007 | 5.19E-05 |
| DENOEST_v1_1240 | ID:63768464 conserved protein of unknown function [Denitratisoma oestradiolicum Fahrbach xx] | 2.146627 | 2.91E-05 |
| DENOEST_v1_1233 | ID:63768457 Aldehyde dehydrogenase [Denitratisoma oestradiolicum Fahrbach xx] | 0.886092 | 0.045067 |
| DENOEST_v1_1232 | ID:63768456 Hydrolase [Denitratisoma oestradiolicum Fahrbach xx] | 0.317624 | 0.038832 |
| DENOEST_v1_1229 | ID:63768453 conserved exported protein of unknown function [Denitratisoma oestradiolicum Fahrbach xx] | 0.919609 | 0.001104 |
| DENOEST_v1_1223 | ID:63768447 conserved protein of unknown function [Denitratisoma oestradiolicum Fahrbach xx] | -2.73833 | 0.000317 |
| DENOEST_v1_1222 | ID:63768446 cutC\| Glyceraldehyde dehydrogenase small chain [Denitratisoma oestradiolicum Fahrbach xx] | -1.92538 | 0.000266 |
| DENOEST_v1_1221 | ID:63768445 conserved protein of unknown function [Denitratisoma oestradiolicum Fahrbach xx] | -2.78279 | 0.000577 |
| DENOEST_v1_1220 | ID:63768444 conserved exported protein of unknown function [Denitratisoma oestradiolicum Fahrbach xx] | -1.88398 | 0.000341 |
| DENOEST_v1_1219 | ID:63768443 conserved exported protein of unknown function [Denitratisoma oestradiolicum Fahrbach xx] | -1.85096 | 0.000785 |
| DENOEST_v1_1218 | ID:63768442 conserved membrane protein of unknown function [Denitratisoma oestradiolicum Fahrbach xx] | -2.31729 | 0.009813 |
| DENOEST_v1_1217 | ID:63768441 conserved exported protein of unknown function [Denitratisoma oestradiolicum Fahrbach xx] | -2.5233 | 0.00011 |
| DENOEST_v1_1215 | ID:63768439 conserved protein of unknown function [Denitratisoma oestradiolicum Fahrbach xx] | 1.126905 | 0.000649 |
| DENOEST_v1_1203 | ID:63768427 lipA\| lipoate synthase [Denitratisoma oestradiolicum Fahrbach xx] | 2.17828 | 6.21E-05 |
| DENOEST_v1_1202 | ID:63768426 cobQ\| Cobyric acid synthase [Denitratisoma oestradiolicum Fahrbach xx] | 24.76927 | 3.87E-06 |
| DENOEST_v1_1201 | ID:63768425 ABC transporter related [Denitratisoma oestradiolicum Fahrbach xx] | 23.16329 | 0.000781 |
| DENOEST_v1_1200 | ID:63768424 ABC transporter permease [Denitratisoma oestradiolicum Fahrbach xx] | 15.81566 | 0.005892 |
| DENOEST_v1_1199 | ID:63768423 TonB-dependent receptor [Denitratisoma oestradiolicum Fahrbach xx] | 27.40634 | 1.25E-05 |
| DENOEST_v1_1197 | ID:63768421 conserved protein of unknown function [Denitratisoma oestradiolicum Fahrbach xx] | 18.5449 | 0.000779 |
| DENOEST_v1_1194 | ID:63768418 btuB\| Outer membrane cobalamin translocator [Denitratisoma oestradiolicum Fahrbach xx] | 27.2907 | 4.65E-07 |
| DENOEST_v1_1192 | ID:63768416 cobO\| Cob(I)yrinic acid a,c-diamide adenosyltransferase [Denitratisoma oestradiolicum Fahrbach xx] | 25.47892 | 6.95E-06 |
| DENOEST_v1_1191 | ID:63768415 cobB\| Hydrogenobyrinate a,c-diamide synthase [Denitratisoma oestradiolicum Fahrbach xx] | 24.93929 | 4.94E-05 |
| DENOEST_v1_1190 | ID:63768414 fbiC\| FO synthase [Denitratisoma oestradiolicum Fahrbach xx] | 18.93158 | 0.000175 |
| DENOEST_v1_1188 | ID:63768412 putative HTH-type transcriptional regulator TtgR [Denitratisoma oestradiolicum Fahrbach xx] | 5.805403 | 0.000168 |
| DENOEST_v1_1187 | ID:63768411 metF\| 5,10-methylenetetrahydrofolate reductase [Denitratisoma oestradiolicum Fahrbach xx] | 25.4624 | 0.000265 |
| DENOEST_v1_1185 | ID:63768409 ahcY\| Adenosylhomocysteinase [Denitratisoma oestradiolicum Fahrbach xx] | 29.24031 | 0.000877 |
| DENOEST_v1_1184 | ID:63768408 metK\| methionine adenosyltransferase 1 [Denitratisoma oestradiolicum Fahrbach xx] | 29.50521 | 0.000123 |
| DENOEST_v1_1183 | ID:63768407 conserved protein of unknown function [Denitratisoma oestradiolicum Fahrbach xx] | 23.34219 | 0.000707 |
| DENOEST_v1_1181 | ID:63768405 conserved exported protein of unknown function [Denitratisoma oestradiolicum Fahrbach xx] | 31.16925 | 0.000106 |
| DENOEST_v1_1180 | ID:63768404 TetR family transcriptional regulator [Denitratisoma oestradiolicum Fahrbach xx] | 22.01202 | 0.002142 |
| DENOEST_v1_1179 | ID:63768403 Long-chain acyl-CoA synthetase [Denitratisoma oestradiolicum Fahrbach xx] | 21.89164 | 0.009727 |
| DENOEST_v1_1178 | ID:63768402 Lipid-transfer protein [Denitratisoma oestradiolicum Fahrbach xx] | 26.24409 | 3.63E-07 |
| DENOEST_v1_1177 | ID:63768401 MaoC like domain-containing protein [Denitratisoma oestradiolicum Fahrbach xx] | 24.84946 | 2.35E-05 |
| DENOEST_v1_1175 | ID:63768399 conserved protein of unknown function [Denitratisoma oestradiolicum Fahrbach xx] | 25.85434 | 0.000611 |
| DENOEST_v1_1174 | ID:63768398 fabG\| 3-oxoacyl-[acyl-carrier-protein] reductase FabG [Denitratisoma oestradiolicum Fahrbach xx] | 28.64758 | 4.42E-05 |
| DENOEST_v1_1173 | ID:63768397 fabG\| 3-oxoacyl-ACP reductase [Denitratisoma oestradiolicum Fahrbach xx] | 27.37279 | 5.9E-06 |
| DENOEST_v1_1172 | ID:63768396 metH\| homocysteine-N5-methyltetrahydrofolate transmethylase, B12-dependent [Denitratisoma oestradiolicum Fahrbach xx] | 28.84808 | 1.26E-07 |
| DENOEST_v1_1171 | ID:63768395 glyA\| serine hydroxymethyltransferase [Denitratisoma oestradiolicum Fahrbach xx] | 27.90357 | 0.000161 |
| DENOEST_v1_1170 | ID:63768394 Methylamine---corrinoid protein Co-methyltransferase [Denitratisoma oestradiolicum Fahrbach xx] | 30.4189 | 2.82E-05 |
| DENOEST_v1_1169 | ID:63768393 conserved protein of unknown function [Denitratisoma oestradiolicum Fahrbach xx] | 28.21276 | 1.08E-05 |
| DENOEST_v1_1168 | ID:63768392 conserved protein of unknown function [Denitratisoma oestradiolicum Fahrbach xx] | 26.16424 | 0.000283 |
| DENOEST_v1_1167 | ID:63768391 conserved protein of unknown function [Denitratisoma oestradiolicum Fahrbach xx] | 27.98712 | 1.58E-06 |
| DENOEST_v1_1166 | ID:63768390 conserved protein of unknown function [Denitratisoma oestradiolicum Fahrbach xx] | 3.490822 | 0.000131 |
| DENOEST_v1_1165 | ID:63768389 Adenosine kinase [Denitratisoma oestradiolicum Fahrbach xx] | 26.30428 | 2.3E-05 |
| DENOEST_v1_1164 | ID:63768388 conserved protein of unknown function [Denitratisoma oestradiolicum Fahrbach xx] | 25.10326 | 6.72E-05 |
| DENOEST_v1_1163 | ID:63768387 conserved protein of unknown function [Denitratisoma oestradiolicum Fahrbach xx] | 7.697166 | 1.78E-05 |
| DENOEST_v1_1161 | ID:63768385 cobT\| Nicotinate-nucleotide--dimethylbenzimidazole phosphoribosyltransferase [Denitratisoma oestradiolicum Fahrbach xx] | 5.594302 | 0.003892 |
| DENOEST_v1_1160 | ID:63768384 cobC\| Threonine-phosphate decarboxylase [Denitratisoma oestradiolicum Fahrbach xx] | 24.50863 | 0.000118 |
| DENOEST_v1_1159 | ID:63768383 Cobalamin-binding protein [Denitratisoma oestradiolicum Fahrbach xx] | 22.94097 | 0.000819 |
| DENOEST_v1_1156 | ID:63768380 conserved protein of unknown function [Denitratisoma oestradiolicum Fahrbach xx] | 4.130621 | 3.3E-05 |
| DENOEST_v1_1155 | ID:63768379 conserved protein of unknown function [Denitratisoma oestradiolicum Fahrbach xx] | 23.20141 | 0.000291 |
| DENOEST_v1_1153 | ID:63768377 conserved protein of unknown function [Denitratisoma oestradiolicum Fahrbach xx] | 18.44775 | 0.010148 |
| DENOEST_v1_1148 | ID:63768372 putative TonB-dependent receptor [Denitratisoma oestradiolicum Fahrbach xx] | 1.023728 | 0.006767 |
| DENOEST_v1_1144 | ID:63768368 Carboxymuconolactone decarboxylase [Denitratisoma oestradiolicum Fahrbach xx] | 4.942254 | 0.000356 |
| DENOEST_v1_1136 | ID:63768360 conserved protein of unknown function [Denitratisoma oestradiolicum Fahrbach xx] | 17.1861 | 0.000463 |
| DENOEST_v1_1132 | ID:63768356 conserved protein of unknown function [Denitratisoma oestradiolicum Fahrbach xx] | 19.06301 | 0.000389 |
| DENOEST_v1_1118 | ID:63768342 conserved protein of unknown function [Denitratisoma oestradiolicum Fahrbach xx] | 5.230773 | 0.00022 |
| DENOEST_v1_1117 | ID:63768341 conserved protein of unknown function [Denitratisoma oestradiolicum Fahrbach xx] | 3.983996 | 0.000329 |
| DENOEST_v1_1116 | ID:63768340 LexA family transcriptional repressor [Denitratisoma oestradiolicum Fahrbach xx] | 4.842201 | 0.006197 |
| DENOEST_v1_1111 | ID:63768335 folD\| Methylenetetrahydrofolate dehydrogenase / Methenyltetrahydrofolate cyclohydrolase [Denitratisoma oestradiolicum Fahrbach xx] | -0.56414 | 0.001096 |
| DENOEST_v1_1106 | ID:63768330 serS\| seryl-tRNA synthetase, also charges selenocysteinyl-tRNA with serine [Denitratisoma oestradiolicum Fahrbach xx] | -0.58608 | 0.003929 |
| DENOEST_v1_1102 | ID:63768326 DNA-binding protein [Denitratisoma oestradiolicum Fahrbach xx] | -1.06767 | 0.00069 |
| DENOEST_v1_1101 | ID:63768325 yjjK\| fused putative transporter subunits of ABC superfamily: ATP-binding components [Denitratisoma oestradiolicum Fahrbach xx] | -0.77056 | 0.000396 |
| DENOEST_v1_1089 | ID:63768313 CoA transferase [Denitratisoma oestradiolicum Fahrbach xx] | -0.57475 | 0.026104 |
| DENOEST_v1_1068 | ID:63768292 conserved protein of unknown function [Denitratisoma oestradiolicum Fahrbach xx] | -0.83412 | 0.03998 |
| DENOEST_v1_1058 | ID:63768282 Alpha-methylacyl-CoA racemase [Denitratisoma oestradiolicum Fahrbach xx] | 0.573441 | 0.00086 |
| DENOEST_v1_1056 | ID:63768280 Acyl-CoA dehydrogenase [Denitratisoma oestradiolicum Fahrbach xx] | 0.471298 | 0.002343 |
| DENOEST_v1_1053 | ID:63768277 3-hydroxyacyl-CoA dehydrogenase [Denitratisoma oestradiolicum Fahrbach xx] | 0.637334 | 0.001868 |
| DENOEST_v1_1052 | ID:63768276 fadA\| putative enzyme [Denitratisoma oestradiolicum Fahrbach xx] | 0.990051 | 0.003024 |
| DENOEST_v1_1035 | ID:63768259 conserved protein of unknown function [Denitratisoma oestradiolicum Fahrbach xx] | -0.83064 | 0.047732 |
| DENOEST_v1_1032 | ID:63768256 conserved protein of unknown function [Denitratisoma oestradiolicum Fahrbach xx] | -3.44358 | 0.0003 |
| DENOEST_v1_1024 | ID:63768248 conserved protein of unknown function [Denitratisoma oestradiolicum Fahrbach xx] | 0.834628 | 0.015881 |
| DENOEST_v1_1023 | ID:63768247 dut\| deoxyuridinetriphosphatase [Denitratisoma oestradiolicum Fahrbach xx] | -0.58792 | 0.007171 |
| DENOEST_v1_1022 | ID:63768246 dfp\| fused 4'-phosphopantothenoylcysteine decarboxylase ; phosphopantothenoylcysteine synthetase, FMN-binding [Denitratisoma oestradiolicum Fahrbach xx] | 0.922897 | 0.004274 |
| DENOEST_v1_1019 | ID:63768243 rpmB\| 50S ribosomal protein L28 [Denitratisoma oestradiolicum Fahrbach xx] | -1.36736 | 0.000116 |
| DENOEST_v1_1018 | ID:63768242 rpmG\| 50S ribosomal protein L33 [Denitratisoma oestradiolicum Fahrbach xx] | -1.89443 | 0.012826 |
| DENOEST_v1_1015 | ID:63768239 S-methyl-5'-thioinosine phosphorylase [Denitratisoma oestradiolicum Fahrbach xx] | -0.3112 | 0.029261 |
| DENOEST_v1_1014 | ID:63768238 bipA\| GTP-binding protein [Denitratisoma oestradiolicum Fahrbach xx] | -1.72238 | 2.37E-05 |
| DENOEST_v1_1012 | ID:63768236 Iron complex outermembrane recepter protein [Denitratisoma oestradiolicum Fahrbach xx] | 0.491742 | 0.001193 |
| DENOEST_v1_1009 | ID:63768233 gtaB\| UTP--glucose-1-phosphate uridylyltransferase [Denitratisoma oestradiolicum Fahrbach xx] | -0.48392 | 0.00939 |
| DENOEST_v1_1006 | ID:63768230 putative Cell division protein ZipA [Denitratisoma oestradiolicum Fahrbach xx] | -0.47373 | 0.003165 |
| DENOEST_v1_1005 | ID:63768229 smc\| Chromosome partition protein Smc [Denitratisoma oestradiolicum Fahrbach xx] | -0.14407 | 0.016835 |
| DENOEST_v1_1004 | ID:63768228 queF\| NADPH-dependent 7-cyano-7-deazaguanine reductase [Denitratisoma oestradiolicum Fahrbach xx] | -0.47801 | 0.002459 |
| DENOEST_v1_0993 | ID:63768217 Metal-dependent phosphohydrolase [Denitratisoma oestradiolicum Fahrbach xx] | -0.67369 | 0.011421 |
| DENOEST_v1_0991 | ID:63768215 GNAT family N-acetyltransferase [Denitratisoma oestradiolicum Fahrbach xx] | 0.60139 | 0.005987 |
| DENOEST_v1_0990 | ID:63768214 tag\| 3-methyl-adenine DNA glycosylase I, constitutive [Denitratisoma oestradiolicum Fahrbach xx] | 0.586133 | 0.018026 |
| DENOEST_v1_0988 | ID:63768212 clpB\| protein disaggregation chaperone [Denitratisoma oestradiolicum Fahrbach xx] | 0.275081 | 0.036291 |
| DENOEST_v1_0987 | ID:63768211 conserved protein of unknown function [Denitratisoma oestradiolicum Fahrbach xx] | 0.309772 | 0.025321 |
| DENOEST_v1_0985 | ID:63768209 moaD\| molybdopterin synthase, small subunit [Denitratisoma oestradiolicum Fahrbach xx] | -2.1423 | 0.015761 |
| DENOEST_v1_0984 | ID:63768208 mobB\| Molybdopterin-guanine dinucleotide biosynthesis adapter protein [Denitratisoma oestradiolicum Fahrbach xx] | -1.46686 | 0.008728 |
| DENOEST_v1_0982 | ID:63768206 putative ATP-dependent DNA helicase HI_0387 [Denitratisoma oestradiolicum Fahrbach xx] | -0.79444 | 0.018249 |
| DENOEST_v1_0980 | ID:63768204 Chemotaxis sensory transducer [Denitratisoma oestradiolicum Fahrbach xx] | -1.32241 | 0.000967 |
| DENOEST_v1_0979 | ID:63768203 amiC\| N-acetylmuramoyl-L-alanine amidase AmiC [Denitratisoma oestradiolicum Fahrbach xx] | -1.65632 | 0.00994 |
| DENOEST_v1_0972 | ID:63768196 ispA\| geranyltranstransferase [Denitratisoma oestradiolicum Fahrbach xx] | -0.28691 | 0.003099 |
| DENOEST_v1_0971 | ID:63768195 dxs\| 1-deoxyxylulose-5-phosphate synthase, thiamine-requiring, FAD-requiring [Denitratisoma oestradiolicum Fahrbach xx] | -0.63273 | 0.019632 |
| DENOEST_v1_0964 | ID:63768188 L516 [Denitratisoma oestradiolicum Fahrbach xx] | -0.36721 | 0.032182 |
| DENOEST_v1_0957 | ID:63768181 nuoI\| NADH-quinone oxidoreductase subunit I [Denitratisoma oestradiolicum Fahrbach xx] | -0.46371 | 0.016351 |
| DENOEST_v1_0955 | ID:63768179 NADH-quinone oxidoreductase [Denitratisoma oestradiolicum Fahrbach xx] | -0.36361 | 0.002345 |
| DENOEST_v1_0954 | ID:63768178 nuoF\| NADH:ubiquinone oxidoreductase, chain F [Denitratisoma oestradiolicum Fahrbach xx] | -0.43761 | 0.023844 |
| DENOEST_v1_0953 | ID:63768177 nqo\| NADH-quinone oxidoreductase subunit 2 [Denitratisoma oestradiolicum Fahrbach xx] | -0.99642 | 0.030306 |
| DENOEST_v1_0952 | ID:63768176 nuoD\| NADH-quinone oxidoreductase subunit D [Denitratisoma oestradiolicum Fahrbach xx] | -0.40118 | 0.01102 |
| DENOEST_v1_0951 | ID:63768175 nuoC\| NADH-quinone oxidoreductase subunit C [Denitratisoma oestradiolicum Fahrbach xx] | -0.54583 | 0.008638 |
| DENOEST_v1_0950 | ID:63768174 nuoB\| NADH-quinone oxidoreductase subunit B [Denitratisoma oestradiolicum Fahrbach xx] | -0.36624 | 0.045799 |
| DENOEST_v1_0947 | ID:63768171 tpiA\| Triosephosphate isomerase [Denitratisoma oestradiolicum Fahrbach xx] | -0.46651 | 0.000383 |
| DENOEST_v1_0945 | ID:63768169 Indolepyruvate ferredoxin oxidoreductase [Denitratisoma oestradiolicum Fahrbach xx] | 1.549573 | 0.003679 |
| DENOEST_v1_0944 | ID:63768168 Diguanylate cyclase/phosphodiesterase [Denitratisoma oestradiolicum Fahrbach xx] | -2.50419 | 3.65E-05 |
| DENOEST_v1_0942 | ID:63768166 yhgF\| transcriptional accessory protein [Denitratisoma oestradiolicum Fahrbach xx] | -2.47508 | 0.028187 |
| DENOEST_v1_0939 | ID:63768163 Spermidine synthase [Denitratisoma oestradiolicum Fahrbach xx] | -2.4012 | 0.015853 |
| DENOEST_v1_0918 | ID:63768142 putative CRISPR-associated protein, Cse3 family [Denitratisoma oestradiolicum Fahrbach xx] | -1.01704 | 0.000187 |
| DENOEST_v1_0917 | ID:63768141 conserved protein of unknown function [Denitratisoma oestradiolicum Fahrbach xx] | -1.13478 | 0.009318 |
| DENOEST_v1_0916 | ID:63768140 CRISPR-associated protein Cas7/Cse4/CasC, subtype I-E [Denitratisoma oestradiolicum Fahrbach xx] | -0.82722 | 0.000346 |
| DENOEST_v1_0915 | ID:63768139 conserved protein of unknown function [Denitratisoma oestradiolicum Fahrbach xx] | -0.57884 | 0.020826 |
| DENOEST_v1_0911 | ID:63768135 conserved protein of unknown function [Denitratisoma oestradiolicum Fahrbach xx] | 1.940209 | 9.37E-05 |
| DENOEST_v1_0910 | ID:63768134 putative phosphoenolpyruvate synthase regulatory protein [Denitratisoma oestradiolicum Fahrbach xx] | 2.029817 | 5.82E-05 |
| DENOEST_v1_0903 | ID:63768127 fabZ\| (3R)-hydroxymyristol acyl carrier protein dehydratase [Denitratisoma oestradiolicum Fahrbach xx] | -1.0307 | 0.030693 |
| DENOEST_v1_0898 | ID:63768122 dxr\| 1-deoxy-D-xylulose 5-phosphate reductoisomerase [Denitratisoma oestradiolicum Fahrbach xx] | -1.08238 | 0.00178 |
| DENOEST_v1_0895 | ID:63768119 frr\| ribosome releasing factor [Denitratisoma oestradiolicum Fahrbach xx] | -1.08821 | 0.002119 |
| DENOEST_v1_0894 | ID:63768118 pyrH\| uridylate kinase [Denitratisoma oestradiolicum Fahrbach xx] | -0.90318 | 0.002748 |
| DENOEST_v1_0893 | ID:63768117 tsf\| protein chain elongation factor EF-Ts [Denitratisoma oestradiolicum Fahrbach xx] | -1.26301 | 0.00022 |
| DENOEST_v1_0892 | ID:63768116 rpsB\| 30S ribosomal protein S2 [Denitratisoma oestradiolicum Fahrbach xx] | -0.97399 | 0.001737 |
| DENOEST_v1_0884 | ID:63768108 conserved exported protein of unknown function [Denitratisoma oestradiolicum Fahrbach xx] | 0.242934 | 0.024206 |
| DENOEST_v1_0877 | ID:63768101 conserved protein of unknown function [Denitratisoma oestradiolicum Fahrbach xx] | 1.339042 | 0.000312 |
| DENOEST_v1_0875 | ID:63768099 conserved exported protein of unknown function [Denitratisoma oestradiolicum Fahrbach xx] | 0.332239 | 0.001878 |
| DENOEST_v1_0873 | ID:63768097 ispH\| 1-hydroxy-2-methyl-2-(E)-butenyl 4-diphosphate reductase, 4Fe-4S protein [Denitratisoma oestradiolicum Fahrbach xx] | -0.39033 | 0.049614 |
| DENOEST_v1_0872 | ID:63768096 Peptidyl-prolyl cis-trans isomerase [Denitratisoma oestradiolicum Fahrbach xx] | -1.74283 | 0.001683 |
| DENOEST_v1_0870 | ID:63768094 ileS\| isoleucyl-tRNA synthetase [Denitratisoma oestradiolicum Fahrbach xx] | -1.3181 | 0.035188 |
| DENOEST_v1_0869 | ID:63768093 ribF\| bifunctional riboflavin kinase and FAD synthetase [Denitratisoma oestradiolicum Fahrbach xx] | -0.67395 | 0.003 |
| DENOEST_v1_0855 | ID:63768079 RND family efflux transporter, MFP subunit (fragment) [Denitratisoma oestradiolicum Fahrbach xx] | 1.930951 | 0.000612 |
| DENOEST_v1_0839 | ID:63768063 putative TonB-dependent receptor [Denitratisoma oestradiolicum Fahrbach xx] | 15.55976 | 0.009598 |
| DENOEST_v1_0837 | ID:63768061 otsB\| trehalose-6-phosphate phosphatase, biosynthetic [Denitratisoma oestradiolicum Fahrbach xx] | -1.09421 | 5.49E-05 |
| DENOEST_v1_0831 | ID:63768055 putative glutamate--cysteine ligase 2-1 [Denitratisoma oestradiolicum Fahrbach xx] | -0.52796 | 0.009215 |
| DENOEST_v1_0829 | ID:63768053 putative OmpA family protein [Denitratisoma oestradiolicum Fahrbach xx] | -0.62446 | 0.044276 |
| DENOEST_v1_0828 | ID:63768052 Transcriptional regulator, MarR family [Denitratisoma oestradiolicum Fahrbach xx] | -0.90414 | 0.007992 |
| DENOEST_v1_0827 | ID:63768051 conserved exported protein of unknown function [Denitratisoma oestradiolicum Fahrbach xx] | -0.93878 | 0.007095 |
| DENOEST_v1_0819 | ID:63768043 maeB\| putative fused malic enzyme oxidoreductase ; putative phosphotransacetylase [Denitratisoma oestradiolicum Fahrbach xx] | -0.33411 | 0.016912 |
| DENOEST_v1_0812 | ID:63768036 ispD\| 4-diphosphocytidyl-2C-methyl-D-erythritol synthase [Denitratisoma oestradiolicum Fahrbach xx] | -0.47876 | 0.024336 |
| DENOEST_v1_0807 | ID:63768031 conserved exported protein of unknown function [Denitratisoma oestradiolicum Fahrbach xx] | 0.363147 | 0.002185 |
| DENOEST_v1_0805 | ID:63768029 rnr\| Ribonuclease R [Denitratisoma oestradiolicum Fahrbach xx] | -0.26986 | 0.012249 |
| DENOEST_v1_0802 | ID:63768026 folK\| 2-amino-4-hydroxy-6-hydroxymethyldihyropteridine pyrophosphokinase [Denitratisoma oestradiolicum Fahrbach xx] | -0.88705 | 0.014408 |
| DENOEST_v1_0799 | ID:63768023 panC\| pantothenate synthetase [Denitratisoma oestradiolicum Fahrbach xx] | -0.37326 | 0.024295 |
| DENOEST_v1_0798 | ID:63768022 panD\| aspartate 1-decarboxylase [Denitratisoma oestradiolicum Fahrbach xx] | 3.895423 | 0.000343 |
| DENOEST_v1_0795 | ID:63768019 narG\| nitrate reductase 1, alpha subunit [Denitratisoma oestradiolicum Fahrbach xx] | -1.30778 | 5.56E-05 |
| DENOEST_v1_0794 | ID:63768018 narY\| nitrate reductase 2 (NRZ), beta subunit [Denitratisoma oestradiolicum Fahrbach xx] | -1.58302 | 0.000187 |
| DENOEST_v1_0793 | ID:63768017 narJ\| Chaperone [Denitratisoma oestradiolicum Fahrbach xx] | -4.2337 | 7.9E-06 |
| DENOEST_v1_0790 | ID:63768014 narL\| DNA-binding response regulator in two-component regulatory system with NarX (or NarQ) [Denitratisoma oestradiolicum Fahrbach xx] | -0.82793 | 0.004053 |
| DENOEST_v1_0785 | ID:63768009 Protein TonB [Denitratisoma oestradiolicum Fahrbach xx] | 1.229949 | 0.008151 |
| DENOEST_v1_0784 | ID:63768008 exbB\| Biopolymer transport protein ExbB [Denitratisoma oestradiolicum Fahrbach xx] | 0.605371 | 0.024518 |
| DENOEST_v1_0783 | ID:63768007 Outer membrane transport energization protein ExbD [Denitratisoma oestradiolicum Fahrbach xx] | 0.522871 | 0.035902 |
| DENOEST_v1_0782 | ID:63768006 Biopolymer transporter ExbD [Denitratisoma oestradiolicum Fahrbach xx] | 0.87613 | 0.021331 |
| DENOEST_v1_0781 | ID:63768005 conserved exported protein of unknown function [Denitratisoma oestradiolicum Fahrbach xx] | 0.69677 | 0.000496 |
| DENOEST_v1_0780 | ID:63768004 Short chain dehydrogenase [Denitratisoma oestradiolicum Fahrbach xx] | 0.404867 | 0.000986 |
| DENOEST_v1_0779 | ID:63768003 putative Luciferase family protein [Denitratisoma oestradiolicum Fahrbach xx] | 1.859495 | 0.000352 |
| DENOEST_v1_0773 | ID:63767997 conserved protein of unknown function [Denitratisoma oestradiolicum Fahrbach xx] | -0.31354 | 0.004421 |
| DENOEST_v1_0761 | ID:63767985 putative TonB-dependent receptor [Denitratisoma oestradiolicum Fahrbach xx] | 1.79208 | 0.012877 |
| DENOEST_v1_0752 | ID:63767976 metZ\| O-succinylhomoserine sulfhydrylase [Denitratisoma oestradiolicum Fahrbach xx] | -0.59398 | 0.002116 |
| DENOEST_v1_0751 | ID:63767975 purF\| amidophosphoribosyltransferase [Denitratisoma oestradiolicum Fahrbach xx] | -0.71957 | 0.001583 |
| DENOEST_v1_0747 | ID:63767971 accD\| acetyl-CoA carboxylase, beta (carboxyltranferase) subunit [Denitratisoma oestradiolicum Fahrbach xx] | -1.11525 | 0.002028 |
| DENOEST_v1_0746 | ID:63767970 trpA\| Tryptophan synthase alpha chain [Denitratisoma oestradiolicum Fahrbach xx] | -0.60627 | 3.72E-05 |
| DENOEST_v1_0745 | ID:63767969 trpB\| tryptophan synthase beta chain [Denitratisoma oestradiolicum Fahrbach xx] | -0.82128 | 7.06E-05 |
| DENOEST_v1_0744 | ID:63767968 trpF\| N-(5'-phosphoribosyl)anthranilate isomerase [Denitratisoma oestradiolicum Fahrbach xx] | -0.97041 | 0.004912 |
| DENOEST_v1_0740 | ID:63767964 asd\| aspartate-semialdehyde dehydrogenase, NAD(P)-binding [Denitratisoma oestradiolicum Fahrbach xx] | -0.48499 | 0.015382 |
| DENOEST_v1_0739 | ID:63767963 leuB\| 3-isopropylmalate dehydrogenase [Denitratisoma oestradiolicum Fahrbach xx] | -0.77436 | 1.06E-05 |
| DENOEST_v1_0738 | ID:63767962 leuD\| 3-isopropylmalate isomerase (dehydratase), subunit with LeuC [Denitratisoma oestradiolicum Fahrbach xx] | -0.75534 | 0.001319 |
| DENOEST_v1_0737 | ID:63767961 leuC\| 3-isopropylmalate dehydratase (isomerase), subunit with LeuD [Denitratisoma oestradiolicum Fahrbach xx] | -0.73293 | 0.002837 |
| DENOEST_v1_0732 | ID:63767956 CBS domain-containing protein [Denitratisoma oestradiolicum Fahrbach xx] | 0.666306 | 0.033751 |
| DENOEST_v1_0730 | ID:63767954 trxA\| thioredoxin 1 [Denitratisoma oestradiolicum Fahrbach xx] | -0.67552 | 0.017757 |
| DENOEST_v1_0729 | ID:63767953 rho\| transcription termination factor [Denitratisoma oestradiolicum Fahrbach xx] | -0.5425 | 0.002097 |
| DENOEST_v1_0723 | ID:63767947 atoB\| acetyl-CoA acetyltransferase [Denitratisoma oestradiolicum Fahrbach xx] | -1.53786 | 4.34E-05 |
| DENOEST_v1_0721 | ID:63767945 conserved protein of unknown function [Denitratisoma oestradiolicum Fahrbach xx] | -0.73054 | 0.00974 |
| DENOEST_v1_0718 | ID:63767942 ppnP\| Pyrimidine/purine nucleoside phosphorylase [Denitratisoma oestradiolicum Fahrbach xx] | -0.91376 | 0.000961 |
| DENOEST_v1_0716 | ID:63767940 argG\| Argininosuccinate synthase [Denitratisoma oestradiolicum Fahrbach xx] | -0.77972 | 5.19E-05 |
| DENOEST_v1_0714 | ID:63767938 argD\| Acetylornithine aminotransferase [Denitratisoma oestradiolicum Fahrbach xx] | -0.22892 | 0.022371 |
| DENOEST_v1_0712 | ID:63767936 rmlC\| dTDP-4-deoxyrhamnose-3,5-epimerase [Denitratisoma oestradiolicum Fahrbach xx] | -0.80187 | 0.012372 |
| DENOEST_v1_0705 | ID:63767929 hisD\| bifunctional histidinal dehydrogenase and histidinol dehydrogenase [Denitratisoma oestradiolicum Fahrbach xx] | -0.26168 | 0.003936 |
| DENOEST_v1_0704 | ID:63767928 hisG\| ATP phosphoribosyltransferase [Denitratisoma oestradiolicum Fahrbach xx] | -0.38497 | 0.001279 |
| DENOEST_v1_0698 | ID:63767922 conserved exported protein of unknown function [Denitratisoma oestradiolicum Fahrbach xx] | -0.56007 | 0.010845 |
| DENOEST_v1_0697 | ID:63767921 ABC transporter [Denitratisoma oestradiolicum Fahrbach xx] | -0.58234 | 0.000368 |
| DENOEST_v1_0696 | ID:63767920 yrbD\| toluene transporter subunit: membrane component of ABC superfamily [Denitratisoma oestradiolicum Fahrbach xx] | -0.60538 | 0.009566 |
| DENOEST_v1_0694 | ID:63767918 yrbF\| toluene transporter subunit: ATP-binding component of ABC superfamily [Denitratisoma oestradiolicum Fahrbach xx] | -0.9102 | 0.000659 |
| DENOEST_v1_0688 | ID:63767912 Sulfite reductase [Denitratisoma oestradiolicum Fahrbach xx] | -1.64027 | 1.87E-05 |
| DENOEST_v1_0687 | ID:63767911 conserved protein of unknown function [Denitratisoma oestradiolicum Fahrbach xx] | -2.03196 | 0.000733 |
| DENOEST_v1_0686 | ID:63767910 cysH\| Thioredoxin-dependent 5'-adenylylsulfate reductase [Denitratisoma oestradiolicum Fahrbach xx] | -1.51152 | 0.009372 |
| DENOEST_v1_0683 | ID:63767907 putative TonB-dependent receptor [Denitratisoma oestradiolicum Fahrbach xx] | 0.950389 | 0.00175 |
| DENOEST_v1_0681 | ID:63767905 Type I secretion protein ATPase [Denitratisoma oestradiolicum Fahrbach xx] | 0.667701 | 0.003587 |
| DENOEST_v1_0679 | ID:63767903 glnS\| glutamyl-tRNA synthetase [Denitratisoma oestradiolicum Fahrbach xx] | -0.67582 | 0.000616 |
| DENOEST_v1_0678 | ID:63767902 pyrF\| Orotidine 5'-phosphate decarboxylase [Denitratisoma oestradiolicum Fahrbach xx] | -0.58164 | 0.016602 |
| DENOEST_v1_0675 | ID:63767899 Metal dependent phosphohydrolase [Denitratisoma oestradiolicum Fahrbach xx] | 0.99064 | 0.004185 |
| DENOEST_v1_0671 | ID:63767895 deaD\| ATP-dependent RNA helicase DeaD [Denitratisoma oestradiolicum Fahrbach xx] | -0.90866 | 0.000358 |
| DENOEST_v1_0661 | ID:63767885 fbp\| Fructose-1,6-bisphosphatase class 1 1 [Denitratisoma oestradiolicum Fahrbach xx] | -0.25367 | 0.022811 |
| DENOEST_v1_0658 | ID:63767882 Diguanylate cyclase/phosphodiesterase with PAS/PAC sensor [Denitratisoma oestradiolicum Fahrbach xx] | 0.19298 | 0.030542 |
| DENOEST_v1_0654 | ID:63767878 cobT\| Nicotinate-nucleotide--dimethylbenzimidazole phosphoribosyltransferase [Denitratisoma oestradiolicum Fahrbach xx] | 2.109013 | 6.42E-06 |
| DENOEST_v1_0653 | ID:63767877 Cobalamin-binding protein [Denitratisoma oestradiolicum Fahrbach xx] | 2.759885 | 2.99E-05 |
| DENOEST_v1_0652 | ID:63767876 Periplasmic binding protein [Denitratisoma oestradiolicum Fahrbach xx] | 2.041901 | 0.000416 |
| DENOEST_v1_0650 | ID:63767874 conserved protein of unknown function [Denitratisoma oestradiolicum Fahrbach xx] | 2.849536 | 0.017286 |
| DENOEST_v1_0647 | ID:63767871 prfB\| Peptide chain release factor 2 [Denitratisoma oestradiolicum Fahrbach xx] | -0.72166 | 0.003132 |
| DENOEST_v1_0646 | ID:63767870 prfC\| peptide chain release factor RF-3 [Denitratisoma oestradiolicum Fahrbach xx] | -0.63574 | 0.0023 |
| DENOEST_v1_0640 | ID:63767864 Acetolactate synthase [Denitratisoma oestradiolicum Fahrbach xx] | 0.657592 | 0.004886 |
| DENOEST_v1_0637 | ID:63767861 lysU\| lysine tRNA synthetase, inducible [Denitratisoma oestradiolicum Fahrbach xx] | -0.68439 | 5.72E-05 |
| DENOEST_v1_0634 | ID:63767858 gltX\| glutamyl-tRNA synthetase [Denitratisoma oestradiolicum Fahrbach xx] | -0.50648 | 0.003367 |
| DENOEST_v1_0622 | ID:63767846 Deaminated glutathione amidase [Denitratisoma oestradiolicum Fahrbach xx] | -0.42829 | 0.016286 |
| DENOEST_v1_0620 | ID:63767844 tldD\| putative peptidase [Denitratisoma oestradiolicum Fahrbach xx] | -0.90897 | 0.00015 |
| DENOEST_v1_0616 | ID:63767840 rpe\| D-ribulose-5-phosphate 3-epimerase [Denitratisoma oestradiolicum Fahrbach xx] | -0.41248 | 0.01749 |
| DENOEST_v1_0614 | ID:63767838 trpE\| Anthranilate synthase component 1 [Denitratisoma oestradiolicum Fahrbach xx] | -0.77629 | 0.007528 |
| DENOEST_v1_0613 | ID:63767837 conserved exported protein of unknown function [Denitratisoma oestradiolicum Fahrbach xx] | 1.429138 | 0.000311 |
| DENOEST_v1_0612 | ID:63767836 thiC\| hydroxymethylpyrimidine moiety synthesis in thiamin biosynthesis [Denitratisoma oestradiolicum Fahrbach xx] | -0.43483 | 0.006959 |
| DENOEST_v1_0610 | ID:63767834 conserved exported protein of unknown function [Denitratisoma oestradiolicum Fahrbach xx] | 1.371029 | 2.59E-05 |
| DENOEST_v1_0609 | ID:63767833 aceA\| isocitrate lyase [Denitratisoma oestradiolicum Fahrbach xx] | -7.89807 | 6.4E-07 |
| DENOEST_v1_0607 | ID:63767831 rhlE\| RNA helicase [Denitratisoma oestradiolicum Fahrbach xx] | -2.47233 | 0.012394 |
| DENOEST_v1_0606 | ID:63767830 clpA\| ATP-binding protease component [Denitratisoma oestradiolicum Fahrbach xx] | -1.05571 | 0.002371 |
| DENOEST_v1_0602 | ID:63767826 icd\| isocitrate dehydrogenase [Denitratisoma oestradiolicum Fahrbach xx] | 0.391961 | 0.010181 |
| DENOEST_v1_0600 | ID:63767824 idh\| isocitrate dehydrogenase [Denitratisoma oestradiolicum Fahrbach xx] | -3.90601 | 0.000155 |
| DENOEST_v1_0599 | ID:63767823 aceK\| Isocitrate dehydrogenase kinase/phosphatase [Denitratisoma oestradiolicum Fahrbach xx] | -2.94282 | 0.005041 |
| DENOEST_v1_0597 | ID:63767821 cphA\| Cyanophycin synthetase [Denitratisoma oestradiolicum Fahrbach xx] | -0.44273 | 1.94E-05 |
| DENOEST_v1_0596 | ID:63767820 Cyanophycin synthetase [Denitratisoma oestradiolicum Fahrbach xx] | -0.57729 | 0.001226 |
| DENOEST_v1_0594 | ID:63767818 conserved protein of unknown function [Denitratisoma oestradiolicum Fahrbach xx] | -0.57728 | 0.039619 |
| DENOEST_v1_0592 | ID:63767816 NAD(P)H-quinone oxidoreductase [Denitratisoma oestradiolicum Fahrbach xx] | -0.65306 | 0.005061 |
| DENOEST_v1_0591 | ID:63767815 purC\| Phosphoribosylaminoimidazole-succinocarboxamide synthase [Denitratisoma oestradiolicum Fahrbach xx] | -0.23476 | 0.02728 |
| DENOEST_v1_0589 | ID:63767813 prlC\| Oligopeptidase A [Denitratisoma oestradiolicum Fahrbach xx] | -0.4586 | 0.004871 |
| DENOEST_v1_0586 | ID:63767810 Phosphoglycerate/bisphosphoglycerate mutase [Denitratisoma oestradiolicum Fahrbach xx] | 18.02492 | 0.000128 |
| DENOEST_v1_0584 | ID:63767808 cobP\| Bifunctional adenosylcobalamin biosynthesis protein CobP [Denitratisoma oestradiolicum Fahrbach xx] | 2.741454 | 0.000175 |
| DENOEST_v1_0578 | ID:63767802 pyrE\| orotate phosphoribosyltransferase [Denitratisoma oestradiolicum Fahrbach xx] | -0.25476 | 0.004197 |
| DENOEST_v1_0577 | ID:63767801 conserved exported protein of unknown function [Denitratisoma oestradiolicum Fahrbach xx] | 0.721013 | 0.002221 |
| DENOEST_v1_0576 | ID:63767800 gatB\| aspartyl/glutamyl-tRNA(Asn/Gln) amidotransferase subunit B [Denitratisoma oestradiolicum Fahrbach xx] | -0.57852 | 0.003707 |
| DENOEST_v1_0575 | ID:63767799 gatA\| aspartyl/glutamyl-tRNA(Asn/Gln) amidotransferase subunit A [Denitratisoma oestradiolicum Fahrbach xx] | -0.56873 | 0.023816 |
| DENOEST_v1_0573 | ID:63767797 mreB\| rod shape-determining protein [Denitratisoma oestradiolicum Fahrbach xx] | -0.55684 | 0.003608 |
| DENOEST_v1_0568 | ID:63767792 mrdB\| cell wall shape-determining protein [Denitratisoma oestradiolicum Fahrbach xx] | 2.611729 | 0.000106 |
| DENOEST_v1_0561 | ID:63767785 ccmE\| periplasmic heme chaperone [Denitratisoma oestradiolicum Fahrbach xx] | -0.57878 | 0.010007 |
| DENOEST_v1_0549 | ID:63767773 Cytochrome C [Denitratisoma oestradiolicum Fahrbach xx] | 0.432579 | 0.042925 |
| DENOEST_v1_0546 | ID:63767770 Peptidylprolyl isomerase [Denitratisoma oestradiolicum Fahrbach xx] | 0.154771 | 0.025451 |
| DENOEST_v1_0545 | ID:63767769 conserved exported protein of unknown function [Denitratisoma oestradiolicum Fahrbach xx] | 0.604442 | 0.02709 |
| DENOEST_v1_0544 | ID:63767768 6-bladed beta-propeller [Denitratisoma oestradiolicum Fahrbach xx] | 0.332365 | 0.0007 |
| DENOEST_v1_0537 | ID:63767761 conserved protein of unknown function [Denitratisoma oestradiolicum Fahrbach xx] | 0.424565 | 0.040316 |
| DENOEST_v1_0525 | ID:63767749 conserved exported protein of unknown function [Denitratisoma oestradiolicum Fahrbach xx] | -0.28182 | 0.003178 |
| DENOEST_v1_0524 | ID:63767748 conserved exported protein of unknown function [Denitratisoma oestradiolicum Fahrbach xx] | -0.48326 | 0.005219 |
| DENOEST_v1_0521 | ID:63767745 ccmG\| periplasmic thioredoxin of cytochrome c-type biogenesis [Denitratisoma oestradiolicum Fahrbach xx] | -0.913 | 0.004556 |
| DENOEST_v1_0519 | ID:63767743 ccmI\| C-type cytochrome biogenesis protein CcmI [Denitratisoma oestradiolicum Fahrbach xx] | -0.64933 | 0.004307 |
| DENOEST_v1_0511 | ID:63767735 Regulator [Denitratisoma oestradiolicum Fahrbach xx] | 0.685113 | 0.042784 |
| DENOEST_v1_0510 | ID:63767734 Regulator [Denitratisoma oestradiolicum Fahrbach xx] | 0.655515 | 0.000185 |
| DENOEST_v1_0507 | ID:63767731 thiE\| Thiamine-phosphate synthase [Denitratisoma oestradiolicum Fahrbach xx] | -0.73057 | 0.001779 |
| DENOEST_v1_0506 | ID:63767730 Bifunctional hydroxymethylpyrimidine kinase/phosphomethylpyrimidine kinase [Denitratisoma oestradiolicum Fahrbach xx] | -0.49785 | 0.001783 |
| DENOEST_v1_0504 | ID:63767728 conserved protein of unknown function [Denitratisoma oestradiolicum Fahrbach xx] | 0.922642 | 0.011174 |
| DENOEST_v1_0503 | ID:63767727 conserved protein of unknown function [Denitratisoma oestradiolicum Fahrbach xx] | 1.408572 | 0.000212 |
| DENOEST_v1_0502 | ID:63767726 Alcohol dehydrogenase [Denitratisoma oestradiolicum Fahrbach xx] | 1.371835 | 0.001389 |
| DENOEST_v1_0501 | ID:63767725 Short-chain dehydrogenase [Denitratisoma oestradiolicum Fahrbach xx] | 1.675267 | 0.008786 |
| DENOEST_v1_0500 | ID:63767724 Luciferase [Denitratisoma oestradiolicum Fahrbach xx] | 2.24713 | 0.025686 |
| DENOEST_v1_0498 | ID:63767722 Oxidoreductase [Denitratisoma oestradiolicum Fahrbach xx] | 1.6217 | 0.021547 |
| DENOEST_v1_0491 | ID:63767715 conserved protein of unknown function [Denitratisoma oestradiolicum Fahrbach xx] | 17.78627 | 0.001943 |
| DENOEST_v1_0490 | ID:63767714 qbdA\| Quinohemoprotein alcohol dehydrogenase ADH IIB [Denitratisoma oestradiolicum Fahrbach xx] | 16.57941 | 0.009997 |
| DENOEST_v1_0489 | ID:63767713 2,4-dienoyl-CoA reductase [Denitratisoma oestradiolicum Fahrbach xx] | 0.561628 | 0.006351 |
| DENOEST_v1_0486 | ID:63767710 conserved protein of unknown function [Denitratisoma oestradiolicum Fahrbach xx] | 0.781265 | 0.020747 |
| DENOEST_v1_0485 | ID:63767709 Fumarate reductase [Denitratisoma oestradiolicum Fahrbach xx] | 0.621347 | 0.019327 |
| DENOEST_v1_0478 | ID:63767702 rplI\| 50S ribosomal subunit protein L9 [Denitratisoma oestradiolicum Fahrbach xx] | -1.18922 | 0.003261 |
| DENOEST_v1_0477 | ID:63767701 rpsR\| 30S ribosomal protein S18 [Denitratisoma oestradiolicum Fahrbach xx] | -1.21719 | 0.021244 |
| DENOEST_v1_0475 | ID:63767699 rpsF\| 30S ribosomal subunit protein S6 [Denitratisoma oestradiolicum Fahrbach xx] | -0.82467 | 0.035954 |
| DENOEST_v1_0474 | ID:63767698 rplU\| 50S ribosomal protein L21 [Denitratisoma oestradiolicum Fahrbach xx] | -0.85913 | 0.027244 |
| DENOEST_v1_0473 | ID:63767697 rpmA\| 50S ribosomal protein L27 [Denitratisoma oestradiolicum Fahrbach xx] | -1.39991 | 0.002118 |
| DENOEST_v1_0472 | ID:63767696 putative GTP-binding protein (Obg) [Denitratisoma oestradiolicum Fahrbach xx] | -0.86535 | 0.00168 |
| DENOEST_v1_0471 | ID:63767695 proB\| Glutamate 5-kinase [Denitratisoma oestradiolicum Fahrbach xx] | -0.49448 | 0.004447 |
| DENOEST_v1_0467 | ID:63767691 conserved protein of unknown function [Denitratisoma oestradiolicum Fahrbach xx] | 0.612994 | 0.004991 |
| DENOEST_v1_0465 | ID:63767689 conserved protein of unknown function [Denitratisoma oestradiolicum Fahrbach xx] | 0.600566 | 0.036499 |
| DENOEST_v1_0462 | ID:63767686 Uncharacterized monothiol glutaredoxin ycf64-like [Denitratisoma oestradiolicum Fahrbach xx] | -1.61996 | 0.000411 |
| DENOEST_v1_0457 | ID:63767681 prfA\| peptide chain release factor RF-1 [Denitratisoma oestradiolicum Fahrbach xx] | -0.75824 | 0.044586 |
| DENOEST_v1_0456 | ID:63767680 hemA\| glutamyl tRNA reductase [Denitratisoma oestradiolicum Fahrbach xx] | -0.6361 | 0.007973 |
| DENOEST_v1_0447 | ID:63767671 bioB\| biotin synthase [Denitratisoma oestradiolicum Fahrbach xx] | -0.74069 | 0.000108 |
| DENOEST_v1_0445 | ID:63767669 conserved protein of unknown function [Denitratisoma oestradiolicum Fahrbach xx] | 0.288362 | 0.027754 |
| DENOEST_v1_0429 | ID:63767653 epsE\| Type II secretion system protein E [Denitratisoma oestradiolicum Fahrbach xx] | -0.20579 | 0.02987 |
| DENOEST_v1_0425 | ID:63767649 secB\| protein export chaperone [Denitratisoma oestradiolicum Fahrbach xx] | -0.76717 | 0.018786 |
| DENOEST_v1_0423 | ID:63767647 Sulfurtransferase [Denitratisoma oestradiolicum Fahrbach xx] | -1.00958 | 0.001904 |
| DENOEST_v1_0422 | ID:63767646 Transcriptional regulator [Denitratisoma oestradiolicum Fahrbach xx] | -0.71941 | 0.009907 |
| DENOEST_v1_0421 | ID:63767645 gpmA\| phosphoglyceromutase 1 [Denitratisoma oestradiolicum Fahrbach xx] | -0.62013 | 0.000952 |
| DENOEST_v1_0419 | ID:63767643 ctpA\| Carboxy-terminal-processing protease [Denitratisoma oestradiolicum Fahrbach xx] | -0.32476 | 0.010824 |
| DENOEST_v1_0397 | ID:63767621 ppc\| Phosphoenolpyruvate carboxylase [Denitratisoma oestradiolicum Fahrbach xx] | 1.803443 | 0.045404 |
| DENOEST_v1_0394 | ID:63767618 Sulfatase [Denitratisoma oestradiolicum Fahrbach xx] | 1.540975 | 0.000597 |
| DENOEST_v1_0390 | ID:63767614 ubiD\| 3-octaprenyl-4-hydroxybenzoate decarboxylase [Denitratisoma oestradiolicum Fahrbach xx] | -0.30341 | 0.004631 |
| DENOEST_v1_0385 | ID:63767609 Ferritin [Denitratisoma oestradiolicum Fahrbach xx] | 0.83184 | 0.017706 |
| DENOEST_v1_0379 | ID:63767603 NAD(P)-dependent dehydrogenase (Short-subunit alcohol dehydrogenase family) [Denitratisoma oestradiolicum Fahrbach xx] | 2.044632 | 0.00392 |
| DENOEST_v1_0377 | ID:63767601 conserved exported protein of unknown function [Denitratisoma oestradiolicum Fahrbach xx] | -1.08336 | 0.008316 |
| DENOEST_v1_0369 | ID:63767593 exported protein of unknown function [Denitratisoma oestradiolicum Fahrbach xx] | 1.215999 | 0.014986 |
| DENOEST_v1_0368 | ID:63767592 Peptidase C1A papain [Denitratisoma oestradiolicum Fahrbach xx] | 1.138991 | 0.000108 |
| DENOEST_v1_0364 | ID:63767588 RutC family protein in vnfA 5'region [Denitratisoma oestradiolicum Fahrbach xx] | -0.67098 | 0.02107 |
| DENOEST_v1_0363 | ID:63767587 fliW\| Flagellar assembly factor FliW [Denitratisoma oestradiolicum Fahrbach xx] | -0.65052 | 0.015051 |
| DENOEST_v1_0362 | ID:63767586 conserved protein of unknown function [Denitratisoma oestradiolicum Fahrbach xx] | -1.03472 | 4.44E-05 |
| DENOEST_v1_0361 | ID:63767585 conserved protein of unknown function [Denitratisoma oestradiolicum Fahrbach xx] | -0.91265 | 0.01826 |
| DENOEST_v1_0351 | ID:63767575 tyrS\| Tyrosine--tRNA ligase [Denitratisoma oestradiolicum Fahrbach xx] | -0.4611 | 0.000464 |
| DENOEST_v1_0348 | ID:63767572 putative chaperone involved in Fe-S cluster assembly and activation (HesB-like) [Denitratisoma oestradiolicum Fahrbach xx] | -1.3396 | 0.000895 |
| DENOEST_v1_0345 | ID:63767569 argC\| N-acetyl-gamma-glutamyl-phosphate reductase [Denitratisoma oestradiolicum Fahrbach xx] | -0.49624 | 0.001828 |
| DENOEST_v1_0344 | ID:63767568 rpsI\| 30S ribosomal protein S9 [Denitratisoma oestradiolicum Fahrbach xx] | -1.36856 | 0.001486 |
| DENOEST_v1_0343 | ID:63767567 rplM\| 50S ribosomal protein L13 [Denitratisoma oestradiolicum Fahrbach xx] | -0.97047 | 0.001921 |
| DENOEST_v1_0339 | ID:63767563 rlmL\| Ribosomal RNA large subunit methyltransferase L [Denitratisoma oestradiolicum Fahrbach xx] | -0.34191 | 0.011077 |
| DENOEST_v1_0337 | ID:63767561 NAD(P)-dependent oxidoreductase [Denitratisoma oestradiolicum Fahrbach xx] | -0.66566 | 0.008631 |
| DENOEST_v1_0336 | ID:63767560 ascD\| CDP-6-deoxy-L-threo-D-glycero-4-hexulose-3-dehydrase reductase [Denitratisoma oestradiolicum Fahrbach xx] | -0.81366 | 0.007742 |
| DENOEST_v1_0333 | ID:63767557 Cyclic nucleotide-binding protein [Denitratisoma oestradiolicum Fahrbach xx] | 1.638027 | 0.00534 |
| DENOEST_v1_0332 | ID:63767556 conserved exported protein of unknown function [Denitratisoma oestradiolicum Fahrbach xx] | 0.632197 | 0.002632 |
| DENOEST_v1_0329 | ID:63767553 Iron complex outermembrane recepter protein [Denitratisoma oestradiolicum Fahrbach xx] | 3.890127 | 0.001081 |
| DENOEST_v1_0321 | ID:63767545 exported protein of unknown function [Denitratisoma oestradiolicum Fahrbach xx] | -1.28756 | 7.92E-06 |
| DENOEST_v1_0320 | ID:63767544 nirM\| Cytochrome c-551 [Denitratisoma oestradiolicum Fahrbach xx] | -1.0668 | 7.33E-05 |
| DENOEST_v1_0319 | ID:63767543 conserved protein of unknown function [Denitratisoma oestradiolicum Fahrbach xx] | -3.85851 | 0.005671 |
| DENOEST_v1_0316 | ID:63767540 cph2\| Phytochrome-like protein cph2 [Denitratisoma oestradiolicum Fahrbach xx] | -1.02391 | 0.002313 |
| DENOEST_v1_0314 | ID:63767538 ilvD\| dihydroxy-acid dehydratase [Denitratisoma oestradiolicum Fahrbach xx] | -0.50212 | 0.006297 |
| DENOEST_v1_0311 | ID:63767535 ybeY\| putative metal-dependent hydrolase [Denitratisoma oestradiolicum Fahrbach xx] | -0.58606 | 0.045843 |
| DENOEST_v1_0310 | ID:63767534 ybeZ\| putative enzyme with nucleoside triphosphate hydrolase domain [Denitratisoma oestradiolicum Fahrbach xx] | -0.40212 | 0.008269 |
| DENOEST_v1_0305 | ID:63767529 conserved protein of unknown function [Denitratisoma oestradiolicum Fahrbach xx] | -0.93249 | 0.01489 |
| DENOEST_v1_0303 | ID:63767527 aspS\| aspartyl-tRNA synthetase [Denitratisoma oestradiolicum Fahrbach xx] | -0.6777 | 0.000202 |
| DENOEST_v1_0297 | ID:63767521 ffh\| 4.5S-RNP protein, GTP binding export factor, part of signal recognition particle with 4.5 RNA [Denitratisoma oestradiolicum Fahrbach xx] | -0.75055 | 0.033281 |
| DENOEST_v1_0294 | ID:63767518 nudH\| nucleotide hydrolase [Denitratisoma oestradiolicum Fahrbach xx] | -0.87085 | 0.001613 |
| DENOEST_v1_0293 | ID:63767517 conserved exported protein of unknown function [Denitratisoma oestradiolicum Fahrbach xx] | -0.32738 | 0.007578 |
| DENOEST_v1_0290 | ID:63767514 coq\| 2-nonaprenyl-3-methyl-6-methoxy-1,4-benzoquinol hydroxylase [Denitratisoma oestradiolicum Fahrbach xx] | -0.42373 | 0.002974 |
| DENOEST_v1_0287 | ID:63767511 rpoH\| RNA polymerase, sigma 32 (sigma H) factor [Denitratisoma oestradiolicum Fahrbach xx] | -0.92409 | 0.045066 |
| DENOEST_v1_0283 | ID:63767507 Ribonuclease II [Denitratisoma oestradiolicum Fahrbach xx] | -0.56425 | 0.000393 |
| DENOEST_v1_0279 | ID:63767503 Multiple antibiotic resistance protein marR (modular protein) [Denitratisoma oestradiolicum Fahrbach xx] | -0.79332 | 0.006961 |
| DENOEST_v1_0278 | ID:63767502 yheS\| fused putative transporter subunits of ABC superfamily: ATP-binding components [Denitratisoma oestradiolicum Fahrbach xx] | -1.26056 | 0.001381 |
| DENOEST_v1_0276 | ID:63767500 ybiT\| putative transporter fused subunits of ABC superfamily: ATP-binding components [Denitratisoma oestradiolicum Fahrbach xx] | -1.15611 | 0.003738 |
| DENOEST_v1_0275 | ID:63767499 Eco57I restriction endonuclease [Denitratisoma oestradiolicum Fahrbach xx] | -16.46 | 0.000255 |
| DENOEST_v1_0274 | ID:63767498 TetR family transcriptional regulator [Denitratisoma oestradiolicum Fahrbach xx] | 0.499419 | 0.036852 |
| DENOEST_v1_0271 | ID:63767495 pcm\| Protein-L-isoaspartate O-methyltransferase [Denitratisoma oestradiolicum Fahrbach xx] | -0.51321 | 0.020891 |
| DENOEST_v1_0261 | ID:63767485 conserved protein of unknown function [Denitratisoma oestradiolicum Fahrbach xx] | -0.84023 | 0.007297 |
| DENOEST_v1_0260 | ID:63767484 apaH\| Bis(5'-nucleosyl)-tetraphosphatase, symmetrical [Denitratisoma oestradiolicum Fahrbach xx] | -0.53766 | 0.005465 |
| DENOEST_v1_0255 | ID:63767479 pilU\| twitching motility protein [Denitratisoma oestradiolicum Fahrbach xx] | 0.369832 | 0.044657 |
| DENOEST_v1_0251 | ID:63767475 proC\| Pyrroline-5-carboxylate reductase [Denitratisoma oestradiolicum Fahrbach xx] | -0.36013 | 0.026167 |
| DENOEST_v1_0248 | ID:63767472 Oxidoreductase [Denitratisoma oestradiolicum Fahrbach xx] | -0.27227 | 0.017191 |
| DENOEST_v1_0247 | ID:63767471 Oxidoreductase [Denitratisoma oestradiolicum Fahrbach xx] | -0.15614 | 0.041294 |
| DENOEST_v1_0241 | ID:63767465 glyQ\| glycyl-tRNA synthetase, alpha chain [Denitratisoma oestradiolicum Fahrbach xx] | -0.74851 | 0.004875 |
| DENOEST_v1_0239 | ID:63767463 glyS\| glycine tRNA synthetase, beta subunit [Denitratisoma oestradiolicum Fahrbach xx] | -0.64605 | 0.012556 |
| DENOEST_v1_0238 | ID:63767462 D-glycero-beta-D-manno-heptose-1,7-bisphosphate 7-phosphatase [Denitratisoma oestradiolicum Fahrbach xx] | -0.69453 | 0.00291 |
| DENOEST_v1_0236 | ID:63767460 conserved protein of unknown function [Denitratisoma oestradiolicum Fahrbach xx] | -16.3552 | 0.001872 |
| DENOEST_v1_0234 | ID:63767458 Uncharacterized copper-binding protein, cupredoxin-like subfamily [Denitratisoma oestradiolicum Fahrbach xx] | 0.674078 | 0.032253 |
| DENOEST_v1_0229 | ID:63767453 conserved protein of unknown function [Denitratisoma oestradiolicum Fahrbach xx] | 1.242996 | 0.034462 |
| DENOEST_v1_0224 | ID:63767448 Cytochrome c, class I [Denitratisoma oestradiolicum Fahrbach xx] | -1.19554 | 0.015868 |
| DENOEST_v1_0217 | ID:63767441 17 kDa surface antigen [Denitratisoma oestradiolicum Fahrbach xx] | 1.169857 | 0.001091 |
| DENOEST_v1_0216 | ID:63767440 Peptidase C14 caspase catalytic subunit p20 [Denitratisoma oestradiolicum Fahrbach xx] | 1.038265 | 8E-05 |
| DENOEST_v1_0215 | ID:63767439 OmpA/MotB domain-containing protein [Denitratisoma oestradiolicum Fahrbach xx] | 0.665856 | 0.000434 |
| DENOEST_v1_0214 | ID:63767438 conserved exported protein of unknown function [Denitratisoma oestradiolicum Fahrbach xx] | 0.969509 | 0.00061 |
| DENOEST_v1_0209 | ID:63767433 conserved protein of unknown function [Denitratisoma oestradiolicum Fahrbach xx] | 2.456409 | 0.030467 |
| DENOEST_v1_0208 | ID:63767432 conserved exported protein of unknown function [Denitratisoma oestradiolicum Fahrbach xx] | 5.486382 | 0.009739 |
| DENOEST_v1_0204 | ID:63767428 conserved protein of unknown function [Denitratisoma oestradiolicum Fahrbach xx] | 1.023543 | 0.00598 |
| DENOEST_v1_0203 | ID:63767427 Peptidoglycan-binding domain 1 protein [Denitratisoma oestradiolicum Fahrbach xx] | 0.366297 | 0.022145 |
| DENOEST_v1_0202 | ID:63767426 conserved exported protein of unknown function [Denitratisoma oestradiolicum Fahrbach xx] | 1.394428 | 0.000103 |
| DENOEST_v1_0198 | ID:63767422 conserved exported protein of unknown function [Denitratisoma oestradiolicum Fahrbach xx] | 0.745426 | 0.010759 |
| DENOEST_v1_0192 | ID:63767416 cca\| fused tRNA nucleotidyl transferase ; 2'3'-cyclic phosphodiesterase and 2'nucleotidase and phosphatase [Denitratisoma oestradiolicum Fahrbach xx] | 0.638037 | 0.007691 |
| DENOEST_v1_0188 | ID:63767412 conserved protein of unknown function [Denitratisoma oestradiolicum Fahrbach xx] | 0.770312 | 0.01729 |
| DENOEST_v1_0184 | ID:63767408 rbr\| Rubrerythrin [Denitratisoma oestradiolicum Fahrbach xx] | 0.627418 | 0.036261 |
| DENOEST_v1_0182 | ID:63767406 putative ShlB/FhaC/HecB family hemolysin secretion/activation protein [Denitratisoma oestradiolicum Fahrbach xx] | 0.783048 | 0.00582 |
| DENOEST_v1_0181 | ID:63767405 Filamentous hemagglutinin family outer membrane protein [Denitratisoma oestradiolicum Fahrbach xx] | 1.084131 | 0.032171 |
| DENOEST_v1_0180 | ID:63767404 conserved protein of unknown function [Denitratisoma oestradiolicum Fahrbach xx] | 1.3659 | 0.002718 |
| DENOEST_v1_0176 | ID:63767400 Phosphonate ABC transporter substrate-binding protein [Denitratisoma oestradiolicum Fahrbach xx] | -1.08111 | 0.019042 |
| DENOEST_v1_0173 | ID:63767397 nodI\| Nod factor export ATP-binding protein I [Denitratisoma oestradiolicum Fahrbach xx] | -0.76296 | 0.009065 |
| DENOEST_v1_0167 | ID:63767391 conserved protein of unknown function [Denitratisoma oestradiolicum Fahrbach xx] | -2.65886 | 0.001059 |
| DENOEST_v1_0166 | ID:63767390 yhbV\| protease [Denitratisoma oestradiolicum Fahrbach xx] | -0.33277 | 0.010182 |
| DENOEST_v1_0164 | ID:63767388 Putrescine-binding periplasmic protein [Denitratisoma oestradiolicum Fahrbach xx] | 0.704724 | 0.045382 |
| DENOEST_v1_0161 | ID:63767385 potG\| putrescine transporter subunit: ATP-binding component of ABC superfamily [Denitratisoma oestradiolicum Fahrbach xx] | 0.582024 | 0.034801 |
| DENOEST_v1_0155 | ID:63767379 3-alpha-(or 20-beta)-hydroxysteroid dehydrogenase [Denitratisoma oestradiolicum Fahrbach xx] | -0.29459 | 0.024574 |
| DENOEST_v1_0154 | ID:63767378 putative oxidoreductase [Denitratisoma oestradiolicum Fahrbach xx] | -0.63944 | 0.006122 |
| DENOEST_v1_0153 | ID:63767377 argS\| Arginine--tRNA ligase [Denitratisoma oestradiolicum Fahrbach xx] | -1.26407 | 0.000398 |
| DENOEST_v1_0149 | ID:63767373 putative Quercetin 2,3-dioxygenase [Denitratisoma oestradiolicum Fahrbach xx] | -0.78322 | 0.011397 |
| DENOEST_v1_0145 | ID:63767369 Disulfide bond formation protein DsbA [Denitratisoma oestradiolicum Fahrbach xx] | -0.57309 | 0.00171 |
| DENOEST_v1_0144 | ID:63767368 conserved protein of unknown function [Denitratisoma oestradiolicum Fahrbach xx] | 0.763009 | 0.006111 |
| DENOEST_v1_0142 | ID:63767366 conserved protein of unknown function [Denitratisoma oestradiolicum Fahrbach xx] | 1.825661 | 0.003167 |
| DENOEST_v1_0139 | ID:63767363 putative toxin-antitoxin system toxin component, PIN family [Denitratisoma oestradiolicum Fahrbach xx] | -3.1669 | 0.000177 |
| DENOEST_v1_0138 | ID:63767362 phaC\| Poly(3-hydroxyalkanoate) polymerase subunit PhaC [Denitratisoma oestradiolicum Fahrbach xx] | -1.63374 | 0.001228 |
| DENOEST_v1_0136 | ID:63767360 conserved exported protein of unknown function [Denitratisoma oestradiolicum Fahrbach xx] | -0.63551 | 0.010148 |
| DENOEST_v1_0134 | ID:63767358 Fe-S-cluster-containing hydrogenase subunit [Denitratisoma oestradiolicum Fahrbach xx] | -1.21924 | 0.000211 |
| DENOEST_v1_0123 | ID:63767347 conserved exported protein of unknown function [Denitratisoma oestradiolicum Fahrbach xx] | 1.54557 | 0.015658 |
| DENOEST_v1_0120 | ID:63767344 Lipid-transfer protein [Denitratisoma oestradiolicum Fahrbach xx] | -0.42645 | 0.016761 |
| DENOEST_v1_0119 | ID:63767343 Benzoylsuccinyl-CoA thiolase [Denitratisoma oestradiolicum Fahrbach xx] | -0.70464 | 0.003004 |
| DENOEST_v1_0118 | ID:63767342 ribE\| Riboflavin synthase [Denitratisoma oestradiolicum Fahrbach xx] | -0.64379 | 0.001337 |
| DENOEST_v1_0117 | ID:63767341 gshA\| Glutamate--cysteine ligase [Denitratisoma oestradiolicum Fahrbach xx] | -1.19721 | 0.003002 |
| DENOEST_v1_0110 | ID:63767334 etfB\| electron transfer flavoprotein beta-subunit [Denitratisoma oestradiolicum Fahrbach xx] | -0.37563 | 0.020835 |
| DENOEST_v1_0109 | ID:63767333 etfA\| electron transfer flavoprotein alpha-subunit [Denitratisoma oestradiolicum Fahrbach xx] | -0.42456 | 0.009854 |
| DENOEST_v1_0104 | ID:63767328 putative kinase Y4mE [Denitratisoma oestradiolicum Fahrbach xx] | 0.759331 | 0.011662 |
| DENOEST_v1_0099 | ID:63767323 transposase [Denitratisoma oestradiolicum Fahrbach xx] | -0.87728 | 0.036988 |
| DENOEST_v1_0098 | ID:63767322 Integrase [Denitratisoma oestradiolicum Fahrbach xx] | 1.721587 | 0.009826 |
