# Supplementary material for: Channeling C1 Metabolism toward S-Adenosylmethionine-Dependent Conversion of Estrogens to Androgens in Estrogen-Degrading Bacteria
Source: mBio. 2020 Aug 25;11(4):e01259-20. doi: 10.1128/mBio.01259-20 (PMC7448270; doi:10.1128/mBio.01259-20)
Supplement: TABLE S3 [file mBio.01259-20-st003.docx]

**Table S3.** List of differentially regulated genes in *D. oestradiolicum*. The log2 ratios for proteins abundances during growth with 17β-estradiol/nitrate and acetate/nitrate are shown. Similarities of differentially abundant gene products to those from other organisms are indicated.

| ***Denitratisoma oestradiolicum*** | | | ***Denitratisoma sp.* DHT3** | | | ***Steroidobacter denitrificans*** | | | ***Sterolibacterium denitrificans*** | | | **Proteobacterial steroid degraders (aerobic)** | | | | **Actinobacterial steroid degraders (aerobic)** | | | |
| --- | --- | --- | --- | --- | --- | --- | --- | --- | --- | --- | --- | --- | --- | --- | --- | --- | --- | --- | --- |
| Locus Tag | Annotation in *D. oestradiolicum* | Regulation (log2ratio) | E-value | Identitiy (%) | Accesion Nr. | E-value | Identitiy (%) | Accesion Nr. | E-value | Identitiy (%) | Accesion Nr. | Target species | E-value | Identitiy (%) | Accesion Nr. | Target species | E-value | Identitiy (%) | Accesion Nr. |
| **β-oxidation induced gene cluster (Ring C + D)** | | | | | | | | | | | | | | | | | | | |
| 1366 | CoA transferase subunit A | 2.6 | 0 | 92.1 | [WP_145843437.1](https://www.ncbi.nlm.nih.gov/protein/WP_145843437.1?report=genbank&log$=prottop&blast_rank=1&RID=WX4EG8E4015) |  |  |  | 7E-174 | 77.66 | [WP_067170630.1](https://www.ncbi.nlm.nih.gov/protein/WP_067170630.1?report=genbank&log$=prottop&blast_rank=2&RID=WX4EG8E4015) | *Pseudomonas* sp. Chol1 | 2E-161 | 70.79 | [WP_008568676.1](https://www.ncbi.nlm.nih.gov/protein/WP_008568676.1?report=genbank&log$=prottop&blast_rank=4&RID=WX4EG8E4015) | *Rhodococcus jostii* RHA1 | 1E-85 | 48.4 | [ABG96437.1](https://www.ncbi.nlm.nih.gov/protein/ABG96437.1?report=genbank&log$=prottop&blast_rank=7&RID=WX4EG8E4015) |
| 1367 | ketoacid CoA transferase | 2.6 | 0 | 95.13 | [WP_145841106.1](https://www.ncbi.nlm.nih.gov/protein/WP_145841106.1?report=genbank&log$=prottop&blast_rank=1&RID=WX4F8XHZ015) |  |  |  | 1-137 | 70.52 | [WP_067170633.1](https://www.ncbi.nlm.nih.gov/protein/WP_067170633.1?report=genbank&log$=prottop&blast_rank=3&RID=WX4F8XHZ015) | *Pseudomonas* sp. Chol1 | 7E-151 | 74.44 | [WP_008568677.1](https://www.ncbi.nlm.nih.gov/protein/WP_008568677.1?report=genbank&log$=prottop&blast_rank=2&RID=WX4F8XHZ015) | *Rhodococcus jostii* RHA1 | 2E-59 | 41.7 | [ABG96436.1](https://www.ncbi.nlm.nih.gov/protein/ABG96436.1?report=genbank&log$=prottop&blast_rank=6&RID=WX4F8XHZ015) |
| 1368 | enoyl-CoA hydratase | 2.2 | 1E-167 | 93.23 | [WP_145841104.1](https://www.ncbi.nlm.nih.gov/protein/WP_145841104.1?report=genbank&log$=prottop&blast_rank=1&RID=WX55HFP5015) |  |  |  | 8.00E-122 | 72.62 | [WP_067170635.1](https://www.ncbi.nlm.nih.gov/protein/WP_067170635.1?report=genbank&log$=prottop&blast_rank=4&RID=WX55HFP5015) | *C. testosteroni* ATCC 11996 | 1E-155 | 83.53 | [EHN64492.1](https://www.ncbi.nlm.nih.gov/protein/EHN64492.1?report=genbank&log$=prottop&blast_rank=2&RID=WX55HFP5015) | *Rhodococcus jostii* RHA1 | 7E-73 | 48.13 | [ABG96438.1](https://www.ncbi.nlm.nih.gov/protein/ABG96438.1?report=genbank&log$=prottop&blast_rank=6&RID=WX55HFP5015) |
| 1369 | acyl-CoA dehydrogenase | 2.3 | 0 | 97.16 | [WP_145841103.1](https://www.ncbi.nlm.nih.gov/protein/WP_145841103.1?report=genbank&log$=prottop&blast_rank=1&RID=WX5BWAD9015) |  |  |  | 0 | 74.55 | [WP_067169281.1](https://www.ncbi.nlm.nih.gov/protein/WP_067169281.1?report=genbank&log$=prottop&blast_rank=2&RID=WX5BWAD9015) | *C. testosteroni* ATCC 11996 | 0 | 70.54 | [EHN64491.1](https://www.ncbi.nlm.nih.gov/protein/EHN64491.1?report=genbank&log$=prottop&blast_rank=3&RID=WX5BWAD9015) | *Rhodococcus jostii* RHA1 | 5E-143 | 50.76 | [ABG96379.1](https://www.ncbi.nlm.nih.gov/protein/ABG96379.1?report=genbank&log$=prottop&blast_rank=6&RID=WX5BWAD9015) |
| 1370 | acyl-CoA dehydrogenase | 2.6 | 0 | 96.62 | [WP_145841102.1](https://www.ncbi.nlm.nih.gov/protein/WP_145841102.1?report=genbank&log$=prottop&blast_rank=1&RID=WX5HMWJH015) | 6E-121 | 54.44 | [WP_066917846.1](https://www.ncbi.nlm.nih.gov/protein/WP_066917846.1?report=genbank&log$=prottop&blast_rank=5&RID=WX5HMWJH015) | 2.00E-140 | 60.56 | [WP_067169284.1](https://www.ncbi.nlm.nih.gov/protein/WP_067169284.1?report=genbank&log$=prottop&blast_rank=3&RID=WX5HMWJH015) | *Pseudomonas* sp. Chol1 | 5E-149 | 61.02 | [WP_008568681.1](https://www.ncbi.nlm.nih.gov/protein/WP_008568681.1?report=genbank&log$=prottop&blast_rank=2&RID=WX5HMWJH015) | *Rhodococcus jostii* RHA1 | 8E-55 | 36.6 | [ABG96378.1](https://www.ncbi.nlm.nih.gov/protein/ABG96378.1?report=genbank&log$=prottop&blast_rank=6&RID=WX5HMWJH015) |
| 1371 | acetyl-CoA C-acetyltransferase | 2.5 | 0 | 96.35 | [WP_145841101.1](https://www.ncbi.nlm.nih.gov/protein/WP_145841101.1?report=genbank&log$=prottop&blast_rank=1&RID=WX5NX97B014) | 0 | 88.28 | [WP_066917847.1](https://www.ncbi.nlm.nih.gov/protein/WP_066917847.1?report=genbank&log$=prottop&blast_rank=4&RID=WX5NX97B014) | 0 | 90.89 | [WP_067169288.1](https://www.ncbi.nlm.nih.gov/protein/WP_067169288.1?report=genbank&log$=prottop&blast_rank=3&RID=WX5NX97B014) | *C. testosteroni* ATCC 11996 | 0 | 91.1 | [EHN64489.1](https://www.ncbi.nlm.nih.gov/protein/EHN64489.1?report=genbank&log$=prottop&blast_rank=2&RID=WX5NX97B014) | *Rhodococcus jostii* RHA1 | 0 | 65.27 | [ABG96385.1](https://www.ncbi.nlm.nih.gov/protein/ABG96385.1?report=genbank&log$=prottop&blast_rank=6&RID=WX5NX97B014) |
| 1372 | SDR family NAD(P)-dependent oxidoreductase | 1.5 | 0 | 96.25 | [WP_145841100.1](https://www.ncbi.nlm.nih.gov/protein/WP_145841100.1?report=genbank&log$=prottop&blast_rank=1&RID=WX61AD8T014) | 1E-138 | 66.21 | [WP_066917848.1](https://www.ncbi.nlm.nih.gov/protein/WP_066917848.1?report=genbank&log$=prottop&blast_rank=4&RID=WX61AD8T014) | 1.00E-144 | 67.58 | [WP_067170989.1](https://www.ncbi.nlm.nih.gov/protein/WP_067170989.1?report=genbank&log$=prottop&blast_rank=2&RID=WX61AD8T014) | *C. testosteroni* ATCC 11996 | 2E-139 | 64.16 | [EHN64486.1](https://www.ncbi.nlm.nih.gov/protein/EHN64486.1?report=genbank&log$=prottop&blast_rank=3&RID=WX61AD8T014) | *M. tuberculosis* H37Rv | 6E-104 | 53.27 | [NP_218065.1](https://www.ncbi.nlm.nih.gov/protein/NP_218065.1?report=genbank&log$=prottop&blast_rank=5&RID=WX61AD8T014) |
| 1373 | SDR family oxidoreductase | 1.6 | 0 | 96.93 | [WP_145841099.1](https://www.ncbi.nlm.nih.gov/protein/WP_145841099.1?report=genbank&log$=prottop&blast_rank=1&RID=WX65U0ZW014) | 8E-134 | 73.18 | [WP_066917851.1](https://www.ncbi.nlm.nih.gov/protein/WP_066917851.1?report=genbank&log$=prottop&blast_rank=4&RID=WX65U0ZW014) | 6.00E+153 | 77.39 | [WP_067170997.1](https://www.ncbi.nlm.nih.gov/protein/WP_067170997.1?report=genbank&log$=prottop&blast_rank=2&RID=WX65U0ZW014) | *Pseudomonas* sp. Chol1 | 1E-142 | 77.78 | [WP_008568684.1](https://www.ncbi.nlm.nih.gov/protein/WP_008568684.1?report=genbank&log$=prottop&blast_rank=3&RID=WX65U0ZW014) | *Rhodococcus jostii* RHA1 | 9E-118 | 63.74 | [ABG96383.1](https://www.ncbi.nlm.nih.gov/protein/ABG96383.1?report=genbank&log$=prottop&blast_rank=6&RID=WX65U0ZW014) |
| 1374 | MaoC family dehydratase | 2 | 4E-111 | 95.39 | [WP_145841098.1](https://www.ncbi.nlm.nih.gov/protein/WP_145841098.1?report=genbank&log$=prottop&blast_rank=1&RID=WX6H27SB015) | 2E-69 | 59.87 | [WP_066917852.1](https://www.ncbi.nlm.nih.gov/protein/WP_066917852.1?report=genbank&log$=prottop&blast_rank=4&RID=WX6H27SB015) | 4.00E-79 | 66.23 | [WP_067171000.1](https://www.ncbi.nlm.nih.gov/protein/WP_067171000.1?report=genbank&log$=prottop&blast_rank=2&RID=WX6H27SB015) | *C. testosteroni* ATCC 11996 | 2E-68 | 60.67 | [EHN64482.1](https://www.ncbi.nlm.nih.gov/protein/EHN64482.1?report=genbank&log$=prottop&blast_rank=6&RID=WX6H27SB015) | *M. tuberculosis* H37Rv | 1E-54 | 55.17 | [NP_214644.1](https://www.ncbi.nlm.nih.gov/protein/NP_214644.1?report=genbank&log$=prottop&blast_rank=7&RID=WX6H27SB015) |
| 1375 | 3-ketoacyl-CoA thiolase | 1.7 | 0 | 98.25 | [WP_145841097.1](https://www.ncbi.nlm.nih.gov/protein/WP_145841097.1?report=genbank&log$=prottop&blast_rank=1&RID=WX6YS08B01N) | 4E-175 | 60.9 | [WP_066917917.1](https://www.ncbi.nlm.nih.gov/protein/WP_066917917.1?report=genbank&log$=prottop&blast_rank=5&RID=WX6YS08B01N) | 0 | 81.25 | [SMB22261.1](https://www.ncbi.nlm.nih.gov/protein/SMB22261.1?report=genbank&log$=prottop&blast_rank=2&RID=WX6YS08B01N) | *Pseudomonas* sp. Chol1 | 0 | 71.93 | [WP_008568686.1](https://www.ncbi.nlm.nih.gov/protein/WP_008568686.1?report=genbank&log$=prottop&blast_rank=4&RID=WX6YS08B01N) | *Rhodococcus jostii* RHA1 | 2E-80 | 36.71 | [ABG94825.1](https://www.ncbi.nlm.nih.gov/protein/ABG94825.1?report=genbank&log$=prottop&blast_rank=12&RID=WX6YS08B01N) |
| 1376 | steroid delta-isomerase | 2.5 | 8E-81 | 89.6 | [WP_145843436.1](https://www.ncbi.nlm.nih.gov/protein/WP_145843436.1?report=genbank&log$=prottop&blast_rank=1&RID=WX74MSD6015) | - | - | - | - | - | - | - | - | - | - | - | - | - | - |
| 1377 | cholest-4-en-3-one-Δ1-isomerase | 1.7 | 0 | 89.41 | [WP_145841096.1](https://www.ncbi.nlm.nih.gov/protein/WP_145841096.1?report=genbank&log$=prottop&blast_rank=1&RID=WX7AM98S01N) | 0 | 68.06 | [WP_066922605.1](https://www.ncbi.nlm.nih.gov/protein/WP_066922605.1?report=genbank&log$=prottop&blast_rank=2&RID=WX7AM98S01N) | 0 | 52.67 | [ABV59992.1](https://www.ncbi.nlm.nih.gov/protein/ABV59992.1?report=genbank&log$=prottop&blast_rank=3&RID=WX7AM98S01N) | *C. testosteroni* ATCC 11996 | 2E-175 | 46 | [EHN64413.1](https://www.ncbi.nlm.nih.gov/protein/EHN64413.1?report=genbank&log$=prottop&blast_rank=4&RID=WX7AM98S01N) | *Rhodococcus jostii* RHA1 | 2E-112 | 37.68 | [ABG96318.1](https://www.ncbi.nlm.nih.gov/protein/ABG96318.1?report=genbank&log$=prottop&blast_rank=8&RID=WX7AM98S01N) |
| 1378 | long-chain-fatty-acid-CoA ligase | 1.2 | 0 | 88.68 | [WP_145841095.1](https://www.ncbi.nlm.nih.gov/protein/WP_145841095.1?report=genbank&log$=prottop&blast_rank=1&RID=WX7EHCUR014) | 0 | 55.1 | [WP_083536640.1](https://www.ncbi.nlm.nih.gov/protein/WP_083536640.1?report=genbank&log$=prottop&blast_rank=5&RID=WX7EHCUR014) | 0 | 63.95 | [WP_067171006.1](https://www.ncbi.nlm.nih.gov/protein/WP_067171006.1?report=genbank&log$=prottop&blast_rank=2&RID=WX7EHCUR014) | *C. testosteroni* ATCC 11996 | 0 | 60.16 | [EHN64410.1](https://www.ncbi.nlm.nih.gov/protein/EHN64410.1?report=genbank&log$=prottop&blast_rank=3&RID=WX7EHCUR014) | *Rhodococcus jostii* RHA1 | 4E-161 | 47.33 | [Q0S7V5.1](https://www.ncbi.nlm.nih.gov/protein/Q0S7V5.1?report=genbank&log$=prottop&blast_rank=8&RID=WX7EHCUR014) |
| 1379 | hypothetical protein | 1.9 | - | - | - | 6E-61 | 45.33 | [WP_066918050.1](https://www.ncbi.nlm.nih.gov/protein/WP_066918050.1?report=genbank&log$=prottop&blast_rank=1&RID=WX7KKX1U015) | - | - | - | - | - | - | - | - | - | - | - |
| **β-oxidation induced gene cluster (Ring A + B)** | | | | | | | | | | | | | | | | | | | |
| 2958 | enoyl-CoA hydratase | 1.7 | 0 | 95.29 | [WP_145841759.1](https://www.ncbi.nlm.nih.gov/protein/WP_145841759.1?report=genbank&log$=prottop&blast_rank=1&RID=WX7ZF2H701N) | - | - | - | 1.00E-163 | 80.07 | [SMB22664.1](https://www.ncbi.nlm.nih.gov/protein/SMB22664.1?report=genbank&log$=prottop&blast_rank=2&RID=WX7ZF2H701N) | - | - | - | - | - | - | - | - |
| 2959 | F420-dependent oxidoreductase | - | 0 | 98.04 | [WP_145841760.1](https://www.ncbi.nlm.nih.gov/protein/WP_145841760.1?report=genbank&log$=prottop&blast_rank=1&RID=WX876B8B015) | 2E-97 | 43.46 | [AMN46162.1](https://www.ncbi.nlm.nih.gov/protein/AMN46162.1?report=genbank&log$=prottop&blast_rank=3&RID=WX876B8B015) | 0 | 79.41 | [SMB22670.1](https://www.ncbi.nlm.nih.gov/protein/SMB22670.1?report=genbank&log$=prottop&blast_rank=2&RID=WX876B8B015) | - | - | - | - | - | - | - | - |
| 2960 | CoA transferase | 2.2 | 0 | 92.86 | [WP_145841761.1](https://www.ncbi.nlm.nih.gov/protein/WP_145841761.1?report=genbank&log$=prottop&blast_rank=1&RID=WX87SRP1015) | 0 | 73.4 | [WP_066917861.1](https://www.ncbi.nlm.nih.gov/protein/WP_066917861.1?report=genbank&log$=prottop&blast_rank=3&RID=WX87SRP1015) | 0 | 78.57 | [SMB22676.1](https://www.ncbi.nlm.nih.gov/protein/SMB22676.1?report=genbank&log$=prottop&blast_rank=2&RID=WX87SRP1015) | *Pseudomonas* sp. Chol1 | 3E-55 | 31.19 | [WP_008570268.1](https://www.ncbi.nlm.nih.gov/protein/WP_008570268.1?report=genbank&log$=prottop&blast_rank=9&RID=WX87SRP1015) | - | - | - | - |
| 2961 | CoA transferase | 1.8 | 0 | 92.42 | [WP_145841762.1](https://www.ncbi.nlm.nih.gov/protein/WP_145841762.1?report=genbank&log$=prottop&blast_rank=1&RID=WX8C3Y4M01N) | 0 | 76.96 | [WP_066917860.1](https://www.ncbi.nlm.nih.gov/protein/WP_066917860.1?report=genbank&log$=prottop&blast_rank=3&RID=WX8C3Y4M01N) | 0 | 87.99 | [SMB22681.1](https://www.ncbi.nlm.nih.gov/protein/SMB22681.1?report=genbank&log$=prottop&blast_rank=2&RID=WX8C3Y4M01N) | *Pseudomonas* sp. Chol1 | 1E-57 | 31.94 | [WP_008569598.1](https://www.ncbi.nlm.nih.gov/protein/WP_008569598.1?report=genbank&log$=prottop&blast_rank=10&RID=WX8C3Y4M01N) | *Rhodococcus jostii* RHA1 | 6E-55 | 33.16 | [ABG92598.1](https://www.ncbi.nlm.nih.gov/protein/ABG92598.1?report=genbank&log$=prottop&blast_rank=14&RID=WX8C3Y4M01N) |
| 2962 | acyl-CoA dehydrogenase | 3.1 | 0 | 86.05 | [WP_145841763.1](https://www.ncbi.nlm.nih.gov/protein/WP_145841763.1?report=genbank&log$=prottop&blast_rank=1&RID=WX8HE676015) | 0 | 69.43 | [WP_066918894.1](https://www.ncbi.nlm.nih.gov/protein/WP_066918894.1?report=genbank&log$=prottop&blast_rank=3&RID=WX8HE676015) | 0 | 70.54 | [SMB22683.1](https://www.ncbi.nlm.nih.gov/protein/SMB22683.1?report=genbank&log$=prottop&blast_rank=2&RID=WX8HE676015) | - | - | - | - | - | - | - | - |
| 2963 | MaoC domain protein dehydratase | 3.5 | 5E-101 | 83.85 | [WP_145841764.1](https://www.ncbi.nlm.nih.gov/protein/WP_145841764.1?report=genbank&log$=prottop&blast_rank=1&RID=WX8PNC4Y015) | 2E-83 | 73.33 | [WP_066918892.1](https://www.ncbi.nlm.nih.gov/protein/WP_066918892.1?report=genbank&log$=prottop&blast_rank=3&RID=WX8PNC4Y015) | 1.00E-90 | 74.53 | [SMB22684.1](https://www.ncbi.nlm.nih.gov/protein/SMB22684.1?report=genbank&log$=prottop&blast_rank=2&RID=WX8PNC4Y015) | - | - | - | - | - | - | - | - |
| 2964 | Maoc family dehydratase | 3.6 | 3E-117 | 88.44 | [WP_145841765.1](https://www.ncbi.nlm.nih.gov/protein/WP_145841765.1?report=genbank&log$=prottop&blast_rank=1&RID=WX8UWRGZ015) | 8E-65 | 53.85 | [WP_083536427.1](https://www.ncbi.nlm.nih.gov/protein/WP_083536427.1?report=genbank&log$=prottop&blast_rank=3&RID=WX8UWRGZ015) | 2.00E-93 | 74.4 | [SMB22687.1](https://www.ncbi.nlm.nih.gov/protein/SMB22687.1?report=genbank&log$=prottop&blast_rank=2&RID=WX8UWRGZ015) | - | - | - | - | - | - | - | - |
| 2965 | CoA transferase | 4 | 0 | 85.23 | [WP_145841766.1](https://www.ncbi.nlm.nih.gov/protein/WP_145841766.1?report=genbank&log$=prottop&blast_rank=1&RID=WX90D398015) | 0 | 73.32 | [WP_066918890.1](https://www.ncbi.nlm.nih.gov/protein/WP_066918890.1?report=genbank&log$=prottop&blast_rank=3&RID=WX90D398015) | 0 | 79.53 | [SMB22688.1](https://www.ncbi.nlm.nih.gov/protein/SMB22688.1?report=genbank&log$=prottop&blast_rank=2&RID=WX90D398015) | *C. testosteroni* ATCC 11996 | 9E-58 | 33.33 | [EHN64477.1](https://www.ncbi.nlm.nih.gov/protein/EHN64477.1?report=genbank&log$=prottop&blast_rank=16&RID=WX90D398015) | *Rhodococcus jostii* RHA1 | 2E-69 | 36.34 | [ABG99197.1](https://www.ncbi.nlm.nih.gov/protein/ABG99197.1?report=genbank&log$=prottop&blast_rank=7&RID=WX90D398015) |
| 2966 | acyl-CoA dehydrogenase | 3.5 | 0 | 92.11 | [WP_145841767.1](https://www.ncbi.nlm.nih.gov/protein/WP_145841767.1?report=genbank&log$=prottop&blast_rank=1&RID=WX9TMG3Y015) | 0 | 83.21 | [WP_066918888.1](https://www.ncbi.nlm.nih.gov/protein/WP_066918888.1?report=genbank&log$=prottop&blast_rank=2&RID=WX9TMG3Y015) | 0 | 82.44 | [SMB22689.1](https://www.ncbi.nlm.nih.gov/protein/SMB22689.1?report=genbank&log$=prottop&blast_rank=3&RID=WX9TMG3Y015) | *Pseudomonas* sp. Chol1 | 7E-64 | 35.21 | [WP_008568644.1](https://www.ncbi.nlm.nih.gov/protein/WP_008568644.1?report=genbank&log$=prottop&blast_rank=16&RID=WX9TMG3Y015) | *Rhodococcus jostii* RHA1 | 5E-66 | 29.85 | [ABG96479.1](https://www.ncbi.nlm.nih.gov/protein/ABG96479.1?report=genbank&log$=prottop&blast_rank=11&RID=WX9TMG3Y015) |
| 2967 | CoA transferase | 2.5 | 0 | 95.12 | [WP_145841768.1](https://www.ncbi.nlm.nih.gov/protein/WP_145841768.1?report=genbank&log$=prottop&blast_rank=1&RID=WX9Z41BR015) | 0 | 79.41 | [WP_066917867.1](https://www.ncbi.nlm.nih.gov/protein/WP_066917867.1?report=genbank&log$=prottop&blast_rank=4&RID=WX9Z41BR015) | 0 | 85.78 | [SMB21136.1](https://www.ncbi.nlm.nih.gov/protein/SMB21136.1?report=genbank&log$=prottop&blast_rank=2&RID=WX9Z41BR015) | - | - | - | - | *Rhodococcus jostii* RHA1 | 2E-62 | 35.12 | [ABG92253.1](https://www.ncbi.nlm.nih.gov/protein/ABG92253.1?report=genbank&log$=prottop&blast_rank=5&RID=WX9Z41BR015) |
| 2968 | putative amidohydrolase family protein | 1.3 | 0 | 96.6 | [WP_145841769.1](https://www.ncbi.nlm.nih.gov/protein/WP_145841769.1?report=genbank&log$=prottop&blast_rank=1&RID=WXA408Y8014) | 3E-67 | 36.2 | [WP_066918687.1](https://www.ncbi.nlm.nih.gov/protein/WP_066918687.1?report=genbank&log$=prottop&blast_rank=4&RID=WXA408Y8014) | 0 | 76.66 | [SMB22433.1](https://www.ncbi.nlm.nih.gov/protein/SMB22433.1?report=genbank&log$=prottop&blast_rank=2&RID=WXA408Y8014) | - | - | - | - | - | - | - | - |
| 2969 | NADPH-dependent F420 reductase | - | 5E-135 | 91.19 | [WP_145841770.1](https://www.ncbi.nlm.nih.gov/protein/WP_145841770.1?report=genbank&log$=prottop&blast_rank=1&RID=WXA8W7HY015) | 2E-60 | 51.12 | [WP_066920948.1](https://www.ncbi.nlm.nih.gov/protein/WP_066920948.1?report=genbank&log$=prottop&blast_rank=4&RID=WXA8W7HY015) | 2.00E-129 | 90.18 | [SMB22485.1](https://www.ncbi.nlm.nih.gov/protein/SMB22485.1?report=genbank&log$=prottop&blast_rank=2&RID=WXA8W7HY015) | - | - | - | - | - | - | - | - |
| 2970 | enoyl-CoA hydratase | - | 0 | 97.3 | [WP_145841771.1](https://www.ncbi.nlm.nih.gov/protein/WP_145841771.1?report=genbank&log$=prottop&blast_rank=1&RID=WXAC7DJA015) | - | - | - | 3.00E-172 | 86.49 | [SMB22484.1](https://www.ncbi.nlm.nih.gov/protein/SMB22484.1?report=genbank&log$=prottop&blast_rank=2&RID=WXAC7DJA015) | - | - | - | - | *M. tuberculosis* H37Rv | 3E-63 | 43.4 | [NP_218033.1](https://www.ncbi.nlm.nih.gov/protein/NP_218033.1?report=genbank&log$=prottop&blast_rank=4&RID=WXAC7DJA015) |
| 2971 | Zn-ribbon domain-containing OB-fold protein | 1.6 | 1E-95 | 92.7 | [WP_145841772.1](https://www.ncbi.nlm.nih.gov/protein/WP_145841772.1?report=genbank&log$=prottop&blast_rank=1&RID=WXE210S5015) | - | - | - | 4.00E-76 | 72.26 | [SMB22483.1](https://www.ncbi.nlm.nih.gov/protein/SMB22483.1?report=genbank&log$=prottop&blast_rank=2&RID=WXE210S5015) | - | - | - | - | - | - | - | - |
| 2972 | lipid-transfer protein | 1.5 | 0 | 93.28 | [WP_145841773.1](https://www.ncbi.nlm.nih.gov/protein/WP_145841773.1?report=genbank&log$=prottop&blast_rank=1&RID=WXE72KFF015) | 1E-94 | 46.23 | [WP_066918699.1](https://www.ncbi.nlm.nih.gov/protein/WP_066918699.1?report=genbank&log$=prottop&blast_rank=4&RID=WXE72KFF015) | 0 | 83.2 | [SMB22480.1](https://www.ncbi.nlm.nih.gov/protein/SMB22480.1?report=genbank&log$=prottop&blast_rank=2&RID=WXE72KFF015) | *Pseudomonas* sp. Chol1 | 7E-80 | 40.16 | [WP_008568651.1](https://www.ncbi.nlm.nih.gov/protein/WP_008568651.1?report=genbank&log$=prottop&blast_rank=12&RID=WXE72KFF015) | *Rhodococcus jostii* RHA1 | 9E-87 | 41.21 | [ABG96274.1](https://www.ncbi.nlm.nih.gov/protein/ABG96274.1?report=genbank&log$=prottop&blast_rank=5&RID=WXE72KFF015) |
| 2973 | 1,4-dihydroxy-6-napththoyl-CoA synthase | 1.7 | 0 | 95.99 | [WP_145841774.1](https://www.ncbi.nlm.nih.gov/protein/WP_145841774.1?report=genbank&log$=prottop&blast_rank=1&RID=WXEBA1CP015) | 0 | 83.11 | [WP_066917865.1](https://www.ncbi.nlm.nih.gov/protein/WP_066917865.1?report=genbank&log$=prottop&blast_rank=3&RID=WXEBA1CP015) | 0 | 86.2 | [SMB22477.1](https://www.ncbi.nlm.nih.gov/protein/SMB22477.1?report=genbank&log$=prottop&blast_rank=2&RID=WXEBA1CP015) | - | - | - | - | *Rhodococcus jostii* RHA1 | 3E-59 | 40 | [ABH00828.1](https://www.ncbi.nlm.nih.gov/protein/ABH00828.1?report=genbank&log$=prottop&blast_rank=5&RID=WXEBA1CP015) |
| 2974 | CoA transferase | 1.9 | 0 | 94.04 | [WP_145840652.1](https://www.ncbi.nlm.nih.gov/protein/WP_145840652.1?report=genbank&log$=prottop&blast_rank=1&RID=WXEHW9XY014) | 0 | 78 | [WP_066917864.1](https://www.ncbi.nlm.nih.gov/protein/WP_066917864.1?report=genbank&log$=prottop&blast_rank=3&RID=WXEHW9XY014) | 0 | 81.75 | [SMB22476.1](https://www.ncbi.nlm.nih.gov/protein/SMB22476.1?report=genbank&log$=prottop&blast_rank=2&RID=WXEHW9XY014) | *Pseudomonas* sp. Chol1 | 8E-61 | 32.27 | [WP_008570268.1](https://www.ncbi.nlm.nih.gov/protein/WP_008570268.1?report=genbank&log$=prottop&blast_rank=4&RID=WXEHW9XY014) | *Rhodococcus jostii* RHA1 | 5E-57 | 31.02 | [ABG99199.1](https://www.ncbi.nlm.nih.gov/protein/ABG99199.1?report=genbank&log$=prottop&blast_rank=10&RID=WXEHW9XY014) |
| 2975 | CoA transferase | 1.9 | 0 | 87.66 | [WP_145840653.1](https://www.ncbi.nlm.nih.gov/protein/WP_145840653.1?report=genbank&log$=prottop&blast_rank=1&RID=WXESEU32015) | 0 | 71.32 | [WP_066917863.1](https://www.ncbi.nlm.nih.gov/protein/WP_066917863.1?report=genbank&log$=prottop&blast_rank=3&RID=WXESEU32015) | 0 | 80.35 | [SMB22473.1](https://www.ncbi.nlm.nih.gov/protein/SMB22473.1?report=genbank&log$=prottop&blast_rank=2&RID=WXESEU32015) | - | - | - | - | - | - | - | - |
| **Methyl transferase / SAM recycling / cobalamin utilization induced gene cluster** | | | | | | | | | | | | | | | | | | | |
| 1153 | thymidylate synthase | 18.4 | - | - | - | - | - | - | - | - | - | - | - | - | - | - | - | - | - |
| 1154 | FAD-binding oxidoreductase | - | - | - | - | - | - | - | - | - | - | - | - | - | - | - | - | - | - |
| 1155 | phosphoribosylformylglycinamidine cyclo-ligase (PurM) | 23.2 | - | - | - | - | - | - | - | - | - | - | - | - | - | - | - | - | - |
| 1156 | cobalamin-binding protein | 4.1 | - | - | - | - | - | - | - | - | - | - | - | - | - | - | - | - | - |
| 1157 | - | - | - | - | - | - | - | - | - | - | - | - | - | - | - | - | - | - | - |
| 1158 | domain containing protein | - | - | - | - | - | - | - | - | - | - | - | - | - | - | - | - | - | - |
| **1159** | **putative vitamin B12 transporter protein** | **22.9** | **0** | **98.63** | [**WP_145842106.1**](https://www.ncbi.nlm.nih.gov/protein/WP_145842106.1?report=genbank&log$=prottop&blast_rank=1&RID=WXFTRKXN015) | **3E-71** | **43.87** | [**AMN45799.1**](https://www.ncbi.nlm.nih.gov/protein/AMN45799.1?report=genbank&log$=prottop&blast_rank=5&RID=WXFTRKXN015) | **1.00E-108** | **54.48** | [**SMB21070.1**](https://www.ncbi.nlm.nih.gov/protein/SMB21070.1?report=genbank&log$=prottop&blast_rank=3&RID=WXFTRKXN015) | **-** | **-** | **-** | **-** | **-** | **-** | **-** | **-** |
| **1160** | **threonine-phosphate decarboxylase** | **24.5** | **0** | **95.44** | [**WP_145842107.1**](https://www.ncbi.nlm.nih.gov/protein/WP_145842107.1?report=genbank&log$=prottop&blast_rank=1&RID=WXFV02HX015) | **3E-63** | **38.41** | [**AMN48414.1**](https://www.ncbi.nlm.nih.gov/protein/AMN48414.1?report=genbank&log$=prottop&blast_rank=5&RID=WXFV02HX015) | **6.00E-88** | **46.73** | [**SMB21063.1**](https://www.ncbi.nlm.nih.gov/protein/SMB21063.1?report=genbank&log$=prottop&blast_rank=2&RID=WXFV02HX015) | **-** | **-** | **-** | **-** | **-** | **-** | **-** | **-** |
| **1161** | **nicotinate-nucleotide-dimethylbenzimidazole phosphoribosyltransferase** | **5.6** | **0** | **98.58** | [**WP_145842108.1**](https://www.ncbi.nlm.nih.gov/protein/WP_145842108.1?report=genbank&log$=prottop&blast_rank=1&RID=WXFZX8FS015) | **1E-62** | **39.69** | [**WP_066922453.1**](https://www.ncbi.nlm.nih.gov/protein/WP_066922453.1?report=genbank&log$=prottop&blast_rank=6&RID=WXFZX8FS015) | **3.00E-72** | **43.03** | [**SMB21065.1**](https://www.ncbi.nlm.nih.gov/protein/SMB21065.1?report=genbank&log$=prottop&blast_rank=5&RID=WXFZX8FS015) | ***C. testosteroni* ATCC 11996** | **2E-103** | **50.15** | [**EHN64098.1**](https://www.ncbi.nlm.nih.gov/protein/EHN64098.1?report=genbank&log$=prottop&blast_rank=3&RID=WXFZX8FS015) | ***Rhodococcus jostii* RHA1** | **1E-57** | **40.48** | [**ABG92972.1**](https://www.ncbi.nlm.nih.gov/protein/ABG92972.1?report=genbank&log$=prottop&blast_rank=7&RID=WXFZX8FS015) |
| 1162 | cobalamin biosynthesis protein | - | 0 | 97.43 | [WP_145842109.1](https://www.ncbi.nlm.nih.gov/protein/WP_145842109.1?report=genbank&log$=prottop&blast_rank=1&RID=WXG2TND9015) | - | - | - | 2.00E-90 | 53.67 | [SMB21062.1](https://www.ncbi.nlm.nih.gov/protein/SMB21062.1?report=genbank&log$=prottop&blast_rank=3&RID=WXG2TND9015) | *Pseudomonas* sp. Chol1 | 1E-105 | 59.72 | [WP_008568179.1](https://www.ncbi.nlm.nih.gov/protein/WP_008568179.1?report=genbank&log$=prottop&blast_rank=2&RID=WXG2TND9015) | *Rhodococcus jostii* RHA1 | 2E-54 | 43.01 | [ABG93015.1](https://www.ncbi.nlm.nih.gov/protein/ABG93015.1?report=genbank&log$=prottop&blast_rank=5&RID=WXG2TND9015) |
| 1163 | putative glycosyltransferase | 7.7 | 2E-160 | 91.53 | [WP_145842110.1](https://www.ncbi.nlm.nih.gov/protein/WP_145842110.1?report=genbank&log$=prottop&blast_rank=1&RID=WXGB7H78014) | 7E-55 | 45.58 | [WP_066921709.1](https://www.ncbi.nlm.nih.gov/protein/WP_066921709.1?report=genbank&log$=prottop&blast_rank=2&RID=WXGB7H78014) | - | - | - | - | - | - | - | - | - | - | - |
| **1164** | **domain-containing protein** | **25.1** | **0** | **98.35** | [**WP_145842111.1**](https://www.ncbi.nlm.nih.gov/protein/WP_145842111.1?report=genbank&log$=prottop&blast_rank=1&RID=WXGGCYC5015) | **0** | **57.37** | [**WP_066921707.1**](https://www.ncbi.nlm.nih.gov/protein/WP_066921707.1?report=genbank&log$=prottop&blast_rank=2&RID=WXGGCYC5015) | **-** | **-** | **-** | **-** | **-** | **-** | **-** | **-** | **-** | **-** | **-** |
| **1165** | **putative adenosine kinase** | **26.3** | **0** | **86.5** | [**WP_145842112.1**](https://www.ncbi.nlm.nih.gov/protein/WP_145842112.1?report=genbank&log$=prottop&blast_rank=1&RID=WXGK24JF014) | **2E-145** | **64.4** | [**WP_066918244.1**](https://www.ncbi.nlm.nih.gov/protein/WP_066918244.1?report=genbank&log$=prottop&blast_rank=2&RID=WXGK24JF014) | **1.00E-130** | **57.84** | [**WP_067169137.1**](https://www.ncbi.nlm.nih.gov/protein/WP_067169137.1?report=genbank&log$=prottop&blast_rank=3&RID=WXGK24JF014) | ***C. testosteroni* ATCC 11996** | **2E-103** | **48.99** | [**EHN63101.1**](https://www.ncbi.nlm.nih.gov/protein/EHN63101.1?report=genbank&log$=prottop&blast_rank=5&RID=WXGK24JF014) | ***M. tuberculosis* H37Rv** | **8E-62** | **34.41** | [**NP_216718.1**](https://www.ncbi.nlm.nih.gov/protein/NP_216718.1?report=genbank&log$=prottop&blast_rank=6&RID=WXGK24JF014) |
| 1166 | tetR family transcriptional regulator | 3.5 | - | - | - | - | - | - | - | - | - | - | - | - | - | - | - | - | - |
| **1167** | **putative oxidoreductase** | **28** | **6E-144** | **62.59** | [**WP_145842114.1**](https://www.ncbi.nlm.nih.gov/protein/WP_145842114.1?report=genbank&log$=prottop&blast_rank=2&RID=WXH08M6M015) | **8E-146** | **63.48** | [**WP_066921700.1**](https://www.ncbi.nlm.nih.gov/protein/WP_066921700.1?report=genbank&log$=prottop&blast_rank=1&RID=WXH08M6M015) | **2.00E-54** | **33.87** | [**SMB22467.1**](https://www.ncbi.nlm.nih.gov/protein/SMB22467.1?report=genbank&log$=prottop&blast_rank=8&RID=WXH08M6M015) | **-** | **-** | **-** | **-** | ***Rhodococcus jostii* RHA1** | **4E-60** | **38.89** | [**ABG96468.1**](https://www.ncbi.nlm.nih.gov/protein/ABG96468.1?report=genbank&log$=prottop&blast_rank=5&RID=WXH08M6M015) |
| **1168** | **hypothetical protein** | **26.2** | **3E-57** | **77.45** | [**WP_145842115.1**](https://www.ncbi.nlm.nih.gov/protein/WP_145842115.1?report=genbank&log$=prottop&blast_rank=1&RID=WXH1ZTRW015) | **6E-52** | **72.55** | [**WP_066921698.1**](https://www.ncbi.nlm.nih.gov/protein/WP_066921698.1?report=genbank&log$=prottop&blast_rank=2&RID=WXH1ZTRW015) | **-** | **-** | **-** | **-** | **-** | **-** | **-** | **-** | **-** | **-** | **-** |
| **1169** | **cobalamin -binding protein** | **28.2** | **1E-150** | **88.98** | [**WP_145842116.1**](https://www.ncbi.nlm.nih.gov/protein/WP_145842116.1?report=genbank&log$=prottop&blast_rank=1&RID=WXH8E9F7015) | **5E-128** | **74.89** | [**WP_066921697.1**](https://www.ncbi.nlm.nih.gov/protein/WP_066921697.1?report=genbank&log$=prottop&blast_rank=2&RID=WXH8E9F7015) | **-** | **-** | **-** | **-** | **-** | **-** | **-** | **-** | **-** | **-** | **-** |
| **1170** | **monomethylamine:corrinoid methyltransferase** | **30.4** | **0** | **70.51** | [**WP_145842117.1**](https://www.ncbi.nlm.nih.gov/protein/WP_145842117.1?report=genbank&log$=prottop&blast_rank=1&RID=WXHBA256015) | **0** | **68.74** | [**AMN47925.1**](https://www.ncbi.nlm.nih.gov/protein/AMN47925.1?report=genbank&log$=prottop&blast_rank=2&RID=WXHBA256015) | **-** | **-** | **-** | **-** | **-** | **-** | **-** | **-** | **-** | **-** | **-** |
| **1171** | **serine hydroxymethyltransferase** | **27.9** | **0** | **90.76** | [**WP_145842118.1**](https://www.ncbi.nlm.nih.gov/protein/WP_145842118.1?report=genbank&log$=prottop&blast_rank=1&RID=WXHE04GE015) | **0** | **73.8** | [**WP_066921018.1**](https://www.ncbi.nlm.nih.gov/protein/WP_066921018.1?report=genbank&log$=prottop&blast_rank=2&RID=WXHE04GE015) | **0** | **63.94** | [**SMB23984.1**](https://www.ncbi.nlm.nih.gov/protein/SMB23984.1?report=genbank&log$=prottop&blast_rank=7&RID=WXHE04GE015) | ***C. testosteroni* ATCC 11996** | **0** | **63.22** | [**EHN65544.1**](https://www.ncbi.nlm.nih.gov/protein/EHN65544.1?report=genbank&log$=prottop&blast_rank=6&RID=WXHE04GE015) | ***M. tuberculosis* H37Rv** | **4E-155** | **55.85** | [**P9WGI9.2**](https://www.ncbi.nlm.nih.gov/protein/P9WGI9.2?report=genbank&log$=prottop&blast_rank=8&RID=WXHE04GE015) |
| **1172** | **methionine synthase** | **28.8** | **0** | **95.62** | [**WP_145842119.1**](https://www.ncbi.nlm.nih.gov/protein/WP_145842119.1?report=genbank&log$=prottop&blast_rank=1&RID=WXHHK0C1014) | **0** | **59.04** | [**WP_066919687.1**](https://www.ncbi.nlm.nih.gov/protein/WP_066919687.1?report=genbank&log$=prottop&blast_rank=5&RID=WXHHK0C1014) | **0** | **75.91** | [**WP_067170133.1**](https://www.ncbi.nlm.nih.gov/protein/WP_067170133.1?report=genbank&log$=prottop&blast_rank=3&RID=WXHHK0C1014) | ***Pseudomonas* sp. Chol1** | **0** | **63.65** | [**WP_008567848.1**](https://www.ncbi.nlm.nih.gov/protein/WP_008567848.1?report=genbank&log$=prottop&blast_rank=4&RID=WXHHK0C1014) | ***M. tuberculosis* H37Rv** | **1E-165** | **31.96** | [**NP_216640.1**](https://www.ncbi.nlm.nih.gov/protein/NP_216640.1?report=genbank&log$=prottop&blast_rank=7&RID=WXHHK0C1014) |
| 1173 | SDR family oxidoreductase | 27.3 | 2E-144 | 58.55 | [WP_145841128.1](https://www.ncbi.nlm.nih.gov/protein/WP_145841128.1?report=genbank&log$=prottop&blast_rank=1&RID=WXHPW9U5015) | - | - | - | - | - | - | *C. testosteroni* ATCC 11996 | 1E-62 | 40.88 | [EHN64501.1](https://www.ncbi.nlm.nih.gov/protein/EHN64501.1?report=genbank&log$=prottop&blast_rank=2&RID=WXHPW9U5015) | - | - | - | - |
| 1174 | glucose-1-dehydrogenase | 28.6 | 4E-89 | 52.12 | [WP_145841129.1](https://www.ncbi.nlm.nih.gov/protein/WP_145841129.1?report=genbank&log$=prottop&blast_rank=1&RID=WXHUPKWZ014) | 1E-71 | 45.85 | [WP_066917873.1](https://www.ncbi.nlm.nih.gov/protein/WP_066917873.1?report=genbank&log$=prottop&blast_rank=4&RID=WXHUPKWZ014) | - | - | - | *Pseudomonas* sp. Chol1 | 2E-80 | 47.22 | [WP_037020118.1](https://www.ncbi.nlm.nih.gov/protein/WP_037020118.1?report=genbank&log$=prottop&blast_rank=2&RID=WXHUPKWZ014) | - | - | - | - |
| 1175 | DNA-binding protein | 25.9 | 4E-137 | 59.44 | [WP_145840687.1](https://www.ncbi.nlm.nih.gov/protein/WP_145840687.1?report=genbank&log$=prottop&blast_rank=1&RID=WXJ89T15015) | 2E-83 | 43.89 | [WP_066917824.1](https://www.ncbi.nlm.nih.gov/protein/WP_066917824.1?report=genbank&log$=prottop&blast_rank=5&RID=WXJ89T15015) | 5.00E-56 | 33.33 | [WP_067169255.1](https://www.ncbi.nlm.nih.gov/protein/WP_067169255.1?report=genbank&log$=prottop&blast_rank=7&RID=WXJ89T15015) | *C. testosteroni* ATCC 11996 | 4E-100 | 46.15 | [EHN64439.1](https://www.ncbi.nlm.nih.gov/protein/EHN64439.1?report=genbank&log$=prottop&blast_rank=2&RID=WXJ89T15015) | *Rhodococcus jostii* RHA1 | 1E-66 | 35.38 | [ABG97603.1](https://www.ncbi.nlm.nih.gov/protein/ABG97603.1?report=genbank&log$=prottop&blast_rank=6&RID=WXJ89T15015) |
| 1176 | - | - | - | - | - | - | - | - | - | - | - | - | - | - | - | - | - | - | - |
| 1177 | acyl dehydratase | 24.8 | 3E-61 | 61.43 | [WP_145840689.1](https://www.ncbi.nlm.nih.gov/protein/WP_145840689.1?report=genbank&log$=prottop&blast_rank=1&RID=X59KRE5P015) | 2E-49 | 56.39 | [WP_066917826.1](https://www.ncbi.nlm.nih.gov/protein/WP_066917826.1?report=genbank&log$=prottop&blast_rank=2&RID=X59KRE5P015) | - | - | - | - | - | - | - | - | - | - | - |
| 1178 | lipid transfer protein | 26.2 | 0 | 79.33 | [WP_145840690.1](https://www.ncbi.nlm.nih.gov/protein/WP_145840690.1?report=genbank&log$=prottop&blast_rank=1&RID=X59MK130015) | 0 | 67.95 | [WP_066917827.1](https://www.ncbi.nlm.nih.gov/protein/WP_066917827.1?report=genbank&log$=prottop&blast_rank=4&RID=X59MK130015) | 1.00E-125 | 48.94 | [SMB21395.1](https://www.ncbi.nlm.nih.gov/protein/SMB21395.1?report=genbank&log$=prottop&blast_rank=11&RID=X59MK130015) | *C. testosteroni* ATCC 11996 | 0 | 65.54 | [EHN64436.1](https://www.ncbi.nlm.nih.gov/protein/EHN64436.1?report=genbank&log$=prottop&blast_rank=2&RID=X59MK130015) | *Rhodococcus jostii* RHA1 | 3E-171 | 60.31 | [ABG96274.1](https://www.ncbi.nlm.nih.gov/protein/ABG96274.1?report=genbank&log$=prottop&blast_rank=5&RID=X59MK130015) |
| 1179 | long-chain-acyl-CoA synthetase | 21.9 | 0 | 55.44 | [WP_145840692.1](https://www.ncbi.nlm.nih.gov/protein/WP_145840692.1?report=genbank&log$=prottop&blast_rank=1&RID=X59R951M014) | 0 | 47.99 | [WP_066917822.1](https://www.ncbi.nlm.nih.gov/protein/WP_066917822.1?report=genbank&log$=prottop&blast_rank=7&RID=X59R951M014) | 0 | 51.99 | [WP_067169241.1](https://www.ncbi.nlm.nih.gov/protein/WP_067169241.1?report=genbank&log$=prottop&blast_rank=2&RID=X59R951M014) | *Pseudomonas* sp. Chol1 | 0 | 51.42 | [EHN64434.1](https://www.ncbi.nlm.nih.gov/protein/EHN64434.1?report=genbank&log$=prottop&blast_rank=5&RID=X59R951M014) | *M. tuberculosis* H37Rv | 1E-117 | 36.33 | [NP_215722.1](https://www.ncbi.nlm.nih.gov/protein/NP_215722.1?report=genbank&log$=prottop&blast_rank=8&RID=X59R951M014) |
| 1180 | tetR/acrR family transcriptional regulator | 22 | - | - | - | - | - | - | - | - | - | - | - | - | - | - | - | - | - |
| 1181 | tonB-dependent receptor | 31.2 | 0 | 49.62 | [WP_145842586.1](https://www.ncbi.nlm.nih.gov/protein/WP_145842586.1?report=genbank&log$=prottop&blast_rank=1&RID=X59Z2R9J014) | 0 | 40.87 | [AMN46221.1](https://www.ncbi.nlm.nih.gov/protein/AMN46221.1?report=genbank&log$=prottop&blast_rank=9&RID=X59Z2R9J014) | 0 | 44.39 | [SMB26750.1](https://www.ncbi.nlm.nih.gov/protein/SMB26750.1?report=genbank&log$=prottop&blast_rank=6&RID=X59Z2R9J014) | - | - | - | - | - | - | - | - |
| 1182 | - | - | - | - | - | - | - | - | - | - | - | - | - | - | - | - | - | - | - |
| 1183 | tetR family transcriptional regulator | 23.3 | 6E-68 | 45.45 | [WP_145842136.1](https://www.ncbi.nlm.nih.gov/protein/WP_145842136.1?report=genbank&log$=prottop&blast_rank=1&RID=X5A4DEKF014) | 6E-52 | 43.01 | [AMN47929.1](https://www.ncbi.nlm.nih.gov/protein/AMN47929.1?report=genbank&log$=prottop&blast_rank=2&RID=X5A4DEKF014) | - | - | - | - | - | - | - | - | - | - | - |
| **1184** | **methionine adenosyltransferase** | **29.5** | **0** | **98.71** | [**WP_145842135.1**](https://www.ncbi.nlm.nih.gov/protein/WP_145842135.1?report=genbank&log$=prottop&blast_rank=1&RID=X5A55ZWR014) | **0** | **76.8** | [**WP_066918246.1**](https://www.ncbi.nlm.nih.gov/protein/WP_066918246.1?report=genbank&log$=prottop&blast_rank=3&RID=X5A55ZWR014) | **0** | **69.68** | [**SMB22269.1**](https://www.ncbi.nlm.nih.gov/protein/SMB22269.1?report=genbank&log$=prottop&blast_rank=6&RID=X5A55ZWR014) | ***C. testosteroni* ATCC 11996** | **0** | **75.72** | [**EHN66722.1**](https://www.ncbi.nlm.nih.gov/protein/EHN66722.1?report=genbank&log$=prottop&blast_rank=4&RID=X5A55ZWR014) | ***Rhodococcus jostii* RHA1** | **1E-156** | **57.43** | [**Q0S0L4.1**](https://www.ncbi.nlm.nih.gov/protein/Q0S0L4.1?report=genbank&log$=prottop&blast_rank=8&RID=X5A55ZWR014) |
| **1185** | **adenosylhomocysteinase** | **29.2** | **0** | **97.27** | [**WP_145842134.1**](https://www.ncbi.nlm.nih.gov/protein/WP_145842134.1?report=genbank&log$=prottop&blast_rank=1&RID=X5A7BVHP015) | **0** | **83.76** | [**WP_066922686.1**](https://www.ncbi.nlm.nih.gov/protein/WP_066922686.1?report=genbank&log$=prottop&blast_rank=5&RID=X5A7BVHP015) | **0** | **86.11** | [**WP_067170411.1**](https://www.ncbi.nlm.nih.gov/protein/WP_067170411.1?report=genbank&log$=prottop&blast_rank=3&RID=X5A7BVHP015) | ***C. testosteroni* ATCC 11996** | **0** | **78.32** | [**EHN67178.1**](https://www.ncbi.nlm.nih.gov/protein/EHN67178.1?report=genbank&log$=prottop&blast_rank=6&RID=X5A7BVHP015) | ***Rhodococcus jostii* RHA1** | **0** | **58.73** | [**ABG98099.1**](https://www.ncbi.nlm.nih.gov/protein/ABG98099.1?report=genbank&log$=prottop&blast_rank=7&RID=X5A7BVHP015) |
| 1186 | - | - | - | - | - | - | - | - | - | - | - | - | - | - | - | - | - | - | - |
| **1187** | **methylenetetrahydrofolate reductase [NAD(P)H]** | **25.5** | **0** | **93.48** | [**WP_145842133.1**](https://www.ncbi.nlm.nih.gov/protein/WP_145842133.1?report=genbank&log$=prottop&blast_rank=1&RID=X5ABS128014) | **2E-65** | **38.77** | [**WP_066919685.1**](https://www.ncbi.nlm.nih.gov/protein/WP_066919685.1?report=genbank&log$=prottop&blast_rank=6&RID=X5ABS128014) | **9.00E-125** | **60.59** | [**WP_067170408.1**](https://www.ncbi.nlm.nih.gov/protein/WP_067170408.1?report=genbank&log$=prottop&blast_rank=3&RID=X5ABS128014) | ***Pseudomonas* sp. Chol1** | **2E-112** | **55.27** | [**WP_008568480.1**](https://www.ncbi.nlm.nih.gov/protein/WP_008568480.1?report=genbank&log$=prottop&blast_rank=4&RID=X5ABS128014) | ***Rhodococcus jostii* RHA1** | **8E-50** | **35.21** | [**ABG92932.1**](https://www.ncbi.nlm.nih.gov/protein/ABG92932.1?report=genbank&log$=prottop&blast_rank=7&RID=X5ABS128014) |
| 1188 | tetR family transcriptional regulator | 5.5 | 2E-142 | 87.1 | [WP_145842132.1](https://www.ncbi.nlm.nih.gov/protein/WP_145842132.1?report=genbank&log$=prottop&blast_rank=1&RID=X5APS037015) | - | - | - | - | - | - | - | - | - | - | - | - | - | - |
| 1189 | 7,8-didemthyl-8-hydroxy.5.deazariboflavin synthase subunit CofH | - | 2E-106 | 85.65 | [WP_145842131.1](https://www.ncbi.nlm.nih.gov/protein/WP_145842131.1?report=genbank&log$=prottop&blast_rank=1&RID=X5ASJGG5015) | 7E-59 | 70.14 | [WP_066917828.1](https://www.ncbi.nlm.nih.gov/protein/WP_066917828.1?report=genbank&log$=prottop&blast_rank=3&RID=X5ASJGG5015) | - | - | - | - | - | - | - | - | - | - | - |
| 1190 | 7,8-didemthyl-8-hydroxy.5.deazariboflavin synthase subunit CofH | 18.9 | 0 | 96.19 | [WP_145842131.1](https://www.ncbi.nlm.nih.gov/protein/WP_145842131.1?report=genbank&log$=prottop&blast_rank=1&RID=X5AURJ2G014) | 0 | 64.55 | [WP_066917828.1](https://www.ncbi.nlm.nih.gov/protein/WP_066917828.1?report=genbank&log$=prottop&blast_rank=3&RID=X5AURJ2G014) | 0 | 62.6 | [SMB22493.1](https://www.ncbi.nlm.nih.gov/protein/SMB22493.1?report=genbank&log$=prottop&blast_rank=5&RID=X5AURJ2G014) | - | - | - | - | *Rhodococcus jostii* RHA1 | 0 | 47.68 | [ABH00688.1](https://www.ncbi.nlm.nih.gov/protein/ABH00688.1?report=genbank&log$=prottop&blast_rank=7&RID=X5AURJ2G014) |
| 1191 | cobyrinate a,c-diamide synthase | 24.9 | 0 | 92.82 | [WP_145842137.1](https://www.ncbi.nlm.nih.gov/protein/WP_145842137.1?report=genbank&log$=prottop&blast_rank=1&RID=X5AY1H8V014) | - | - | - | - | - | - | *Pseudomonas* sp. Chol1 | 7E-171 | 59.39 | [WP_008568181.1](https://www.ncbi.nlm.nih.gov/protein/WP_008568181.1?report=genbank&log$=prottop&blast_rank=2&RID=X5AY1H8V014) | - | - | - | - |
| 1192 | cob(1)yrinic acid a,c-diamide adenosyltransferase | 25.5 | 1E-142 | 93.66 | [WP_145842138.1](https://www.ncbi.nlm.nih.gov/protein/WP_145842138.1?report=genbank&log$=prottop&blast_rank=1&RID=X5B4120S014) | - | - | - | - | - | - | *Pseudomonas* sp. Chol1 | 6E-91 | 63 | [WP_008568182.1](https://www.ncbi.nlm.nih.gov/protein/WP_008568182.1?report=genbank&log$=prottop&blast_rank=2&RID=X5B4120S014) | - | - | - | - |
| 1193 | hypothetical protein | - | 5E-105 | 88.82 | [WP_145842139.1](https://www.ncbi.nlm.nih.gov/protein/WP_145842139.1?report=genbank&log$=prottop&blast_rank=1&RID=X5B6MEVA014) | - | - | - | - | - | - | *Pseudomonas* sp. Chol1 | 6E-91 | 63 | [WP_008568182.1](https://www.ncbi.nlm.nih.gov/protein/WP_008568182.1?report=genbank&log$=prottop&blast_rank=2&RID=X5B4120S014) | - | - | - | - |
| 1194 | tonB-dependent vitamin B12 receptor | 27.3 | 0 | 95.51 | [WP_145842140.1](https://www.ncbi.nlm.nih.gov/protein/WP_145842140.1?report=genbank&log$=prottop&blast_rank=1&RID=X5BFM7RV015) | - | - | - | - | - | - | *C. testosteroni* ATCC 11996 | 5E-142 | 40.26 | [EHN64112.1](https://www.ncbi.nlm.nih.gov/protein/EHN64112.1?report=genbank&log$=prottop&blast_rank=3&RID=X5BFM7RV015) | - | - | - | - |
| 1195 | glycosyl hydrolase | - | 0 | 95.22 | [WP_145842141.1](https://www.ncbi.nlm.nih.gov/protein/WP_145842141.1?report=genbank&log$=prottop&blast_rank=1&RID=X5CDCVYY014) | - | - | - | - | - | - | *Pseudomonas* sp. Chol1 | 1E-53 | 37.08 | [EKM95394.1](https://www.ncbi.nlm.nih.gov/protein/EKM95394.1?report=genbank&log$=prottop&blast_rank=2&RID=X5CDCVYY014) | - | - | - | - |
| 1196 | MMPL family transporter | - | 0 | 96.46 | [WP_145842142.1](https://www.ncbi.nlm.nih.gov/protein/WP_145842142.1?report=genbank&log$=prottop&blast_rank=1&RID=X5CJH4B5014) | - | - | - | 1.00E-166 | 37.76 | [SMB32103.1](https://www.ncbi.nlm.nih.gov/protein/SMB32103.1?report=genbank&log$=prottop&blast_rank=3&RID=X5CJH4B5014) | *Pseudomonas* sp. Chol1 | 6E-170 | 36.88 | [WP_008567110.1](https://www.ncbi.nlm.nih.gov/protein/WP_008567110.1?report=genbank&log$=prottop&blast_rank=2&RID=X5CJH4B5014) | - | - | - | - |
| 1197 | hypothetical protein | 18.5 | 0 | 97.19 | [WP_145842143.1](https://www.ncbi.nlm.nih.gov/protein/WP_145842143.1?report=genbank&log$=prottop&blast_rank=1&RID=X5CNHJEU014) | - | - | - | - | - | - | - | - | - | - | - | - | - | - |
| 1198 | hypothetical protein | - | 0 | 98.41 | [WP_145843522.1](https://www.ncbi.nlm.nih.gov/protein/WP_145843522.1?report=genbank&log$=prottop&blast_rank=1&RID=X5CT2SX5015) | - | - | - | 2.00E-77 | 36.08 | [WP_083522864.1](https://www.ncbi.nlm.nih.gov/protein/WP_083522864.1?report=genbank&log$=prottop&blast_rank=8&RID=X5CT2SX5015) | *Pseudomonas* sp. Chol1 | 6E-104 | 38.8 | [WP_008567041.1](https://www.ncbi.nlm.nih.gov/protein/WP_008567041.1?report=genbank&log$=prottop&blast_rank=2&RID=X5CT2SX5015) | - | - | - | - |
| 1199 | tonB-dependent vitamin B12 receptor | 27.4 | 0 | 94.88 | [WP_145842144.1](https://www.ncbi.nlm.nih.gov/protein/WP_145842144.1?report=genbank&log$=prottop&blast_rank=1&RID=X5CVRAV5015) | - | - | - | - | - | - | *C. testosteroni* ATCC 11996 | 7E-111 | 35.32 | [EHN64112.1](https://www.ncbi.nlm.nih.gov/protein/EHN64112.1?report=genbank&log$=prottop&blast_rank=3&RID=X5CVRAV5015) | - | - | - | - |
| 1200 | iron chelate uptake ABC transporter family permease subunit | 15.8 | 0 | 98.48 | [WP_145842145.1](https://www.ncbi.nlm.nih.gov/protein/WP_145842145.1?report=genbank&log$=prottop&blast_rank=1&RID=X5CZ3H28014) | 3E-86 | 58.36 | [AMN46142.1](https://www.ncbi.nlm.nih.gov/protein/AMN46142.1?report=genbank&log$=prottop&blast_rank=4&RID=X5CZ3H28014) | 5.00E-107 | 68.2 | [SMB21068.1](https://www.ncbi.nlm.nih.gov/protein/SMB21068.1?report=genbank&log$=prottop&blast_rank=3&RID=X5CZ3H28014) | *C. testosteroni* ATCC 11996 | 3E-49 | 40.71 | [EHN64062.1](https://www.ncbi.nlm.nih.gov/protein/EHN64062.1?report=genbank&log$=prottop&blast_rank=6&RID=X5CZ3H28014) | - | - | - | - |
| 1201 | iron(III) dicitrate transport ATP-binding protein | 23.2 | 1E-176 | 95.7 | [WP_145842146.1](https://www.ncbi.nlm.nih.gov/protein/WP_145842146.1?report=genbank&log$=prottop&blast_rank=1&RID=X5D3AH9Z015) | 1E-76 | 51.57 | [WP_066922670.1](https://www.ncbi.nlm.nih.gov/protein/WP_066922670.1?report=genbank&log$=prottop&blast_rank=4&RID=X5D3AH9Z015) | 9.00E-97 | 59.61 | [SMB21069.1](https://www.ncbi.nlm.nih.gov/protein/SMB21069.1?report=genbank&log$=prottop&blast_rank=3&RID=X5D3AH9Z015) | - | - | - | - | - | - | - | - |
| 1202 | cobric acid synthase | 24.8 | 0 | 85.71 | [WP_145842147.1](https://www.ncbi.nlm.nih.gov/protein/WP_145842147.1?report=genbank&log$=prottop&blast_rank=1&RID=X5D74PZU014) | 1E-129 | 46.97 | [WP_066922455.1](https://www.ncbi.nlm.nih.gov/protein/WP_066922455.1?report=genbank&log$=prottop&blast_rank=5&RID=X5D74PZU014) | - | - | - | *Pseudomonas* sp. Chol1 | 9E-165 | 65.32 | [EKM96535.1](https://www.ncbi.nlm.nih.gov/protein/EKM96535.1?report=genbank&log$=prottop&blast_rank=3&RID=X5D74PZU014) | *Rhodococcus jostii* RHA1 | 3E-135 | 47.28 | [Q0S258.1](https://www.ncbi.nlm.nih.gov/protein/Q0S258.1?report=genbank&log$=prottop&blast_rank=4&RID=X5D74PZU014) |
| 1203 | lipoyl synthase | 2.2 | 0 | 96.56 | [WP_145842148.1](https://www.ncbi.nlm.nih.gov/protein/WP_145842148.1?report=genbank&log$=prottop&blast_rank=1&RID=X5DB06Y2014) | 2E-88 | 48.33 | [AMN46719.1](https://www.ncbi.nlm.nih.gov/protein/AMN46719.1?report=genbank&log$=prottop&blast_rank=6&RID=X5DB06Y2014) | 5.00E-177 | 77.41 | [WP_067171077.1](https://www.ncbi.nlm.nih.gov/protein/WP_067171077.1?report=genbank&log$=prottop&blast_rank=3&RID=X5DB06Y2014) | *C. testosteroni* ATCC 11996 | 2E-158 | 71.58 | [EHN62924.1](https://www.ncbi.nlm.nih.gov/protein/EHN62924.1?report=genbank&log$=prottop&blast_rank=4&RID=X5DB06Y2014) | *M. tuberculosis* H37Rv | 1E-81 | 43.67 | [5EXI_A](https://www.ncbi.nlm.nih.gov/protein/5EXI_A?report=genbank&log$=prottop&blast_rank=9&RID=X5DB06Y2014) |
