# Supplementary material for: Channeling C1 Metabolism toward S-Adenosylmethionine-Dependent Conversion of Estrogens to Androgens in Estrogen-Degrading Bacteria
Source: mBio. 2020 Aug 25;11(4):e01259-20. doi: 10.1128/mBio.01259-20 (PMC7448270; doi:10.1128/mBio.01259-20)
Supplement: TABLE S4 [file mBio.01259-20-st004.docx]

**Table S4.** Differential abundances of gene products involved in C1-metabolism/SAM regeneration in Denitratisoma oestradiolicum. For each predicted enzyme (based on e-values ≤ 1 e^-50^ and amino acid sequence identities ≥ 30%), two gene copies are present in the genome. The log2 abundances of each gene product are given after proteome analyses of estradiol vs acetate grown cells. Only the genes DENOEST_v1_11165-1187 are higher abundant in estradiol-grown cells. n.d. = not detectable.

| Predicted gene function | Gene accession number [DENOEST_v1_XXXX] | Log2/fc [estradiol/acetate] |
| --- | --- | --- |
| Serine hydroxymethyltransferase | 1171 | **+ 27.9** |
|  | 3564 | **- 0.5** |
| Methionine synthase | 1172 | **+ 28.8** |
|  | 3243 | **+ 1.4** |
| L-methionineadenosyltransferase | 1184 | **+ 29.5** |
|  | 3406 | **- 0.9** |
| S-adenosylhomocysteinase | 1185 | **+ 29.2** |
|  | 3410 | **n.d.** |
| N^5^,N^10^-methylene-THF reductase | 1187 | **+ 25.5** |
|  | 3412 | **n.d.** |
| Adenosine kinase | 1165 | **+ 26.3** |
|  | 3508 | **- 1.3** |
